# Promoting alcohol treatment engagement post-hospitalization with brief intervention, medications and CBT4CBT: protocol for a randomized clinical trial in a diverse patient population

**DOI:** 10.1186/s13722-023-00407-9

**Published:** 2023-09-19

**Authors:** E. Jennifer Edelman, Oscar F. Rojas-Perez, Charla Nich, Joanne Corvino, Tami Frankforter, Derrick Gordon, Ayana Jordan, Manuel Paris, Jr, Melissa B. Weimer, Brian T. Yates, Emily C. Williams, Brian D. Kiluk

**Affiliations:** 1 Department of Internal Medicine, Yale School of Medicine, 367 Cedar Street, ES Harkness Memorial Hall, Suite 401, New Haven, CT 06510 USA; 2 Yale Program in Addiction Medicine, Yale School of Medicine, New Haven, CT USA; 3 Department of Social and Behavioral Sciences, Yale School of Medicine, New Haven, CT USA; 4 Department of Psychiatry, Yale School of Medicine, New Haven, CT USA; 5 The Consultation Center, New Haven, CT USA; 6 Department of Psychiatry, NYU Langone Health, New York, NY USA; 7 Hispanic Clinic, Connecticut Mental Health Center, New Haven, CT USA; 8 Department of Chronic Disease Epidemiology, Yale School of Public Health, New Haven, CT USA; 9 Department of Psychology, American University, Washington, DC USA; 10 Department of Health Systems and Population Health, University of Washington School of Public Health, Seattle, WA USA; 11 Health Services Research and Development Seattle-Denver Center of Innovation for Veteran-Centered and Value-Driven Care, Veterans Health Administration (VA), Seattle, WA USA

**Keywords:** Alcohol use disorder, Implementation science, Clinical trial protocol, Racialized and ethnic minoritized communities

## Abstract

**Background:**

Alcohol use disorder (AUD) commonly causes hospitalization, particularly for individuals disproportionately impacted by structural racism and other forms of marginalization. The optimal approach for engaging hospitalized patients with AUD in treatment post-hospital discharge is unknown. We describe the rationale, aims, and protocol for *Project ENHANCE* (ENhancing Hospital-initiated Alcohol TreatmeNt to InCrease Engagement), a clinical trial testing increasingly intensive approaches using a hybrid type 1 effectiveness-implementation approach.

**Methods:**

We are randomizing English and/or Spanish-speaking individuals with untreated AUD (n = 450) from a large, urban, academic hospital in New Haven, CT to: (1) Brief Negotiation Interview (with referral and telephone booster) alone (BNI), (2) BNI plus facilitated initiation of medications for alcohol use disorder (BNI + MAUD), or (3) BNI + MAUD + initiation of computer-based training for cognitive behavioral therapy (CBT4CBT, BNI + MAUD + CBT4CBT). Interventions are delivered by Health Promotion Advocates. The primary outcome is AUD treatment engagement 34 days post-hospital discharge. Secondary outcomes include AUD treatment engagement 90 days post-discharge and changes in self-reported alcohol use and phosphatidylethanol. Exploratory outcomes include health care utilization. We will explore whether the effectiveness of the interventions on AUD treatment engagement and alcohol use outcomes differ across and within racialized and ethnic groups, consistent with disproportionate impacts of AUD. Lastly, we will conduct an implementation-focused process evaluation, including individual-level collection and statistical comparisons between the three conditions of costs to providers and to patients, cost-effectiveness indices (effectiveness/cost ratios), and cost–benefit indices (benefit/cost ratios, net benefit [benefits minus costs). Graphs of individual- and group-level effectiveness x cost, and benefits x costs, will portray relationships between costs and effectiveness and between costs and benefits for the three conditions, in a manner that community representatives also should be able to understand and use.

**Conclusions:**

Project ENHANCE is expected to generate novel findings to inform future hospital-based efforts to promote AUD treatment engagement among diverse patient populations, including those most impacted by AUD.

*Clinical Trial Registration*: Clinicaltrials.gov identifier: NCT05338151.

**Supplementary Information:**

The online version contains supplementary material available at 10.1186/s13722-023-00407-9.

## Contributions to the literature

- Acute medical hospitalization may be an important setting for providing treatment of untreated alcohol use disorder (AUD), particularly for those at greatest risk for adverse consequences of AUD;
- The optimal strategy for engaging patients with untreated AUD to promote engagement in treatment post-hospital discharge is currently unknown;
- Brief Negotiation Interview with facilitated initiation of medications for AUD and initiation of computer-based training for cognitive behavioral therapy (CBT4CBT) is expected to be an effective strategy for promoting AUD treatment engagement post-hospital discharge;
- Data regarding factors relevant for initiating AUD treatment in the hospital setting are needed to inform real-world implementation: this should be facilitated by data collected at the individual level and analyzed statistically comparing costs and cost-effectiveness of in-hospital AUD treatment strategies.

## Introduction

**Fig. 1:**
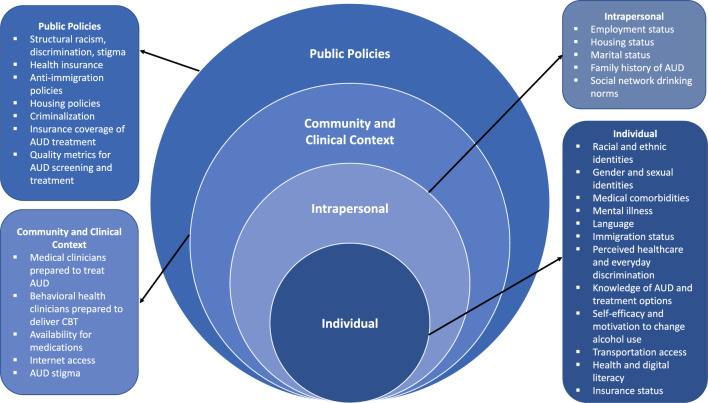
Factors that drive alcohol use disorder and may moderate effectiveness of interventions on promoting treatment engagement: an application of the socioecological model [100–102]

Alcohol use disorder (AUD) is a major cause of morbidity and mortality [1], yet typically left untreated [2]. Among the 29.5 million adults with AUD in 2021, only 4.6% had received any treatment (i.e., behavioral or medication) in the past year and less than 2% took a Food and Drug Administration (FDA)-approved medication for AUD (MAUD) [2]. Structural racism—the totality of ways racism is pervasively and deeply embedded in systems, laws, and written or unwritten policies—and related social determinants of health (SDOH, e.g., inequitable access to education; food insecurity; and discrimination) that are enacted through markers of race and ethnicity may drive alcohol use and interfere with access to and potential benefits of AUD treatment on multiple levels (Fig. 1). Extant research has demonstrated that AUD prevalence and treatment rates differ across racial and ethnic groups and these differences may be the direct result of structural racism and SDOH [3, 4]. Strategies in relevant “touchpoints” are needed that overcome barriers imposed by structural racism and SDOH to address AUD across populations, particularly minoritized racial and ethnic populations.

Acute medical hospitalization may provide such an untapped opportunity to link diverse individuals with AUD to treatment [5]. First, alcohol-associated hospitalizations are common [6, 7] and represent a “reachable moment” when a range of behavioral and medication-based treatments may be initiated and supported by multidisciplinary hospital-based clinicians and staff [8, 9]. Second, hospitalization may offer a unique opportunity to reach minoritized racial and ethnic populations as they have greater rates of alcohol-related hospitalizations relative to White individuals [10], and, importantly, are less likely to receive routine care elsewhere [11]. Third, patients may be introduced to evidence-based digital interventions to address AUD [12] that could continue upon hospital discharge and address barriers to treatment (e.g., variability in community-based access to behavioral health treatment).

To date, few studies have sought to identify the optimal strategy for linking patients to AUD treatment during acute hospitalization [5, 13]. Though prior studies have focused on evaluating different medication treatment options [14–19], to our knowledge, no studies have compared the impact of brief counseling interventions with the addition of MAUD with and without digital interventions to promote post-hospitalization treatment engagement among all individuals with AUD. Also, no studies have focused on understanding differential effectiveness of treatment engagement strategies across racialized and ethnic groups who experience enhanced adverse impacts of AUD and have unique barriers to AUD treatment. To address this important gap, we are conducting *Project ENHANCE* (ENhancing Hospital-initiated Alcohol TreatmeNt to InCrease Engagement), a three-arm randomized clinical trial with a hybrid type 1 effectiveness-implementation approach that also reports and statistically compares between conditions the provider and patient costs, indices of cost-effectiveness, and indices of cost–benefit with attention to the role of systemic racism.

## Methods

### Overall design

**Fig. 2:**
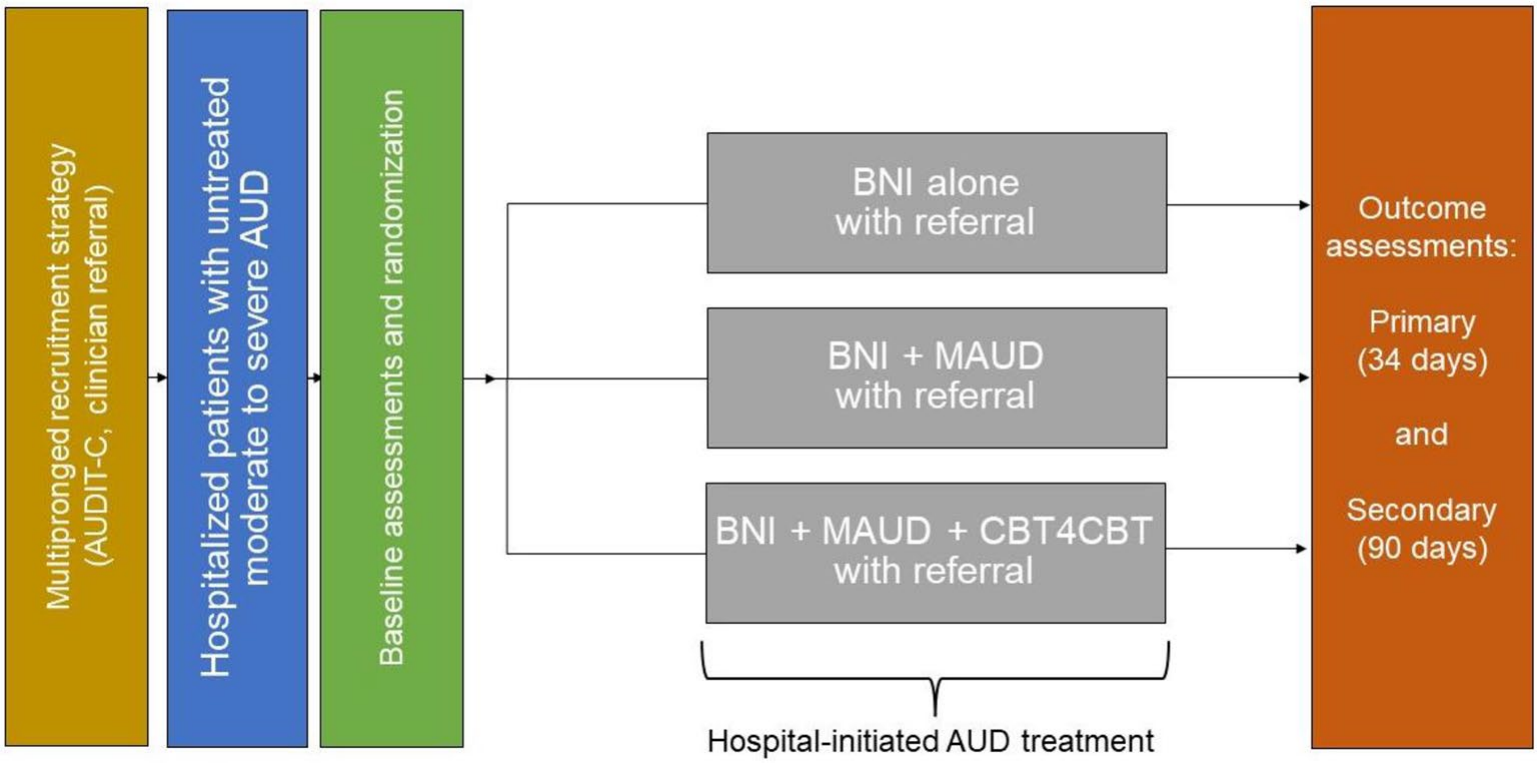
*Project ENHANCE* Protocol Overview^a^. a. All participants regardless of treatment conditions also receive telephone booster at 2 weeks. *AUD* alcohol use disorder; *BNI* brief negotiation interview; *MAUD* medications for alcohol use disorder; *CBT4CBT* computer-based training for cognitive behavioral therapy

Funded by the National Institute on Alcohol Abuse and Alcoholism, *Project ENHANCE* is a three-arm randomized clinical trial that aims to enroll a diverse sample of individuals with untreated AUD (n = 450) during their acute medical hospitalization at a large urban hospital in the US northeast. We will compare the effectiveness of three treatment engagement strategies on the primary outcome of AUD treatment engagement at the 34th day post-hospital discharge (Aim 1, Fig. 2): (1) Brief Negotiation Interview (with referral and telephone booster) alone (BNI), (2) BNI plus facilitated initiation of MAUD (BNI + MAUD), or (3) BNI + MAUD + initiation of computer-based training for cognitive behavioral therapy (CBT4CBT, BNI + MAUD + CBT4CBT). Secondary outcomes include AUD treatment engagement on the 90th day post-hospital discharge and changes in self-reported alcohol use (percentage of heavy drinking days by Timeline Followback) and the alcohol biomarker, phosphatidylethanol (PEth). Exploratory outcomes based on self-report and the electronic medical record include health care utilization, such as emergency department visits and hospital readmission. We will additionally explore whether the effectiveness of the interventions on AUD treatment engagement and alcohol use outcomes differ across and within racialized and ethnic groups based on SDOH (Aim 2). In parallel and consistent with a hybrid type 1 effectiveness-implementation approach [20], we will conduct an implementation-focused process evaluation [21] that includes costs, cost-effectiveness, and cost–benefit analyses comparing the three intervention combinations (Aim 3).

To ensure that a range of perspectives inform the study design and protocol implementation, our study team is intentionally diverse based on training (psychologists, inpatient and outpatient-based Addiction Medicine and Addiction Psychiatry physicians, health services researchers with health equity focus); race and ethnicity (Black, Hispanic, and White); gender; immigration status; sexual orientation; and lived experience with family and/or friends impacted by AUD.

### Rationale for study design

Our study design is informed by several key principles. First, while addiction screening and treatment is increasingly being provided in hospital settings, this is not uniformly done utilizing evidence-informed care by trained medical personnel. The optimal strategy to promote engagement in AUD treatment post-hospitalization is not known. This is of particular concern for racial and ethnically minoritized individuals with AUD who are known to have worsening health disparities from alcohol use due to systemic inequities [3, 11, 22, 23]. Second, hospitalization may be an opportunity to minimize the impact of common drivers of health inequities and reach particularly vulnerable patient populations (e.g., individuals disproportionately impacted by structural racism and its manifestations, individuals experiencing houselessness) and others who may not access routine outpatient care. Since the mean length of a hospital stay for a patient with a principal alcohol-related disorder is nearly 5 days [24], there is a potential opportunity to engage patients in various treatments without the challenges (e.g., transportation) in outpatient settings. Third, best practice recommendations for treating AUD include an evidence-based behavioral therapy, such as CBT, with MAUD [25]. Unfortunately, efforts to initiate these treatments together during acute medical hospitalization are lacking [26]. Fourth, access to high-quality CBT is limited in community treatment settings due to the cost of training and supervision required [27], but may be overcome by digitally-delivered approaches that seem inexpensive to deliver in hospital settings and serve to overcome barriers related to treatment availability [28, 29]; concerns of discrimination or stigma [30, 31]; and poverty and environmental violence that may impede in-person treatment engagement [32, 33]. CBT4CBT, is a proven, scalable platform for addressing AUD that is available in English and Spanish; does not require literacy or high levels of proficiency with computers or technology and can be readily delivered via tablets; and is beneficial across diverse populations offering a potentially useful strategy to help individuals with AUD develop necessary skills to reduce alcohol use post-hospital discharge [34, 35]. Fifth, there are limited data characterizing the prevalence of SDOH, many of which are indicators of structural racism, among patients hospitalized with AUD [36] and few studies have examined the moderating effects of racialized identity, ethnicity, and SDOH on AUD treatment outcomes [37]. Sixth, while there is growing interest in the alcohol biomarker phosphatidylethanol (PEth) for detecting unhealthy alcohol use [38], there is a lack of data on factors that impact PEth among medically complex patients in the context of acute illness [39]. Lastly, to inform future implementation efforts beyond the research context, it is important to collect data on factors that impact trial implementation guided by implementation science principles [20].

### Study aims and hypotheses

Among 450 hospitalized individuals with untreated moderate to severe AUD (by Diagnostic and Statistical Manual-5 [DSM-5] criteria), our aims are to evaluate the effectiveness of hospital-initiated BNI vs. BNI + MAUD vs. BNI + MAUD + CBT4CBT on AUD treatment engagement at 34 days (primary outcome) and 90 days post-discharge; changes in alcohol use by self-report and biomarker over the 90 day period; and (exploratory) health care utilization, including Emergency Department visits and hospital readmission (Aim 1). We hypothesize that BNI + MAUD + CBT4CBT will be more effective than BNI + MAUD, which will be more effective than BNI alone as evidenced by higher rates of AUD treatment engagement, and reductions in alcohol use and urgent and emergency healthcare utilization post-discharge.

We will explore whether the effectiveness of the interventions on AUD treatment engagement and alcohol use outcomes differs across and within racial and ethnic groups and by SDOH indicators that reflect sequalae of structural racism (Aim 2). We hypothesize that there will be no difference in treatment effectiveness by race or ethnicity after adjustment of confounders but there will be differences based on SDOH.

Consistent with our prior experiences [40], we will additionally conduct an implementation-focused process evaluation [21], involving process measures, a clinician and staff survey, in addition to obtaining measures of the amounts and unit costs of resources used to implement each intervention to patients as well as health care staff. Analyses of cost, cost effectiveness, possible cost-savings benefits from reduced use of health care following the intervention, and cost–benefit for each intervention will compare individual-level provider and patient costs, indices of cost-effectiveness, and indices of cost–benefit for the three intervention combinations, as well as intervention-level indices of incremental cost-effectiveness ratios (ICERs) [41, 42] (Aim 3).

### Study context

The study is being conducted at Yale New Haven Hospital (YNHH), a large, urban teaching hospital in New Haven, CT that includes two campuses serving a diverse patient population. For example, from July 1, 2022, to June 30, 2023, 2209 unique patients were admitted to a Medicine service at YNHH who had evidence of an alcohol-related diagnosis (based on clinical orders for Clinical Institute Withdrawal Assessment—Alcohol; AUDIT-C ≥ 7; and/or alcohol-related diagnosis). Among these patients, the mean age was 54 yo (standard deviation = 14.8) and the majority were men (68%). Thirteen percent identified as Hispanic or Latino and 27% as Black. YNHH uses the *Epic®* electronic medical record and has integrated tools to promote evidence-based AUD care, including Alcohol Use Disorder Identification Test-Consumption (AUDIT-C) [43] screening prompts for all hospitalized patients and an AUD signature care pathway that is triggered for patients with evidence of a potential AUD. Hospital-based treatment services include Addiction Medicine specialty consultation care provided by the Yale Addiction Medicine Consult Service (YAMCS). This Service routinely recommends initiation of MAUD, facilitates outpatient referral, and is consulted for the minority of hospitalized patients with AUD who present with complex substance withdrawal and/or comorbid substance use disorders. All FDA approved MAUD, including injectable naltrexone, are available on the inpatient hospital formulary. Community-based treatment options for patients with AUD discharged from YNHH include a range of options for care by Addiction-certified physicians in specialty or office-based settings. Given formative work demonstrating inconsistent knowledge and adoption of evidence-based alcohol-related care among YNHH clinicians [44], we planned to conduct a clinician training prior to study launch.

### Inclusion and exclusion criteria

Inclusion and exclusion criteria are summarized in Table 1.

**Table 1:** *Project ENHANCE* Inclusion and Exclusion Criteria

Inclusion criteria: *all criteria must be met*	Exclusion criteria: *excluded if one or more of these criteria are met*
1) Are hospitalized at YNHH on a general medical ward	1) Been engaged in formal AUD treatment in the past 30 days (i.e., excluding mutual help groups, such as Alcoholics Anonymous)
2) Have an AUDIT-C score ≥ 7	2) Meet DSM-5 criteria for untreated moderate to severe opioid use disorder
3) Meet Diagnostic and Statistical Manual (DSM-5) criteria for a moderate to severe AUD (regardless of primary reason for hospitalization) consistent with clinical guidelines for MAUD initiation	
4)≥ 1 heavy drinking day in the 30 days prior to hospitalization	3) Self-reported or have urine testing confirming pregnancy, nursing, or trying to conceive
5) Are ≥ 18 years old	4) A life-threatening or unstable medical, surgical, or psychiatric condition that prohibits study participation
6) Willing to consider MAUD	5) Inability to provide ≥ 1 form of contact information
7) Are willing and able to be contacted for follow-up	6) Anticipate being unable to return for follow-up assessments for any reason, such as travel, planned procedure
	7) Inability to understand English or Spanish
	8) Currently in jail, prison or other overnight facility as required by court of law and/or is considered a prisoner under local law or is under current terms of civil commitment or guardianship

### Recruitment and randomization

In collaboration with frontline clinicians, nursing staff, and YAMCS and *Epic* reports, potentially eligible participants are identified based on: (1) frontline clinician and staff referral; (2) identified with a proactive daily *Epic* generated report as having a documented Clinical Institute Withdrawal Assessment (CIWA) for Alcohol score in the past 96 h, an AUDIT-C score ≥ 7 in the past 96 h, and/or an active AUD diagnosis on their problem list, and are hospitalized on a general medicine ward; (3) YAMCS team referral; and (4) recruitment handouts and flyers for patients admitted to the hospital. Upon confirmation from the primary care team that the patient is appropriate to approach for research, the research coordinator will approach the patient and obtain verbal permission to screen for eligibility. Individuals who have an AUDIT-C ≥ 7 will be assessed for presence of a moderate to severe AUD by the Alcohol Symptom Checklist[45, 46] followed by screening for ≥ 1 heavy drinking day, defined as any day of consumption of ≥ 5 standard drinks for men, and ≥ 4 standard drinks for women[47] in the 30 days prior to hospitalization. Individuals who are identified as meeting eligibility criteria, provide written informed consent to participate, and complete baseline assessments are randomized 1:1:1 using a computerized urn randomization program that balances probability of treatment condition based on: (1) race and ethnicity, (2) sex, (3) Spanish language preference, and (4) AUD severity [48, 49]. Randomized participants receive $75 gift card or a tablet computer (“tablet”) for completion of baseline assessments and an additional $50 gift card for completing each of the two follow-up assessments.

### Data collection protocol

All data are collected through Research Electronic Data Capture (REDCap) [50], the clinical trial management system. Screening and baseline assessments are collected by trained research coordinators to ensure participants meet eligibility criteria and to capture key predictor variables as well as potential moderators and mediators (Table 2). Follow-up assessments collect primary, secondary, and exploratory outcome data via participant assessments, electronic health record data, provider/facility confirmed treatment engagement, and biomarkers. The primary outcome of AUD treatment engagement on the 34th day following hospital discharge is consistent with the Healthcare Effectiveness Data and Information Set (HEDIS) Initiation and Engagement with Treatment (IET) measure (i.e., ≥ 2 AOD services within 34 days of the initiation visit), specified by the National Committee on Quality Assurance. It is used nationally by health plans [51, 52] and the endpoint in clinical trials focused on promoting addiction treatment from acute care settings [53–55]. Type of treatment will be classified according to the American Society of Addiction Medicine (ASAM) Criteria, verified by objective data (e.g., review of the electronic medical record, contact with the treatment facility, photo of oral naltrexone pill bottle) [56, 57]. Among those who report taking MAUD, we will assess MAUD type and self-reported adherence.

**Table 2:** *Project ENHANCE*: summary of assessments, rationale, and schedule

Domain/Instrument	Rationale	Time point		
		Baseline	34 day follow-up	90 day follow-up
**General measures, health status and other key characteristics**				
Demographic characteristics (e.g., race, ethnicity, primary language of choice)	Description, moderators	X		
Hospitalization information (i.e., reason for and days of hospitalization)	Description, moderator	X		
General life, economic, housing, and criminal justice status	Description, moderators	X		
Usual source of medical care	Description, moderator	X		
Technology access and comfort	Description, moderator	X		
Cognitive function (Montreal Cognitive Assessment [MoCA]) [103]	Description, moderator	X		X
Self-reported medical comorbidities [104]	Description	X		
Medication adherence [105–107]	Moderator	X^a^		
Post-traumatic stress disorder	Description, moderator	X		
Patient reported outcomes (PROMIS-Preference [PROPr]) [108]	Moderators, Health utility score	X^a^	X^a^	X^a^
* Project ENHANCE* Informed consent quiz	Description	X^a^		
**Alcohol-related measures**				
Alcohol use history (lifetime, past 30 days)	Description, moderator	X		
AUDIT-C [43]	Description, moderator	X		
Alcohol symptom checklist [46]	Description, moderator	X		X
Alcohol timeline followback [109]	Secondary outcome	X	X	X
Formal alcohol treatment [110] and MAUD adherence [55]	Primary outcome	X	X^b^	X^b^
Phosphatidylethanol (PEth) [111]	Secondary outcome	X	X	X
Perspectives on treatment for alcohol use disorder [112, 113]	Description, moderator	X		
Alcohol readiness ruler	Description, moderator	X^a^	X^b^	X^b^
Alcohol abstinence self-efficacy scale [114]	Mediator	X^a^	X^a^	X^a^
Short Inventory of problems-revised [115]	Secondary outcome	X^a^		X^a^
**Other substance use-related measures**				
Drug use, past 30 days and age of first use	Moderators	X		
Opioid use disorder symptoms [116]	Moderator	X		
Severity of drug use problems (ASSIST Lite) [117]	Moderator	X		X
Drug timeline followback	Moderator	X	X	X
Substance use treatment history	Description	X		
Family history	Moderator	X		
**Additional social determinants of health**				
Health disparities and safety screening tool, adapted [118, 119]	Moderators	X^a^		
Medical mistrust and discrimination assessment [120]	Moderator	X^a^		
Everyday discrimination scale [121]	Moderator	X^a^		
Perceived racial discrimination-adapted	Moderator	X^a^		
Immigration policy enforcement and related stress [122, 123]	Moderator	X^a^		
Multidimensional acculturative stress inventory for adults of Mexican origin [124]	Moderator	X^a^		
Multigroup ethnic identity measure-revised [125]	Moderator	X^a^		
**Treatment services and adverse events**				
Program and client costs (PACC-SAT) [126–128]	Treatment utilization and cost, mediator	X	X	
Adverse events (SAFTEE) [129]	Safety outcome		X	X
**Process measures**				
Project EHNANCE session completion, duration	Process	BNI and telephone booster		
Yale adherence and competence rating scale, adapted [130]	Intervention fidelity	BNI sessions only		
**Implementation-related measures**				
Cost-related data	Cost effectiveness	X	X	X
Participant satisfaction	Evaluation		X^a^	X^a,c^

^a^Indicates participant self-administered via survey

^b^Verified with objective measures at follow-up for outcome ascertainment [55, 131]

^c^Completed at 90 day assessment only if not completed at 34 day assessment

A secondary outcome of AUD treatment engagement is potential change in use and cost of health services from before the index hospitalization to a comparable period following hospital discharge. Reduction in health service use and costs would be a cost-savings outcome (i.e., benefit of the intervention) [58]. Even if these health service use and cost savings do not completely compensate for intervention costs, they could at least reduce net intervention cost. Emergency department, outpatient, and inpatient visits as well as medication before and after hospitalization will be extracted from the electronic health record for the 12 months before and following hospitalization. Intervention costs themselves will be assessed by multiplying provider and patient time spent for each patient in each intervention condition by provider and patient self-reported wage rates including employment perquisites, plus any materials, medication, and other resources used in interventions. These costs, and costs of computers and software used in CBT4CBT conditions will be assessed using methods developed in previous research published by several of the current authors [58].

### Intervention components overview

#### Brief negotiation interview with telephone booster (BNI)

All participants receive the BNI with referral to aftercare AUD treatment and opportunity for a 2-week telephone booster delivered by a dedicated trained Health Promotion Advocate (HPA). The BNI contains the essential components of effective brief interventions [59–64] and includes training materials that have been used to train non-specialists in various clinical settings. First developed and evaluated in the YNHH Emergency Department (ED) [63–65], the purpose of the BNI is to assist patients in recognizing and changing levels of alcohol consumption that pose health risks. It relies on strategies from motivational interviewing (MI) and the stages of change model [66]. The main goals are to: (1) decrease ambivalence about reducing alcohol use; and (2) negotiate strategies for change. During the 15–20 min BNI, the HPA will: (1) *Raise the subject* of alcohol use; (2) *Provide feedback*: review the patient’s alcohol consumption, make a connection to the patient’s medical condition and reason for hospitalization; review guidelines for lower risk alcohol use; (3) *Enhance motivation*: via a readiness change ruler, which can assist in developing discrepancy; and (4) *Negotiate and Advise*: negotiate goal, provide feedback, ask patient to complete drinking agreement; summarize and arrange follow-up. Participants are provided a pamphlet regarding the impact of AUD on health and potential treatment resources (Additional files: 1, 2). The BNI session follows a structured encounter form and is digitally recorded for fidelity monitoring purposes. Consistent with our prior approaches [67, 68], the ED-based BNI manual was adapted by our team for relevance to racially and ethnically diverse hospitalized patients with AUD. All participants will be referred for formal AUD treatment per American Society of Addiction Medicine (ASAM) criteria [57] prior to discharge for ongoing AUD treatment. For this trial, we considered this the “control” condition given that this is consistent with standard of care [69] and we anticipate our approach guided by an interdisciplinary team with diverse perspectives and expertise will bolster this standard.

#### Facilitated provision of MAUD

For participants randomized to either BNI + MAUD or BNI + MAUD + CBT4CBT, the HPA provides education and counseling regarding MAUD as part of the BNI to the participant and communicates to the primary medical team that MAUD should be considered. Informed by existing resources and guidelines [70, 71], participants receive a pamphlet regarding MAUD (Additional files 1) and, via a note in the EMR, clinicians and staff are provided information regarding MAUD, including how to access the AUD treatment care signature pathway, with documentation of any stated preferences of the participant regarding a particular form of MAUD. Consistent with an effectiveness study, ultimate prescribing of any medication and the choice of medication is at the discretion of the primary medical team with the participant, and medications are provided via usual means.

#### CBT4CBT

For participants randomized to BNI + MAUD + CBT4CBT, the HPA gives the participant a username and password to access the web-based program on a provided tablet or their own device and encourages the participant to begin accessing the modules during their hospitalization (Additional files: 1, 2). Participants may continue accessing the program after discharge from the hospital. Available in English and in a culturally-adapted, Spanish version [35], this highly secure seven module program is modeled closely after the evidence-based NIAAA CBT treatment manuals [72]. Eight independent randomized trials have demonstrated the efficacy of CBT4CBT at improving substance use outcomes across diverse settings and populations [34, 35, 73–78].

#### Intervention training and monitoring

Training of HPAs includes didactics and practice exercises in both: 1) MI and 2) the BNI and its components. The MI portion of the curriculum includes 2 half-day training sessions: 3 h of instruction, beginning with an introduction to the fundamentals of MI (i.e., MI spirit and technical skills) and evidence of its effectiveness on reduction of alcohol use [79–82]; and 3 h of skills-based practice exercises using medical setting patient-specific cases. Similarly, the BNI section of the curriculum includes 2 half-day training sessions: 3 h of instruction, beginning with guidance on the steps of the BNI and support of its use among patients presenting in medical settings [64, 83, 84]; and 3 h of skills-based practice exercises. The following sections describe the critical components and training processes of MI and the BNI implemented in preparing the HPAs.

Prior to beginning study activities, HPAs were individually trained over 4 half-day workshops by a licensed psychologist with extensive experience in MI (Table 3). After this, the HPA demonstrated their acquired BNI skill with a final patient case. The patient case portion of the curriculum took 30 min. The case was specific to the study patient population and setting. The trainer was the patient actor and directly observed the implementation of the BNI. Feedback was provided at the time of the encounter. As indicated, to gain comfort and skills with working in the hospital setting and the outpatient AUD treatment referral network, the HPA additionally spent 2 days shadowing existing hospital-based HPAs who work in conjunction with YAMCS.

**Table 3:** Health promotion advocate motivational interviewing and brief negotiation interview training plan

Training agenda	Description of training
Motivational interviewing	
Half day 1	Introduction to the fundamentals of motivational interviewing: • Prochaska and DiClemente’s Transtheoretical Model^66^ • MI spirit (i.e., partnership, acceptance, compassion, evocation) • MI technical skills (i.e., open questions, affirmations, reflective listening, summaries [OARS] [132]) • Strengthening of change talk (i.e., self-statements that support change) and skillful handling and reduction of sustain talk (i.e., self-statements that support the status quo)^90^
Half day 2	OARS skills building using various practice exercises: • Development of open questions that pull for change talk, construction of reflective statements, and identification of strengths in patient statements Practice implementation: • Role-play demonstrations and simulated patient practice exercises with performance-based feedback
Brief negotiation interview	
Half day 3	Introduction of the BNI 4-step interviewing technique: • Didactic portion included instruction on (1) how to raise the subject of alcohol, (2) provide feedback on the patient’s drinking levels, (3) enhance motivation to reduce or stop drinking, and (4) negotiate and advise a plan of action [133] • Instruction of the implementation of the BNI from a SDOH lens and structural racism context • Watch video clips on the implementation of the BNI with patients in the emergency department to learn each step [134] • Watch recordings of the *Project ENHANCE* conference lecture series
Half day 4	Practice the BNI steps with the trainer: • Role-play demonstrations with patient facing pamphlets and documentation of patient treatment agreement • Live performance-based feedback was provided by the trainer

Adapted from the ED-based BNI manual [53, 65], the HPAs are provided with the *Project ENHANCE* intervention manual, structured encounter forms, and patient-facing pamphlets. The BNI session is audio recorded and reviewed periodically by a clinical psychologist to ensure fidelity to the intervention using a checklist (Additional file: 2) and feedback is provided at least weekly. Further, the HPAs are provided the opportunity to participate in a monthly teleconference with study investigators to reflect on experiences with delivering the intervention and intervention fidelity, with particular attention to considerations on how best to address SDOH and structural racism in the context of the BNI.

Costs of training and subsequent validation via demonstration included trainer and HPA time and materials. These costs were divided among the number of participants receiving interventions, plus the additional number of participants who could have received interventions from trainees over the subsequent year(s).

### Statistical considerations

#### Justification of sample size

Power for the three-arm trial was estimated based on an alpha of 0.025, which is a Bonferroni correction for multiple comparisons, given three treatment conditions with two planned contrasts. We assumed a 40% base rate in engagement in AUD treatment at 34-days post-discharge (primary outcome) following BNI based on our YAMCS post-discharge data and published reports from other hospital-based addiction medicine consult services [13]. A sample size of 450 (150 per condition) will provide adequate power (> 80%) to detect a 15% increase in engagement from the comparator treatment (BNI + MAUD or BNI + MAUD + CBT4CBT) at 34-days, consistent with a small to medium effect (delta = 0.34). With a sample size of 360 participants (120 per condition), we would have > 80% power to detect a 20% increase in engagement, consistent with a medium effect (delta = 0.46). Thus, with a randomized sample of 450, we will have adequate power to detect a medium effect on AUD treatment engagement, assuming a 20% loss of data at follow-up. This is based on experiences with similar populations at YNHH [65], as well as those in our prior outpatient treatment studies [34, 74, 76].

#### Statistical analyses

##### Primary and secondary outcomes

The primary objective of this analysis will be to determine if the proportion of participants with formal AUD treatment engagement (yes/no) at 34 days post-discharge differs among those randomized to BNI alone, BNI + MAUD, and BNI + MAUD + CBT4CBT in this diverse sample. The primary outcome (1a) of AUD treatment engagement is binary and we will use logistic regression with two planned contrasts to evaluate statistical significance of the differences in proportion of formal AUD treatment engagement (as verified through electronic medical record and/or contact with treatment facility). This statistical model will also be used to assess efficacy of study interventions on AUD treatment engagement at 90-days following randomization (1a). Secondary outcomes (1b) will include change in the self-reported percentage of heavy drinking days (PHDD) by month from the 30-day period prior to hospitalization (baseline) to the 34- and 90-day post-discharge timepoint, which will be evaluated with random effects regression statistical models. These models have several advantages in follow-up data from clinical trials of individuals who use substances, as they are less vulnerable than traditional MANOVA approaches to missing data [85, 86]. An additional secondary outcome will include change in biomarker PEth, which as a continuous outcome will be evaluated with a similar statistical approach. Given limited data on factors impacting PEth levels among hospitalized patients [87] and building from our own experiences [88], we will also evaluate the correlation between baseline self-reported alcohol use (in the 30-day period prior to hospitalization) and PEth levels (e.g., liver disease, hemoglobin [38]), factors associated with PEth levels adjusting for baseline self-reported alcohol use, and factors impacting change in PEth from baseline to follow-up. Exploratory outcomes of healthcare utilization, including rates and costs of emergency department visits and hospital readmissions during the 34- and 90-day post-discharge period will be evaluated with mixed model ANOVAs. Analysis of treatment effects on primary, secondary, and exploratory outcomes will include the following contrasts: *H1:* BNI + MAUD > BNI; BNI + MAUD + CBT4CBT > BNI + MAUD or BNI.

##### Exploratory outcomes

The objective of these analyses are to explore differences in intervention effectiveness within and across racialized groups, ethnicity, and SDOH using the same outcomes as Aims 1. The standard approach to moderation using regression models that include a ‘treatment by race interaction’ variable has multiple pitfalls, including lack of consideration of within-group differences [89]. Thus, we will incorporate suggested alternatives less likely to lead to misleading findings [89–91]. These include examining the efficacy of a specific treatment within a racialized and/or ethnic minoritized group (e.g., is BNI + MAUD significantly more effective than BNI for Black individuals?) as well as evaluating the efficacy of a specific treatment across different racialized and/or ethnic groups (e.g., are there significant differences in AUD treatment engagement for BNI + MAUD between Black and White individuals?). These analyses will be conducted with similar statistical approaches as Aim 1 (i.e., logistic regression for engagement outcomes, random effects regression for change in alcohol use outcomes). Furthermore, it is important to consider why specific treatments might yield more favorable results within or between certain racialized or ethnic groups. Accordingly, and informed by the Socio-Ecological Model (Fig. 1), we will evaluate SDOH as key moderators of intervention effects on AUD treatment engagement and alcohol use post-discharge. In these models, logistic regression will be used to examine the effect of the interaction of multiple individual-level SDOH (for example employment, education, housing instability, food insecurity, transportation insecurity, medical mistrust, discrimination experiences) by treatment condition on AUD treatment engagement post-discharge. We will also examine individual-level SDOH as a latent variable in Structural Equation Models evaluating the impact as a moderator of treatment effectiveness on post-discharge engagement and alcohol use outcomes.

#### Implementation-focused process evaluation

Informed by work of others [21] and our own experiences [40, 92], we will conduct an implementation-focused process evaluation to gain an understanding of the necessary factors for building the infrastructure for this clinical trial, delivering the associated interventions in the hospital setting, and then potentially sustaining the interventions outside the context of a funded study. We will ground our evaluation in RE-AIM (Reach, Effectiveness, Adoption, Implementation, Maintenance) with particular attention to promoting health equity [93–95]. To achieve these goals, data sources complementing participant assessments will include screening and enrollment logs (e.g., reasons patients decline study participation); recordings from intervention sessions; minutes from study team meetings; time, transportation, and other resources that participants as well as the HPAs and other healthcare staff contribute to interventions; as well as patient satisfaction data collected as part of the follow-up assessments.

Intervention-level cost per engaged patient will be calculated, along with increments in this cost as successive interventions are added. We will conduct analyses to determine the type, amount, and monetary values (i.e., costs) of the major resources used to implement each intervention for each patient, including time of professionals and patients, medication, and other intervention materials and resources. Combining cost with effectiveness data for individual patients will make possible descriptive scattergrams and group-level graphs of effectiveness = *f* [costs] and benefits = *f*[costs] as well as statistical analyses comparing effectiveness/cost ratios (e.g., change in PHDD per $100 of resources consumed by intervention) and other indices of relationships between costs and effectiveness for patients in each intervention condition. Average and median effectiveness/cost will be compared statistically for each condition with individual-level effectiveness and costs [58]. Cost per effectively treated patient also will be calculated at the group level, defining effective treatment according to quantitative definitions of clinically meaningful change in alcohol use. Similar analyses will examine graphically and statistically cost–benefit relationships as expressed by individual-level net benefit (i.e., reduction following intervention in costs of ED and readmission minus costs of intervention) [58]. Further, building on a mixed-methods evaluation that informed the study protocol [44], we will conduct a survey of hospital-based clinicians and staff upon conclusion of the clinical trial regarding their perspectives on the alcohol treatment interventions and necessary supports to sustain delivery of these interventions outside of the research infrastructure. We will also capture factors that may contribute to inequitable care among historically minoritized racial and ethnic groups.

#### Ethical approval and protection of participants

This HIPAA-complaint study is approved by the Yale School of Medicine Human Investigation Committee (protocol #2000031874). The Data Safety and Monitoring Board will review enrollment, baseline characteristics of enrolled participants, intervention delivery, adverse events, and outcome ascertainment biannually starting approximately 6 months after enrollment initiation.

#### Current status of *Project ENHANCE*

In preparation for study launch, the *Project ENHANCE* team engaged a variety of hospital and university-based stakeholders to refine study processes and prepare for implementation. This led to the development of a multi-pronged approach to inform recruitment strategies. In addition, based on these discussions, the *Project ENHANCE* team offered a set of four 1-h conferences to hospitalist-based clinicians regarding the importance of providing alcohol treatment in the hospital setting, treatment options, unique factors impacting minoritized racialized and ethnic populations, and the study protocol with an emphasis on CBT4CBT. Continuing medical education credits (CME) were offered for attendance with the goal of providing basic education regarding the importance of addressing AUD in the hospital setting; considerations for providing care through an equity lens; considerations for prescribing MAUD among medically complex patients; and the evidence for CBT4CBT.

After completion of this series and trainings of study staff, *Project ENHANCE* opened for recruitment on September 13, 2022, and recruitment and follow-up are ongoing.

## Discussion

*Project ENHANCE* will generate novel data on the comparative effectiveness of three different treatment strategies on promoting AUD treatment engagement and alcohol reduction post-hospital discharge among a diverse sample of patients with untreated AUD, with special emphasis on minoritized racial and ethnic patients. Our protocol is timely and highly relevant for several reasons. First, few prior studies have focused on the hospital setting to reach individuals with untreated AUD, though one important study focused on evaluating different formulations on naltrexone [14]. We build on this work by evaluating an intervention that is flexible with regards to the specific type and formulation of MAUD with ultimate prescribing at the discretion of the participant and primary team. Second, although there have been studies using digital interventions to address alcohol use in outpatient and emergency settings [96–98], to our knowledge, no studies have sought to initiate a robust digital treatment intervention to address AUD or any other substance use disorder in the inpatient hospital setting. Our trial will be amongst the first to evaluate the impact of the evidence-based CBT4CBT platform on AUD treatment engagement when initiated in the hospital setting [12, 26]. Third, our team’s diverse perspectives and expertise is unified by a commitment to promote health equity and address the role of structural racism as a driver of AUD and its consequences, as well as a barrier to treatment. We expect to generate new insights on the prevalence and correlates of SDOH among our sample of enrolled participants and the moderating effect of SDOH on intervention effects and outcomes, expanding beyond traditional measures of SDOH (e.g., housing status) [5]. Fourth, there is significant interest in complementing self-reported alcohol use with an alcohol biomarker, such as PEth [38], yet there have been few studies that collected PEth in the hospital setting. We anticipate examining the correlation between self-reported alcohol use and the biomarker PEth among patients experiencing acute illness and across clinically relevant subgroups [99] and its association with clinical measures over time (e.g., cognitive function). Lastly, given the hybrid type 1 effectiveness-implementation design, we hope to generate critical data including cost, cost-effectiveness, and cost–benefit, useful for informing future implementation of these different HPA-delivered treatment strategies in other large, urban health systems in diverse communities.

### Limitations

We expect limitations to our study. First, our focus has been to address the role of race and ethnicity in all aspects of study design and implementation; however, we have not had a similar focus on the role of gender that should be considered in future work. Second, while the MAUD conditions intend to facilitate MAUD through provision of patient and clinician facing materials, this may not be sufficient to stimulate actual prescribing. Third, the ongoing challenges of the COVID-19 pandemic have had a significant impact on clinicians, including burnout; clinicians may be less motivated, prepared, and able to address AUD in these circumstances. Fourth, Addiction Medicine Consult Services are increasingly available in hospitals across the United States, yet this still represents the minority of hospitals. In addition, the study is being conducted in an academic hospital system in New Haven, CT, which offers a range of accessible services for AUD upon hospital discharge. Thus, findings may not be generalizable to contexts where AUD treatment services are not as robust, including community hospitals and other regions of the United States. Fifth, all participant-facing documents and the CBT4CBT platform are available in English and Spanish and our team’s Research Coordinators are all fluent in English and Spanish. However, consistent with common practice at YNHH, interventions delivered by the HPAs will require use of translation services for Spanish-only speaking patients due to the skills of our current team members. Lastly, due to resource constraints, research coordinators and the HPAs will not be blinded to study conditions; no team member, however, will have a priori knowledge about group assignment prior to randomization.

## Conclusion

*Project ENHANCE* is expected to generate timely data to inform hospital-based practices on equitable strategies for promoting AUD treatment engagement and alcohol reduction among patients with untreated AUD to mitigate AUD-related morbidity and mortality.

## Supplementary Information

**Additional file 1:** Project ENHANCE Health Promotion Adovcate Intervention Manual.

## Data Availability

Datasets generated during the current study will be made available from the authors upon request and be made publicly available in a deidentified format consistent with NIAAA policies to the National Institute of Mental Health Data Archive repository.
