# Correction: Promoting alcohol treatment engagement post-hospitalization with brief intervention, medications and CBT4CBT: protocol for a randomized clinical trial in a diverse patient population

**DOI:** 10.1186/s13722-025-00558-x

**Published:** 2025-03-24

**Authors:** E. Jennifer Edelman, Oscar F. Rojas-Perez, Charla Nich, Joanne Corvino, Tami Frankforter, Derrick Gordon, Ayana Jordan, Manuel Paris, Jr, Melissa B. Weimer, Brian T. Yates, Emily C. Williams, Brian D. Kiluk

**Affiliations:** 1 Department of Internal Medicine, Yale School of Medicine, 367 Cedar Street, ES Harkness Memorial Hall, Suite 401, New Haven, CT 06510 USA; 2 Yale Program in Addiction Medicine, Yale School of Medicine, New Haven, CT USA; 3 Department of Social and Behavioral Sciences, Yale School of Medicine, New Haven, CT USA; 4 Department of Psychiatry, Yale School of Medicine, New Haven, CT USA; 5 The Consultation Center, New Haven, CT USA; 6 Department of Psychiatry, NYU Langone Health, New York, NY USA; 7 Hiorgdivisionic Clinic, Connecticut Mental Health Center, New Haven, CT USA; 8 Department of Chronic Disease Epidemiology, Yale School of Public Health, New Haven, CT USA; 9 Department of Psychology, American University, Washington, DC USA; 10 Department of Health Systems and Population Health, University of Washington School of Public Health, Seattle, WA USA; 11 Health Services Research and Development Seattle-Denver Center of Innovation for Veteran-Centered and Value-Driven Care, Veterans Health Administration (VA), Seattle, WA USA

**Correction:** ***Addict Sci Clin Pract*** **18, 55 (2023)**

10.1186/s13722-023-00407-9

Following publication of the original article [1], the authors discovered errors in retrieval of AUDIT-C data from the electronic medical record that led to the under-reporting admissions to a medicine service at Yale New Haven Hospital (YNHH), and thus would like to correct the text in **Study context** section.

In the **Study context** section, sentences are corrected from:

“For example, from July 1, 2022, to June 30, 2023, 1172 unique patients were admitted to a Medicine service at YNHH who had evidence of an alcohol-related diagnosis (based on clinical orders for Clinical Institute Withdrawal Assessment—Alcohol; AUDIT-C ≥ 7; and/or alcohol-related diagnosis). Among these patients, the mean age was 54 yo (standard deviation = 14.25) and the majority were men (67%). 15% identified as Hispanic or Latino and 27% as Black.”

To:

For example, from July 1, 2022, to June 30, 2023, 2209 unique patients were admitted to a Medicine service at YNHH who had evidence of an alcohol-related diagnosis (based on clinical orders for Clinical Institute Withdrawal Assessment—Alcohol; AUDIT-C ≥ 7; and/or alcohol-related diagnosis). Among these patients, the mean age was 54 yo (standard deviation = 14.8) and the majority were men (68%). 13% identified as Hispanic or Latino and 27% as Black.

The authors apologise for any inconvenience this may have caused.

The original article [1] has been updated.
