# Supplementary material for: Promoting alcohol treatment engagement post-hospitalization with brief intervention, medications and CBT4CBT: protocol for a randomized clinical trial in a diverse patient population
Source: Addict Sci Clin Pract. 2023 Sep 19;18:55. doi: 10.1186/s13722-023-00407-9 (PMC10510167; doi:10.1186/s13722-023-00407-9)
Supplement: Supplementary file 1 — Additional file 1: Project ENHANCE Health Promotion Adovcate Intervention Manual. [file 13722_2023_407_MOESM1_ESM.pdf]

Project ENHANCE

ENhancing **H**ospital-initiated **A**lcohol Treatme**N**t to In**C**rease **E**ngagement

Health Promotion Advocate Manual

Promoting alcohol treatment engagement post-hospitalization with brief intervention, medications, and CBT4CBT: A randomized clinical trial in a diverse patient population

Principal Investigators: E. Jennifer Edelman, MD, MHS and Brian Kiluk, PhD

Adaptations by:
Oscar F. Rojas Perez, PhD
Melissa B. Weimer, DO, MCR
Karen Ablondi, MPH, LCSW, LADC
Derrick Gordon, PhD
Ayana Jordon, MD, PhD
David J. Pagano
Emily C. Williams, PhD
E. Jennifer Edelman, MD, MHS*
Brian Kiluk, PhD*

**Contributed equally*

Text in this manual is excerpted and adapted from the Screening, Brief Intervention and Referral to Treatment (SBIRT) Training Manual For Alcohol and Other Drug Problems and from PROJECT ED HEALTH II BNI Training Manual

Created by:
Gail D'Onofrio, MD, MS; Michael V. Pantalon, PhD; Linda C. Degutis, DrPH; David A. Fiellin, MD; and Patrick G. O'Connor, MD, Department of Emergency Medicine, Department of Medicine, Section of General Internal Medicine; Yale University School of Medicine, New Haven, CT

Graphic design provided by Mason/Studio 63, LLC

Supported by: NIH/NIAAA, Grant# R01AA029820
Yale HIC Protocol #2000031874

Yale

Table of Contents

I. Study Overview ... 3
II. Overview of the Brief Negotiation Interview (BNI) ... 7
III. The 4 Steps of the BNI ... 8
IV. BNI+ Facilitated Provision of MAUD ... 18
V. BNI+ Facilitated Provision of MAUD+ CBT4CBT ... 21
VI. Communication with Primary Medical Team ... 32
VII. Documentation in Epic ... 33
VIII. Brief Negotiation Interview Telephone Booster Session ... 36
IX. Additional Motivational Strategies ... 44
X. Common Problems ... 45
XI. Appendices
Appendix 1 – The Spectrum of Alcohol Use ... 46
Appendix 2 – The Readiness to Change Ruler ... 47
Appendix 3 – Pros and Cons for Alcohol Use & Treatment ... 48
Appendix 4 – Alcohol Use Agreement & Referral Agreement ... 50
Appendix 5 – Case Example of BNI Dialogue for Alcohol Use Reduction ... 51
Appendix 6 – Plan to Promote Health ... 53
Appendix 7 – BNI - MAUD - CBT4CBT Checklists ... 54
Appendix 8 – BNI Trifold – English & Spanish ... 83
Appendix 9 – MAUD Trifold – English & Spanish ... 85
Appendix 10 – CBT4CBT Trifold – English & Spanish ... 87
Appendix 11 – Clinician Educational Materials ... 89
Appendix 12 – SUP_Intervention Adherence Rating Form ... 93
References ... 95

I. Project ENHANCE Study Overview

Overview

Among hospitalized individuals with untreated alcohol use disorder (AUD), the goal of Project **EN**hancing **H**ospital-initiated **A**lcohol Treatme**N**t to In**C**rease **E**ngagement (**Project ENHANCE**) is to evaluate the impact of three different treatment strategies on promoting AUD treatment post-hospital discharge and reducing alcohol use by individuals who meet criteria for an alcohol use disorder. This manual is written for training the health promotion advocates (HPAs) to deliver the treatments and is designed for delivery to individuals who meet criteria for AUD that has not been treated in the past 30 days.

The problem

AUD is a major threat to health and well-being, particularly among Black, Latinx, Indigenous and other minoritized individuals. AUD is a major cause of morbidity and mortality in the United States; and while the prevalence of AUD is lower among most racial and ethnic minoritized populations, than among White individuals, racial and ethnic minoritized groups generally experience more severe health and social consequences from AUD than White individuals. Further, AUD often goes untreated, especially among racial and ethnic minoritized individuals.

Potential solution

To leverage acute medical hospitalization as an important opportunity to initiate AUD treatment for untreated individuals with an AUD. These patients are some of the most vulnerable due to inequities in the social determinants of health (SDOH).

The following strategies independently, and particularly in combination, may promote AUD treatment engagement and alcohol reduction in racial and ethnic minoritized patients currently receiving treatment in acute medical settings:

- Brief intervention via the Brief Negotiation Interview (BNI) with referral and telephone booster
- Medications for AUD (MAUD)
- Computer-based training for cognitive behavioral therapy (CBT4CBT)

Current research question

What is the best approach to promote AUD treatment engagement and alcohol reduction post-hospitalization among racial and ethnic minoritized hospitalized individuals who meet criteria for an AUD that is not currently treated?

Individuals for whom these interventions are intended:

Inclusion Criteria *(must meet all criteria)*	Exclusion Criteria *(excluded if meet any of the following)*
Hospitalized at participating site	Have been engaged in formal AUD treatment in the past 30 days (i.e., excluding mutual help groups, such as Alcoholics Anonymous; this is including use of MAUD in the last 30 days)
Meet Diagnostic and Statistical Manual (DSM-5) criteria for a moderate to severe AUD (regardless of reason for hospitalization)	Meet DSM-5 criteria for moderate to severe opioid use disorder that is not currently treated
≥1 heavy drinking day in the 30 days prior to hospitalization	Self-reported or urine testing confirming pregnancy, nursing, or trying to conceive
Are ≥18 years old	Life-threatening or unstable medical, surgical, or psychiatric condition that prohibits study participation
Willing to consider medication treatment for AUD (MAUD)	Inability to provide ≥1 form of contact information
Willing and able to be contacted for follow-up	Anticipate being unable to return for follow-up assessments for any reasons, such as travel, planned procedure
	Inability to understand English or Spanish
	Currently in jail, prison, or other overnight facility as required by court of law and/or is considered a prisoner under local law or is under current terms of civil commitment or guardianship
	Are legally blind

Purpose of this manual

The current manual is designed to provide the necessary skills to easily and effectively perform:

1) A brief intervention, involving a Brief Negotiation Interview (BNI, with facilitated referral to treatment for alcohol and telephone booster) alone (BNI only);
2) BNI+ Facilitated Provision of Medications for Alcohol Use Disorder (MAUD); and
3) BNI+ Facilitated Provision of MAUD+ Computer-Based Training for Cognitive Behavioral Therapy (CBT4CBT) on AUD treatment.

The following sections describe the critical components of conducting the BNI, BNI+ Facilitated Provision of MAUD, and BNI+ Facilitated Provision of MAUD+ CBT4CBT for promoting both reduction of alcohol use and engagement in AUD treatment after hospitalization. Additional motivational and troubleshooting strategies and other helpful resources (e.g., communicating with primary teams, documentation in Epic, pros and cons of alcohol use, withdrawal checklists, sample BNI dialogues) are provided. This manual focuses on individuals who meet criteria for an alcohol use disorder only and does not address lower levels of alcohol use (i.e., at-risk) or other substance use.

It is important for the health promotion advocate (HPA) to do their best to embody the Motivational Interviewing spirit, which is made up of the following four domains:

1) partnership,
2) acceptance,
3) compassion, and
4) evocation.

Partnership means working collaboratively with the patient to arrive at a decision; ultimately the patient must decide to implement the change. **Acceptance** means valuing and accepting the patient for who they are, having empathy, and **respecting the patient's autonomy. Compassion** emphasizes understanding and pursuing the best interest of the patient while supporting their goals and values. Finally, **evocation** encourages HPAs to elicit patient strengths and capabilities.

Key considerations when engaging racial and ethnic minoritized populations: HPA's should consider the negative ramifications of structural racism including, the effects on inequitable educational systems, oppressive immigration laws, land displacement, and legacy of enslavement and "Jim Crow" era laws that may be contributing to alcohol use or ambivalence to engage in treatment (e.g., AUD treatment, physical and mental health treatment). For example, an HPA can inquire about interpersonal experiences of social injustices (e.g., discrimination, racism, prejudicial treatment), barriers to care (e.g., lack of childcare, financial resources, lack of culturally and linguistically responsive providers, transportation concerns, everyday life demands, lack of insurance, mistrust, cultural beliefs), anti-immigration related stressors, cultural destruction stressors, and experiences of social injustices. Another example may include personally mediated racism, Black and Brown people being treated unfairly because of what racial groups they are assigned to or identify with. An example of personally mediated racism is police brutality – where Black and Brown people being treated unfairly are more likely to encounter violence compared to their White counterparts. This inquiry of barriers can provide the HPA with critical contextual information on the individual's unique hurdles that should be considered at all levels of the planned intervention. For example, an HPA working with a Latinx individual may want to pay special attention to specific social stressors that may include their unique experiences around acculturation, immigration, changes in family dynamics resulting from acculturation, and or changes in social support as a result of family separation (due to immigration context). Further, an HPA working with a Black individual may want to highly consider the impact structural racism has had on their lived experience which may include, limited occupational opportunities resulting from segregated education and stolen wealth, redlining (e.g., disproportionate alcohol advertising and sales in neighborhood that are historically Black due to discriminatory practices), and police presence. Another example, an HPA working with an Indigenous individual may want to pay special attention to specific inequitable stressors that may be the result of past genocide, attempts to eliminate cultural and spiritual practices, and land displacement. Finally, special attention should be given to cultural values held by the racial and ethnic minoritized individual. For example, when engaging a Latinx, Black, and Indigenous individual, one can assess adherence to the core cultural values, such as family and respect.

Prior to implementing this manual with patients, HPAs should have:

1. received training in motivational interviewing,
2. received training in implementation of the Brief Negotiation Interview (BNI),
3. be familiar with Medication for Alcohol Use Disorder (MAUD),
4. reviewed Computer-Based Training for Cognitive Behavioral Therapy (CBT4CBT) platform,
5. have demonstrated competency in delivering the interventions with supporting materials (e.g., participant facing trifolds) via role play,
6. received cultural competency training; and
7. be familiar with the ASAM criteria and community resources for referral and referral pathways.

II. Overview of the Brief Negotiation Interview (BNI)

The Brief Negotiation Interview (BNI) was first developed in 1994 by Drs. Edward Bernstein, Judith Bernstein, and Gail D'Onofrio in consultation with Dr. Stephen Rollnick for Project ASSERT in the ED.[1-3] It was later refined and tested for unhealthy alcohol use in the ED and HIV clinics as part of a stepped care intervention.[4-6]

The BNI is a short, 15 to 20 minute counseling session and incorporates brief feedback and advice with motivational enhancement techniques to assist the patient in changing alcohol-related behaviors. The BNI procedure is patient-centered, and the skills used are based in large part on the patient's motivation and readiness to change. The primary goal of the BNI procedure is the patient's agreement to reduce alcohol amounts or accept a referral to a formal specialized treatment center to decrease harm (medical problems or trauma). The HPA and patient come to this agreement through a process of negotiation described in the following section.

The following are the 4 steps of the BNI:

1) Raise the Subject

- Establish rapport (you may want to acknowledge their minoritized status here and affirm their experience based on their self-identification [i.e., hearing what is it like to be…])?
- Raise the subject of alcohol use
- Assess comfort

2) Provide Feedback

- Review patient's alcohol use and patterns
- Make connection between alcohol use and negative consequences in a variety of areas of life including medical, legal, family, and employment with particular emphasis on context and history for racial and ethnic minoritized populations
- Make connection between alcohol use and the hospitalization
- Discuss issues related to physical dependence, such as tolerance and withdrawal

3) Enhance Motivation

- Assess readiness to change
- Boost motivation

4) Negotiate and Advise

- Negotiate goal
- Give advice
- Summarize and complete referral/or alcohol reduction plan

Each step has critical components, specific objectives, actions and necessary preparations to be successful as detailed in the following sections.

III. The 4 Steps of the BNI

The following is a detailed description of the actions to be taken during each of the 4 steps of the BNI. In each step, specific directions are given. Because all patients in this study meet criteria for AUD, the goal for patients randomized to **BNI only** would be the patient's agreement to follow-up with a referral to treatment upon hospital discharge and/or a reduction of alcohol use as the patient considers treatment options. If the patient is randomized to **BNI+ Facilitated Provision of MAUD**, the goal continues to be referral to treatment or a reduction of alcohol use as the patient considers treatment options; in addition, the HPA will discuss MAUD (See Section IV. BNI+ Facilitated Provision of MAUD). For patients randomized to **BNI+ Facilitated Provision of MAUD+ CBT4CBT**, the HPA will additionally incorporate a discussion of MAUD and introduce the patient to CBT4CBT to learn skills for reducing alcohol use (See Section V. BNI+ Facilitated Provision of MAUD+ CBT4CBT).

STEP 1: Raise the Subject

Critical components:

1. Be respectful
2. Remember, the patient giving you permission to discuss their alcohol use is an important aspect of the intervention
3. Avoid arguing or being confrontational
4. Be mindful of the patient's possible physical discomfort

Preparation

- Collect relevant demographic and background information from the research coordinator and/or chart review before meeting with the patient. This should include basic demographic characteristics and relevant clinical information regarding current hospitalization.
- Know which group the study patient is randomized to: BNI only or BNI+ Facilitated Provision of MAUD or BNI Facilitated Provision of MAUD+ CBT4CBT.

Sample Suggested BNI Dialogue

HOW TO START CONVERSATION:

"May we spend a few minutes talking about your use of alcohol." <PAUSE>

If the patient response is *"YES,"* use the following statement.

"I want to talk about how it's affecting you and how we might be able to help."

Follow the statement with the next questions.

"Tell me how you are feeling right now? When is the last time you drank alcohol?"

If the patient response is *"NO"* use the following statement.

"When would be a great time for us to discuss your alcohol related concerns?"

Or

"We don't have to talk about your alcohol use, we can talk about other concerns that you may have."

Summary of STEP 1

This first step sets the climate for a successful BNI. Asking permission to discuss the subject of alcohol use formally lets the patient know that their wishes and perceptions are central to the treatment, which later enhances the patient's acceptance of a referral to treatment and engage in a plan to reduce their alcohol use. Further reflecting on patient acknowledged problems and discomfort related to their alcohol use motivates the patient to start considering compelling reasons for change. This must be done in a way that is not confrontational and any information shared should not be delivered in a harsh (i.e., non-motivational) manner.

STEP 2: Provide Feedback

Critical components:

1. Review current alcohol use and patterns and symptoms or tolerance or alcohol withdrawal
2. Review spectrum of alcohol use and diagnosis of alcohol use disorder
3. Discuss the role of alcohol use on difficult areas of life (financial, family, employment, health, legal)
4. Make the connection between alcohol use and reason for hospitalization
5. Discuss issues related to physical dependence such as withdrawal and need to continually use alcohol (if applicable)
6. Inform patient that a variety of treatments work effectively to reduce and stop alcohol use, and ask patients for their positive treatment experiences

Preparation:

- Trifold
- National Institute on Alcohol Abuse and Alcoholism (NIAAA) guidelines

Sample Suggested Dialogue

Review patient's drinking patterns

Reflect patient's last statement and follow with the next comment.

"From what I understand you are drinking (fill in amounts from screening)."
BUT DO NOT RE-SCREEN

Follow the statement above with one of the next questions.

"How concerned are you with your alcohol use, if at all?" **Or** "*What concerns has it caused you, if any?"* **Or** *"How concerned or worried is your family with your drinking?"* **Or** *"How concerned or worried are members from your community with your drinking?"* <PAUSE>

Make sure to reflect the patient's statement. For example, "*it sounds like there is some concern with your alcohol use."*

Express concern about these patterns

Reflect the patient's statement above and assess alcohol use and medical illness connection with the following question:

"What connections might you see between your use of alcohol and your medical condition?" **Or** *"What connections might you see between your race and/or ethnicity, your use of alcohol, and your medical condition?"*

Reflect the patient's statement and follow it up with the next question.

"Has there been a time in your life when you stopped drinking or cut back in the past?"
"If 'YES,' please tell me about how you did it?"

"If 'NO,' tell me what has been helpful in your life in overcoming obstacles? **Or** *"In the past, how have you managed a difficult situation."*

IF PATIENT STATES ANY ACCURATE & RELEVANT CONNECTION(S),
Reiterate them, acknowledge (affirm) the patient's good insight, and give additional relevant facts on medical risks related to alcohol use.

IF PATIENT DOES NOT STATE ANY ACCURATE & RELEVANT CONNECTIONS,
MAKE the connection based on general caution regarding alcohol use:

"From research we know that any alcohol use compromises decision making and health behaviors. In addition, we know that any alcohol use can be harmful to health. Finally, research suggests that racial and ethnic minoritized groups generally experience more severe health and social consequences from alcohol use than White individuals."

Ask about a connection

Reflect patient's comment above and follow with the next question if patient did not already acknowledge a connection between alcohol and this hospitalization.

"What (if any) connection is there between your alcohol use and this hospitalization?" <PAUSE>

See next steps below.

Reflect the patient's statement

IF PATIENT SEES ANY ACCURATE & RELEVANT CONNECTION, reiterate (reflect) what they have said.

Provide medical facts and information that there is a connection between the hospitalization and drinking.

Based on the information obtained above, follow with one of the options below.

SAMPLE: *"You're right,* [reiterate patient's connection] *... that's a good connection to make."*

IF PATIENT DOES NOT SEE AN ACCURATE CONNECTION, then make one using facts (if applicable).

This is a key component of respecting autonomy: ***Ask for permission to share: Is it okay if I share an observation? If yes, follow the sample on next page.***

SAMPLE: *"One of the things that we see as a connection is that your patterns of alcohol use may have contributed to your fall, physical discomfort, or whatever they're in the hospital for."* (if relevant)

Follow the statement with: *"What do you make of that?"*

IF THERE ACTUALLY IS NO CONNECTION between the hospitalization and alcohol use, ask about any potential connections between alcohol use and overall health issues, or ask about connections between alcohol use and interpersonal experiences of social injustices (e.g., discrimination, racism, or prejudicial treatment, barriers to care, racial and ethnic stressors, harmful policies, police brutality). For example, an HPA may ask, *"how do social injustices* (e.g., police brutality, anti-immigration law, land displacement) *influence your alcohol use?"* **Or** *"As a* [insert race or ethnicity, persons self-identified gender] *influence/contribute to your alcohol use"* **Or** *"As a* [insert race or ethnicity, persons self-identified gender], *how do racial and/or ethnic injustices influence/contribute to your alcohol use?"*

Inform patient of NIAAA guidelines by using the trifold or card

Hand patient trifold (BNI only)

Whether a connection between hospitalization and drinking has been made, the next step is to provide information regarding the spectrum of alcohol use and recommendations regarding safer drinking limits; introduce relevant content on the BNI trifold.

Ask for permission to share information on NIAAA guidelines: *"Is it okay if I review with you what is considered safer drinking limits for your age and gender?"*

If the patient's response is *"YES"* proceed with sharing information. If they say *"NO",* say – *"tell me what you usually drink."*

If the patient's response was *"YES"* and drinking norms have been shared ask the following question: *"What do you make of the information just shared."* **Or** *"Within your cultural or social network, what are considered drinking limits?"*

This is where we want to bring it all together and use a summary. For example, *"Based on what we discussed so far, there seems to be some concern with your drinking and there is an indirect connection between your alcohol use and current hospitalization* [may consider making the connection to race and ethnic injustices as well]. *Did I get that correct?"*

If the patient's response is *"YES,"* move on to the following statement.

"So, in order to help you with all of this, I would like to talk with you a bit further about how we might be able to help you reduce the concerns associated with your alcohol use. For example, I want to encourage you to consider that there are a wide variety of treatment options available and that I am prepared to go over them with you." (See and show treatment referral list.)

Ask for permission to share treatment options: *"Is it okay if I share some options with you?"*

If *"YES,"* move forward with discussion treatment options (reference trifold).

Explain different types of treatment options available:

Medically Supervised Withdrawal Management (traditionally known as "Detox")
Partial Hospitalization Program (PHP)
Intensive Outpatient Program (IOP)
AA (Alcoholics Anonymous) and Narcotics Anonymous (NA)

To explore more positive thoughts about treatment ask *"What are some reasons for going? Have you thought about going before?"* **Or** *"Why might it be important to seek treatment now?"* **Or** *"What were some of the reasons why?"* **Or** *"Why is it important to have this conversation now?"*

"Would you mind If I gave you some possible reasons?"

If patient says, *"YES,"* then reflect on the possible consequences of treatment.

If patient says, *"NO,"* then discuss possible benefits of treatment (e.g., reversing the negative effects of physical alcohol dependence discussed in Steps 1 & 2).

Offer summary reflection of patient's reasons for starting or considering treatment, ending with a reinforcement of the patient's autonomy… "*Ultimately, the decision to seek treatment is up to you."*

Close with: *"If you decide to accept our recommendation, you would be taking the first step towards addressing the concerns connected with your alcohol use that we've been discussing."*

Summary of STEP 2

This step provides the opportunity to offer education related to specific patient issues. There is opportunity at four different levels that can be used towards the next step of enhancing motivation, i.e.,

1) Linking the hospitalization and the patient's alcohol use to the problems that they acknowledge they are feeling in their life such as financial, family, employment, health, and legal problems and;
2) Connecting the hospitalization to the alcohol use if possible, such as withdrawal, injury, lack of follow-up, risk of injury, etc;
3) Discussing the cycle of tolerance, withdrawal, and ongoing alcohol use and;
4) Should they decide to accept the recommendation, treatment would help.

STEP 3: Enhance Motivation

Critical components:
1. Assess readiness to change
2. Boost motivation
3. Use open-ended questions
4. Use of reflective listening

Preparation:

- Readiness to Change Ruler (Appendix 2 on page 47)
- Pros/Cons of Treatment (Appendix 3 on page 48)

Sample Suggested Dialogue

Ask patient to identify readiness to change on Readiness Ruler show card

Start with: *"Now that we have discussed treatment options, on a scale from 1-10, where 1 is 'not at all' and 10 is 'right now', how ready are you to engage in alcohol treatment after your hospitalization?"*

Ask why they choose that number and not a lower one

If patient says:
- ≥ 2, ask *"Why did you choose that number and not a lower one?"* (i.e., *"What are some reasons you would engage in treatment?" "What else?"*

This is intended to pull for change talk, a patient's own argument for change. The change talk is then reflected. See below.

If the patient's response is 1 or unwilling, ask *"What would it take for that "1" to turn into a "2"?* **Or** *"Imagine you did go to treatment; how could that be helpful to you?"*

Or

"What would have to happen for you to be ready? "How important would it be for you to prevent that from happening?"

Reflect/reiterate positive reasons for change.

Discuss pros and cons of treatment.

Reflect patients' statements regarding change

Then REFLECT on their reasons and reinforce with clinical information *(e.g., "Treatment does in fact work to help reduce and eliminate use, as well as the problems connected with it.")*

Take the patient's answer from the above question and ask: *"What makes that reason important to you?"*

Summary of STEP 3

Patients with AUD spend much of their time justifying their use by rehearsing reasons to continue using. However, in Step 3 of the BNI, specific motivational enhancement techniques are utilized to reverse this so that the patient begins to clarify and, through HPA reflections, reinforce highly personal reasons in favor of either reducing alcohol intake to lower-risk levels or engaging in treatment for their alcohol use (vs. continued use).

The primary tools to promote such a discussion of reasons or motives for treatment engagement are:

1) The Readiness Ruler questions (1-10)
2) Asking why they did not select a lower number and other questions about positive reasons offered
3) Selectively reflecting on reasons that promote treatment-seeking. Exploring this gap between the patient's current situation and how their life might be if they reduced their intake or engaged in treatment, as well as stressing their autonomy and freedom to choose or not to choose these options, are the things that often tip the scale in terms of changing behavior.

STEP 4: Negotiate and Advise

Critical components:

1. BNI only – Negotiate a plan on how to cut back or engage in treatment and reduce harm
2. BNI+ Facilitated Provision of MAUD – Introduce MAUD, negotiate a plan on how to cut back or engage in treatment and/or reduce harm and speak to their hospital-based clinicians about MAUD (See Section IV)
3. BNI+ Facilitated Provision of MAUD+ CBT4CBT – Introduce MAUD, negotiate a plan on how to cut back or engage in treatment and/or reduce harm, speak to their hospital-based clinicians about MAUD, and learn how to complete the CBT4CBT modules. (See Section V)
4. Direct advice
5. Alcohol use agreement or referral agreement and provide informational trifold
6. MAUD and MAUD+ CBT4CBT informational trifold

BNI Alone - Preparation:

- Agreement forms (Appendix 4 on page 50)
- Referral (Appendix 4 on page 50)
- Trifold

Sample Suggested Dialogue

Elicit response (How does all this sound to you?)

Reiterate what participant says in above and say, *"What's the next step?"* [PAUSE] *"Are you accepting the referral?"*

Negotiate the goal (What would you like to do?)

IF *"YES"* to treatment, skip to bottom "Secure Referral".

IF *"NO,"* reiterate reasons participant gave above and <PAUSE>.

IF STILL *"NO,"* then ask if the patient might consider it and give advice below for patient to have, even if they do not want to consider it, and then SKIP TO "Provide Trifold." In this situation, an HPA may say the following, *"What would need to happen in order for you to engage/consider treatment for your alcohol use?"* Once the patient has responded, reflect statement and ask for permission to share additional reasons why engaging in treatment may be beneficial. If the patient response is *"NO,"* respect the patient's autonomy and move forward.

Give advice if necessary

Based on the response above, follow with: *"If you leave the hospital with a plan for continued treatment for your alcohol use, you will be on the road to better health* (See Appendix 4). *Based on*

what you told me and what we know about alcohol use disorder, I think you should...", "it may be a good idea to address this now..."

"Remember what we discussed in terms of improving your health."

Once a *"YES"* has been secured, follow with: *"Which of these options might you be interested in?"* [SHOW LIST]

Summarize (This is what I've heard you say...)

Have patient fill out agreement card

"Let's complete this referral agreement together which will reinforce your decision to seek treatment. This is really an agreement between you and yourself."

"Ok, based on your preferences, your insurance information and availability of programs, I can make a few phone calls to schedule an appointment for you. You may even be able to speak to someone while you are here in the hospital."

"Once I make a few calls, I will come back and we can update the referral agreement. How does that sound?"

Upon return, review information with the patient and update the referral agreement.

Provide summary
Update form with appointment date and time
Get signature
Ask if they have any questions: *"What questions do you have?"*

Thank the participant for their time

Summary of STEP 4 BNI Alone

In Step 4 of the BNI, the HPA assists the patient in exploring a menu of options regarding reducing their alcohol use and engaging in treatment. The HPA also attempts to negotiate a formal agreement around one of these goals (i.e., alcohol reduction or a referral to a formal alcohol use disorder treatment program), where the patient ultimately completes and signs an agreement form. If the patient is not ready to make an agreement, then additional advice is given, and options are again discussed in another attempt to negotiate and motivate the patient. However, the patient is the decision-maker and should ultimately be responsible for their plan.

Step 4 is ended by scheduling a Booster session (phone call) to occur 2-weeks after they discharge from the hospital and asking the patient if they have any questions about the information or instructions and by thanking them for their time.

IV. BNI+ Facilitated Provision of MAUD

For patients randomized to BNI+ Facilitated Provision of MAUD, the HPA will provide education and counseling regarding MAUD as part of the BNI to the patient and communicate to the primary medical team that MAUD is indicated and should be considered.

The following medications are FDA approved for AUD

1. **Naltrexone** which comes in a pill or injection,
2. **Acamprosate** which comes in a pill, and
3. **Disulfiram** which comes in a pill.

There are other medications such as gabapentin and topiramate that are sometimes used as well.

MAUD can help individuals with AUD to:

- Regain a stable state of mind, free from the highs and lows that can come from alcohol use
- Provide freedom from thinking about alcohol
- Reduce craving for alcohol
- Focus on healthy lifestyle

Taking MAUD is like taking medication to treat any other medical condition. It is not substituting one drug for another, but rather treats AUD. These medications are safe and can be taken for years, if needed.

Naltrexone (nal-trex'-one)

- Decreases or completely blocks the enjoyable effects of drinking
- Decreases return to any drinking and heavy drinking

Acamprosate (a-kam'-pro-sate)

- Repairs chemical imbalance in systems of the brain responsible for excitation
- Decreases return to any drinking

Disulfiram (dye-sul'-fi-ram)

- Helps people avoid alcohol by causing unpleasant effects when alcohol is consumed, including:
  - Flushing in the face
  - Throbbing headache
  - Difficulty breathing
  - Nausea
  - Vomiting/sweating

Disulfiram does not decrease alcohol cravings and is appropriate for individuals interested in achieving abstinence. Those taking it should avoid alcohol in all forms including mouthwash and over-the-counter medications.

Topiramate (toe-pyre'-a-mate)

- Decreases craving for alcohol
- Repairs chemical imbalance in systems of the brain responsible for excitation and reward

What are the side effects of MAUD?

It's important the HPA understands that there may be side effects of MAUD. Encourage the patient to discuss medication side effects and contraindications with their health care clinicians and reassure them that these medications are generally well tolerated.

Sample MAUD Suggested Dialogue to Incorporate in the BNI

BNI+ MAUD - Preparation:

- Trifold

Introduce MAUD

Once the BNI is complete, follow with the next statement.

"As part of the plan for addressing your alcohol use, I would like to talk to you about medications that can help reduce cravings. Is it okay if I discuss that with you?"

If they say *"YES,"* move on to the next statement.

If they say *"NO,"* reiterate reasons participant gave above about wanting to reduce alcohol use.

"Like medications for high blood pressure or diabetes, these medications can be prescribed by any doctor. Medications can be used in combination with other treatments like AA and can also be used without other treatments."

Introduce the trifold (MAUD)

"There are three main medications available to reduce alcohol use: naltrexone, acamprosate, and disulfiram. These medications come as a pill form to be taken once a day, like other medications, and one of them – naltrexone – also called Vivitrol – is available as a monthly injection. There are other medications such as gabapentin and topiramate that are sometimes used as well."

"Have you ever received any of these medications?" If "YES", which medications?"

"Some medications (except disulfiram [also known as Antabuse]) can be started or continued while drinking alcohol."

"These medications can be very helpful for decreasing cravings to drink alcohol and are very safe."

Reflect participants statement and move on to the next statement.

"Before I go, I would like you to summarize what we discussed... Finally, based on my understanding you are interested in finding out more about medications to help with alcohol use [may specify medication if any particular option favored] *and will discuss options further with your primary care team."*

Update the agreement form and thank the participant for their time

SUMMARY OF STEP 4 BNI + Facilitated Provision of MAUD

In Step 4 of the BNI+ Facilitated Provision of MAUD, the HPA assists the patient in exploring a menu of options regarding reducing their alcohol use and engaging in treatment in addition to exploring MAUD. The HPA also attempts to negotiate a formal agreement around one of these goals (i.e., alcohol reduction, a referral to a formal AUD treatment program, and/or MAUD), where the patient ultimately completes and signs an agreement form. If the patient is not ready to make an agreement, then additional advice is given, and options are discussed in another attempt to negotiate and motivate the patient. However, the patient is the decision-maker and should ultimately be responsible for their plan.

Step 4 is ended by scheduling a Booster session (phone call) to occur 2-weeks after discharge from the hospital and asking the patient if they have any questions about the information or instructions and by thanking them for their time.

V. BNI+ Facilitated Provision of MAUD+ CBT4CBT

For patients randomized to BNI+ Facilitated Provision of MAUD+ CBT4CBT, the HPA will provide education and counseling regarding MAUD as part of the BNI to the patient and communicate to the primary medical team that MAUD is indicated and should be considered. In addition, the HPA will encourage the patient to agree to participate in the CBT4CBT computer-based training modules.

Sample CBT4CBT Suggested Dialogue to Incorporate in the BNI

BNI+ MAUD+ CBT4CBT - Preparation:

- Trifold

Script for HPA to describe CBT4CBT

"As part of your participation in this research study, you have been assigned to receive a computerized version of cognitive behavioral therapy, called CBT4CBT. I'd like to spend a few minutes telling you about it."

Provide CBT4CBT Trifold

POINT TO SECTION OF TRIFOLD: "What is cognitive behavioral therapy?"

"The CBT4CBT program is designed to teach you skills and strategies to help you reduce or stop drinking. It is based on a type of talk therapy, called cognitive behavioral therapy, or CBT, which is one of the most effective treatments for those wanting to change their alcohol use. Rather than a counselor teaching you the skills in CBT, you will learn them on your own through this interactive computer program."

POINT TO SECTION OF TRIFOLD: "CBT4CBT will teach you how to..."

"The CBT4CBT program will help you learn how to recognize, avoid, and cope with your triggers and things that set you up to drink. There are 7 total lessons in the program, and each one takes about 35-40 minutes to complete. But you'll be able to go at your own pace and can stop and restart a lesson whenever you like. The program has a narrator who will guide you through the lessons. Each lesson includes some brief movies and interactive exercises to help you learn skills to address your alcohol use."

"The CBT4CBT program has been used in many prior studies and is effective at helping people reduce or stop their alcohol or drug use. It is completely safe and confidential. You will not be entering in any personal information."

Important information to convey

- The great thing about this program is that you can do it whenever and wherever you want. You don't have to make an appointment or talk to anyone.
- We recommend that you access the program while in a quiet and private space. Think of this like a therapy session - you want to make sure you can give it your full attention without distractions.
- You will need to hear the narrator and characters in the program, so try to find a quiet space if possible. Headphones could be helpful.
- There are a lot of videos in the program, so we recommend using a device with a large screen (10 inches or greater). A tablet or laptop is recommended for optimal viewing. You can use your phone to access the program, but you may need to scroll a bit to see everything on the screen.
- We ask that you complete at least one CBT4CBT lesson while you're here in the hospital. After you leave the hospital, we recommend you complete 1 or 2 lessons per week. But you are free to access it as often as you like.
- Ask the patient about their access to internet; if they do not have internet at home, discuss with them places they may access it for free (e.g., the library, coffee shops).

Script and Procedure for HPA to help patient log in to Computer-Based Training for Cognitive Behavioral Therapy (CBT4CBT)

"Before I show you how to access it, do you have any questions?"

Answer any questions the participant may have regarding the program.

"*Now, I'm going to show you how to access it and will help you log in the first time."*

POINT TO SECTION OF TRIFOLD: "How Do I Access It?"

"To access the program, you'll go to the following website – home.cbt4cbt.com"

"Or you can scan the QR code here on the trifold using your camera."

"Although the program can be accessed on a phone, the best way to view it is on a screen larger than 10 inches. So, we highly recommend using a tablet or laptop. But if you're more comfortable on your phone, that's OK too."

HELP PATIENT ENTER IN WEB ADDRESS ON THEIR DEVICE

SHOW THEM HOW TO SCAN QR CODE USING THEIR CAMERA

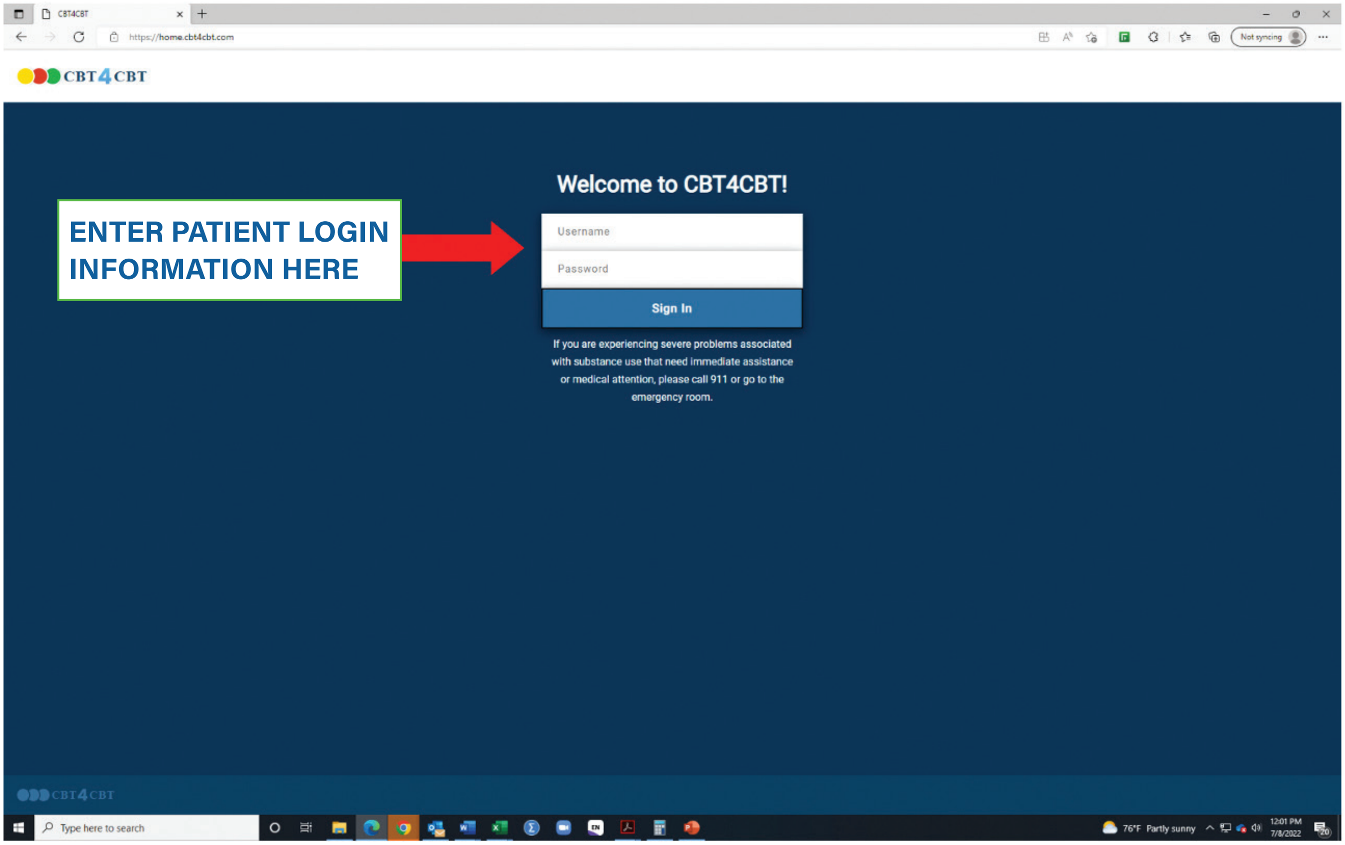

"Here is the main screen where you will log in to the program."

"Let's enter your login information."

"Please type in the following - Your username is…"

"Your password is…"

"You'll need this information each time you log in. Let's write these down so you can remember them. You might also want to keep it somewhere in your phone so you can access it if you forget."

HELP PATIENT WRITE DOWN USERNAME AND PASSWORD. SHOW THEM HOW TO PUT IT IN SMARTPHONE 'NOTE' FUNCTION IF NEEDED

"If you ever forget your log in information, you can call or text our study coordinator at xxx-xxx-xxxx."

PROVIDE STUDY COORDINATOR CONTACT INFO IF PATIENTPATIENT DOES NOT HAVE

"Once you enter the login information, click/touch SIGN IN."

HAVE PATIENT CLICK/TOUCH SIGN IN

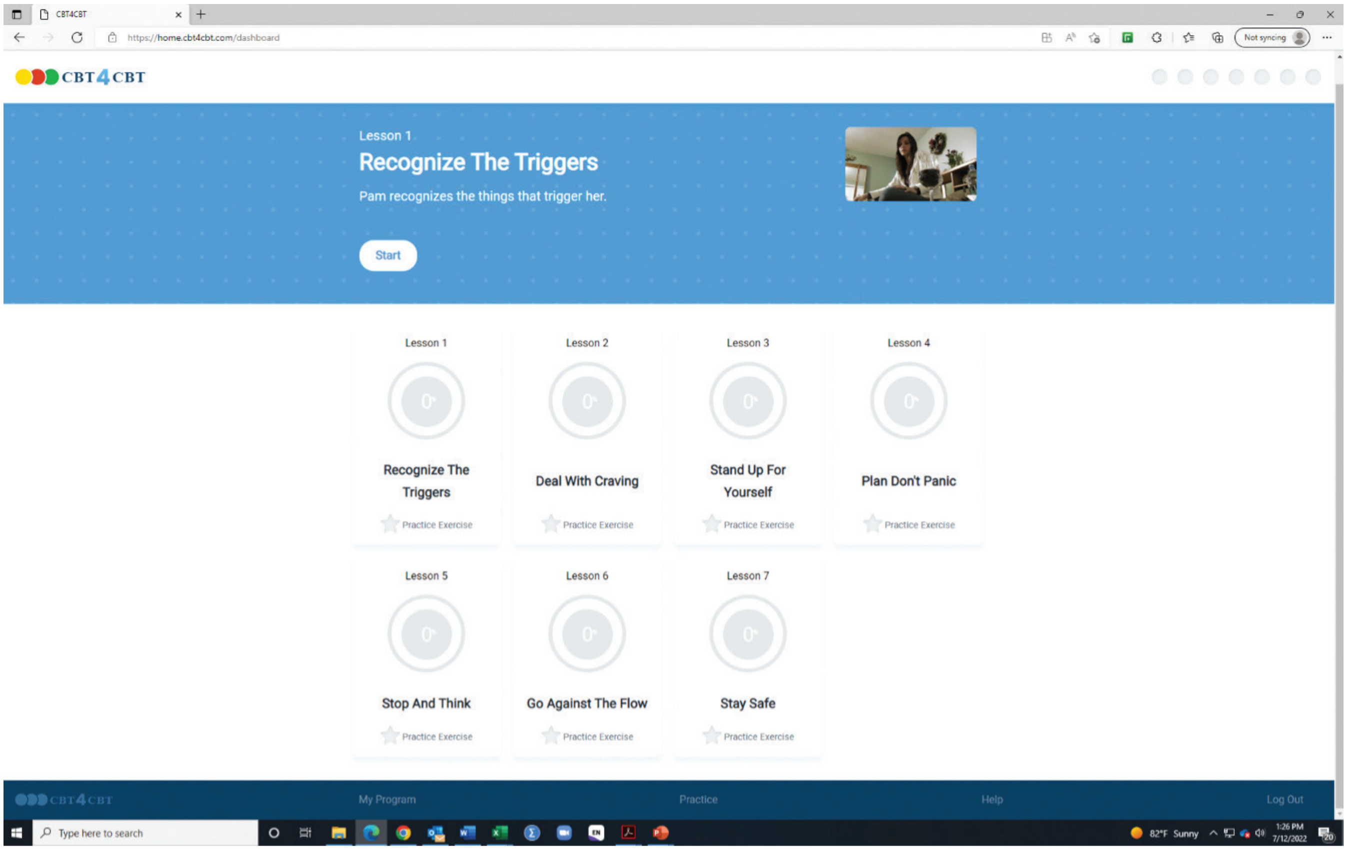

"This is the home page of the program."

"You'll see the 7 lessons here on the home page. The first lesson is highlighted at the top of the page – it's called Recognize the Triggers. Before you start the lesson, let me just show you around the home page."

"Here are the 7 lessons. They each have a grey circle that keeps track of your progress in each lesson. Right now, they're all at 0%."

POINT TO THE PROGRESS CIRCLE FOR EACH LESSON

"When you complete a lesson, the circle will fill up and you'll receive a badge for completion. If you leave a lesson before completing it, the circle will show you what percentage you've completed."

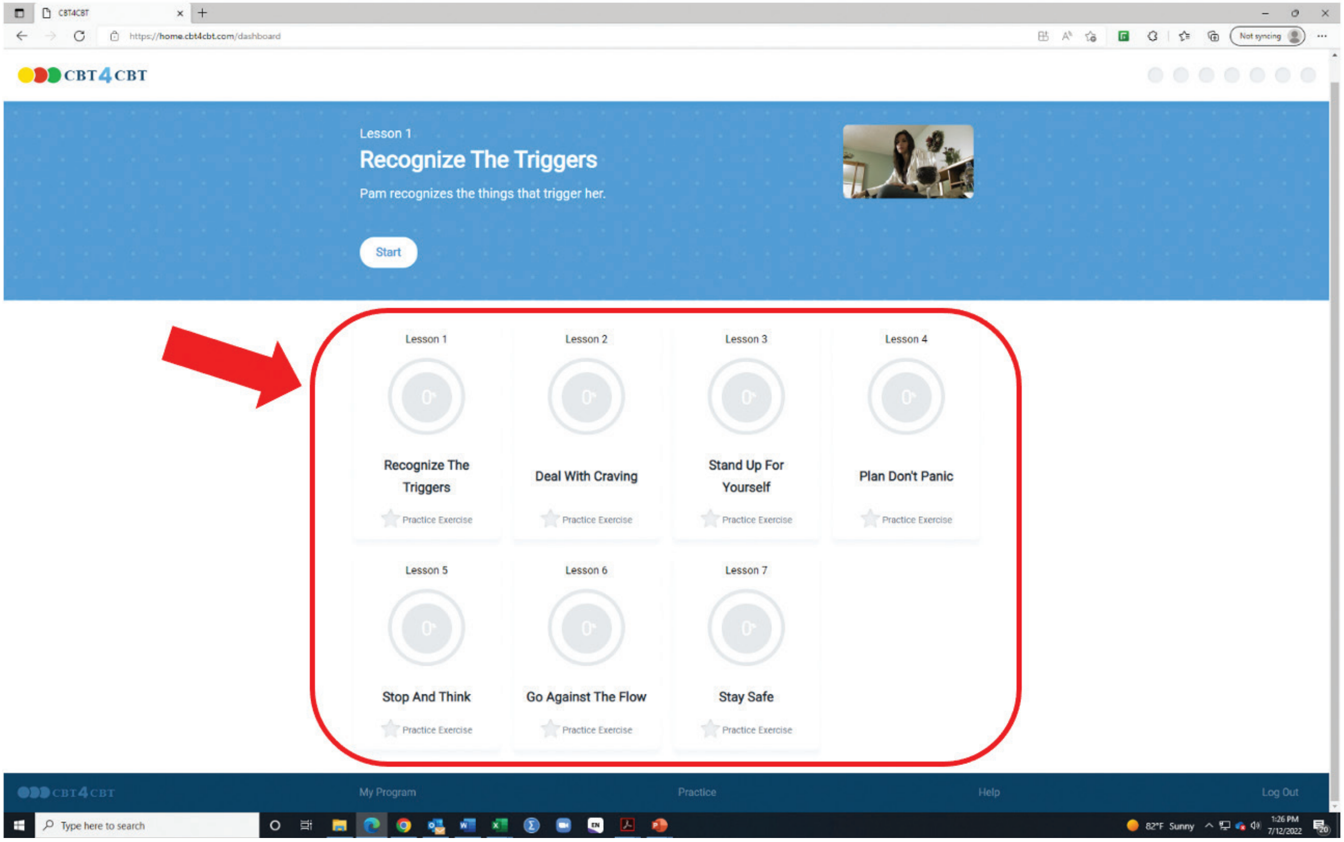

"Under the name of each lesson, there's a small star with the words PRACTICE EXERCISE."

POINT TO THE PRACTICE EXERCISE STAR

"This star will light up once you complete a practice exercise for that lesson. The practice exercises are a very important part of the program. The more you practice these skills, the better you'll do."

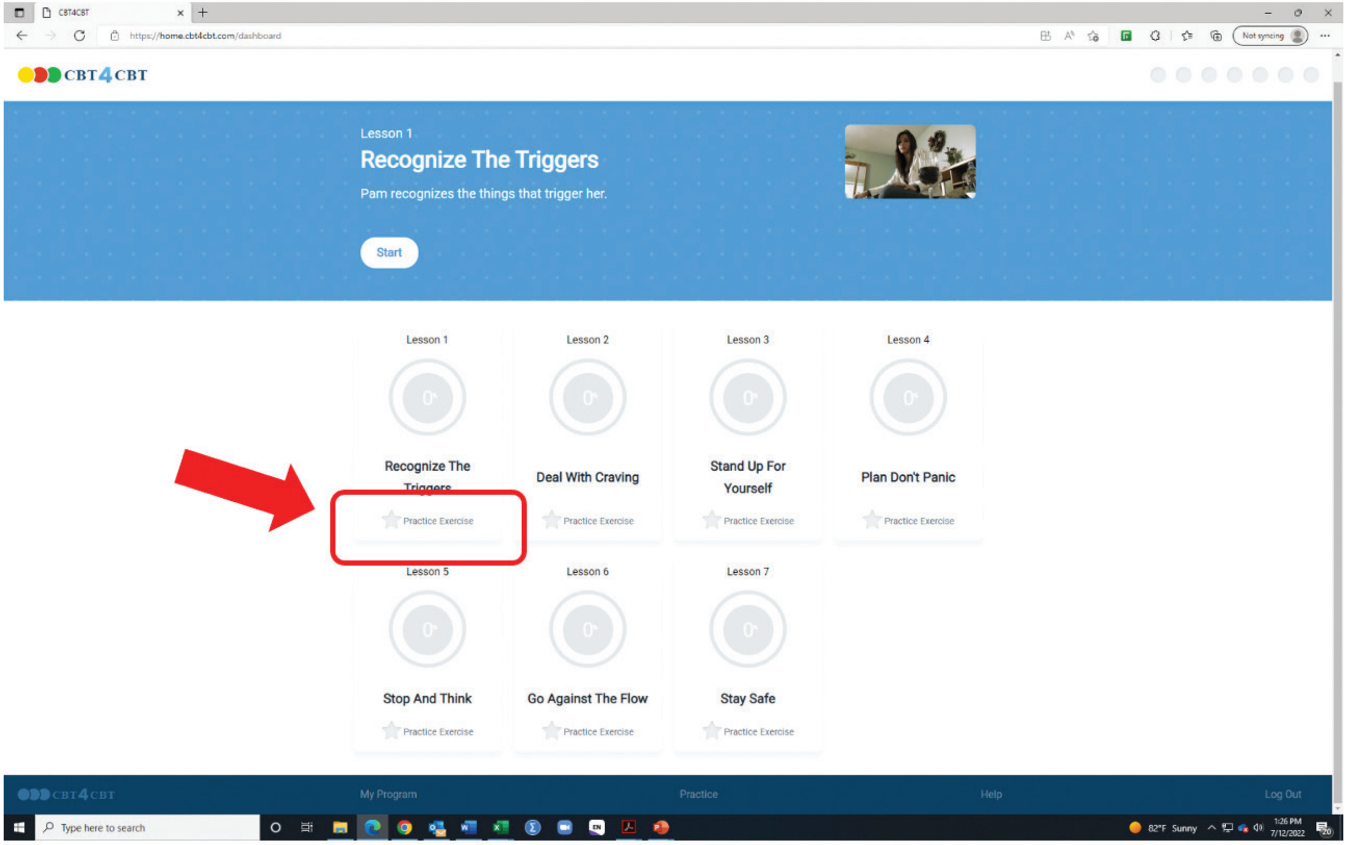

"Along the bottom of the page, there are 4 different tabs that will take you to different areas of the program."

POINT TO THE TABS AT THE BOTTOM OF THE PAGE

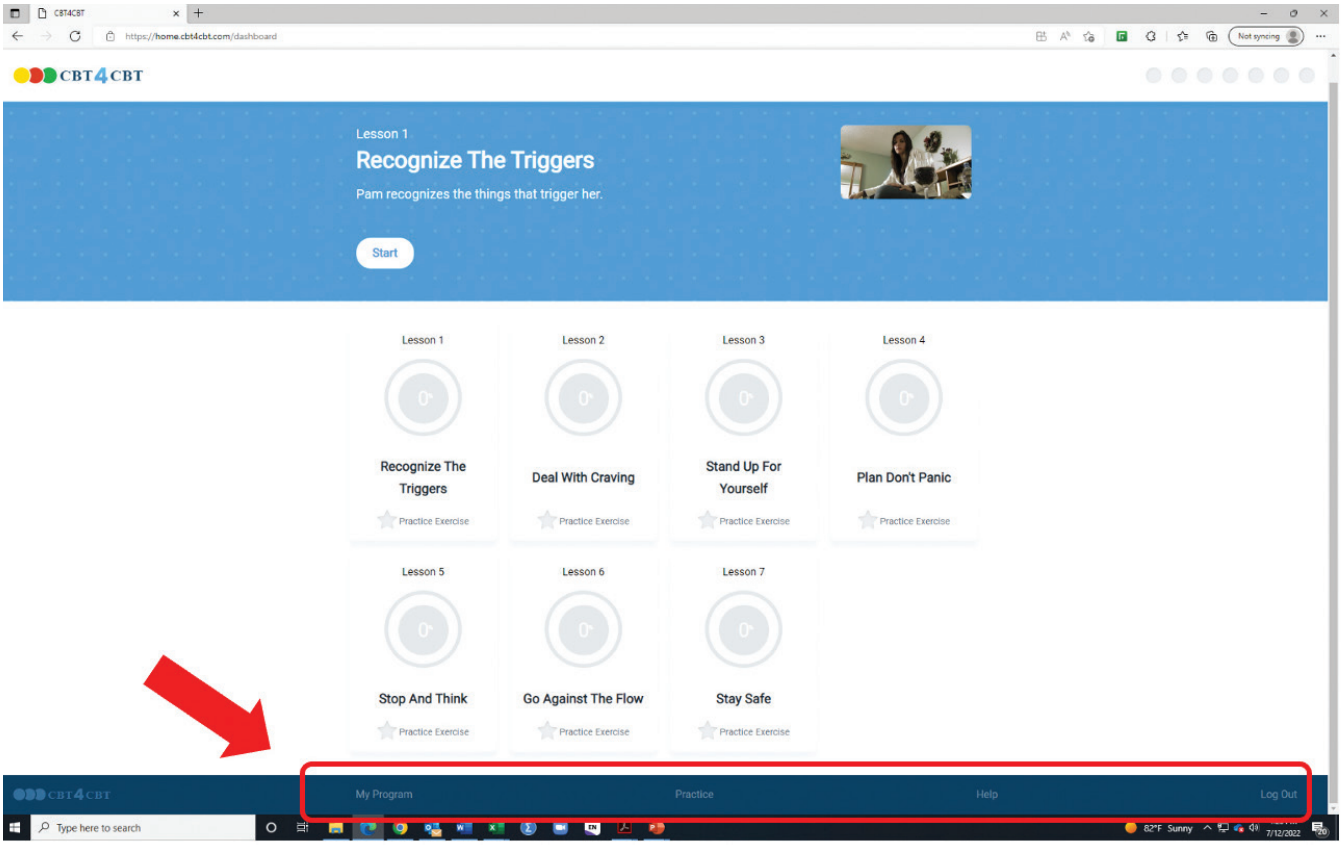

"The tab called MY PROGRAM will take you to this home page with all the lessons. When you are in one of the lessons and want to get back to this home page, just click/touch the MY PROGRAM tab at the bottom."

"The tab called PRACTICE will take you to a page that has all of the practice activities within each lesson."

HAVE PATIENT CLICK/TOUCH PRACTICE TAB TO SHOW THEM WHERE IT LEADS

"This is the Practice page. These are all blank right now because you haven't done any practice activities yet. In each lesson, you'll be asked to complete practice activities on the screen. You'll be able to download blank practice worksheets here on this page whenever you want to practice a skill."

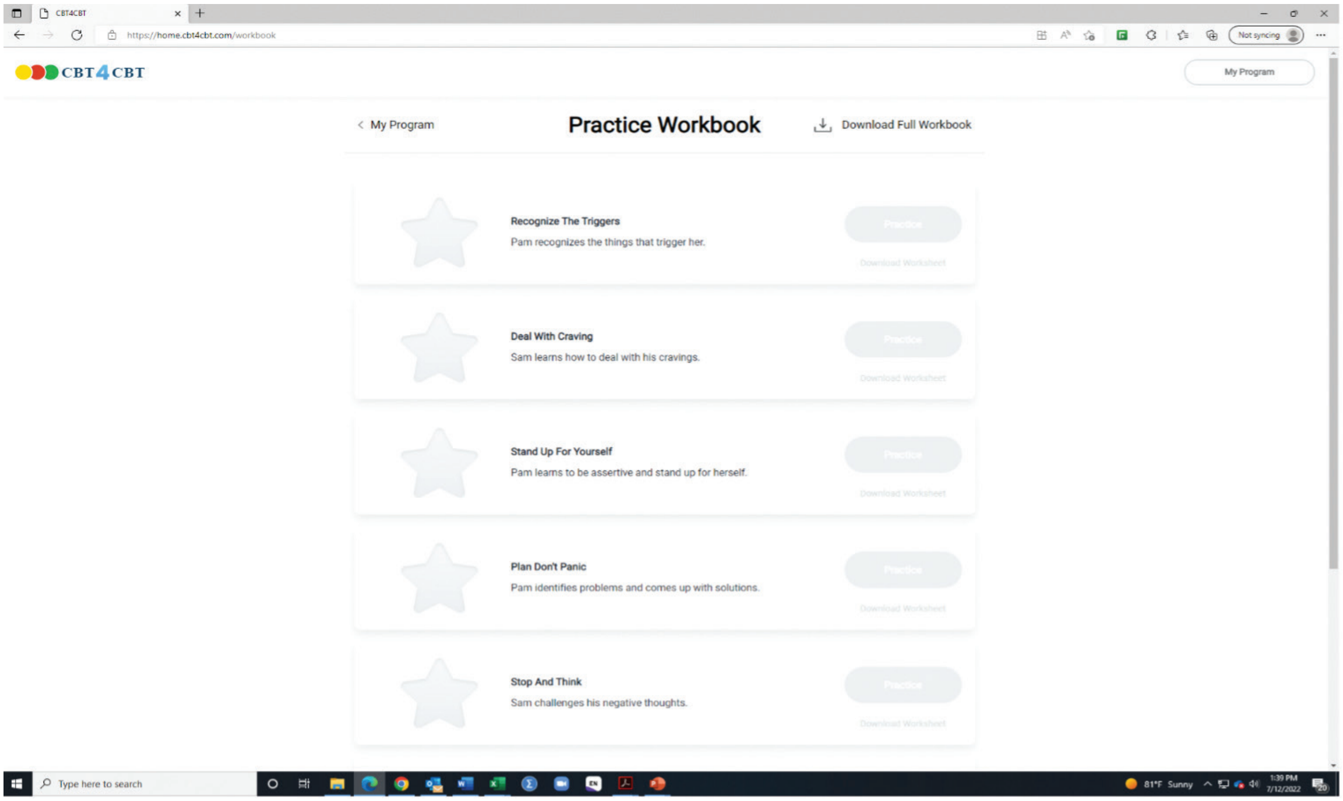

"Please scroll down on the PRACTICE page to see the tabs at the bottom of the page. Touch/click the MY PROGRAM button to go back to the home page."

HAVE PATIENT CLICK/TOUCH MY PROGRAM TAB

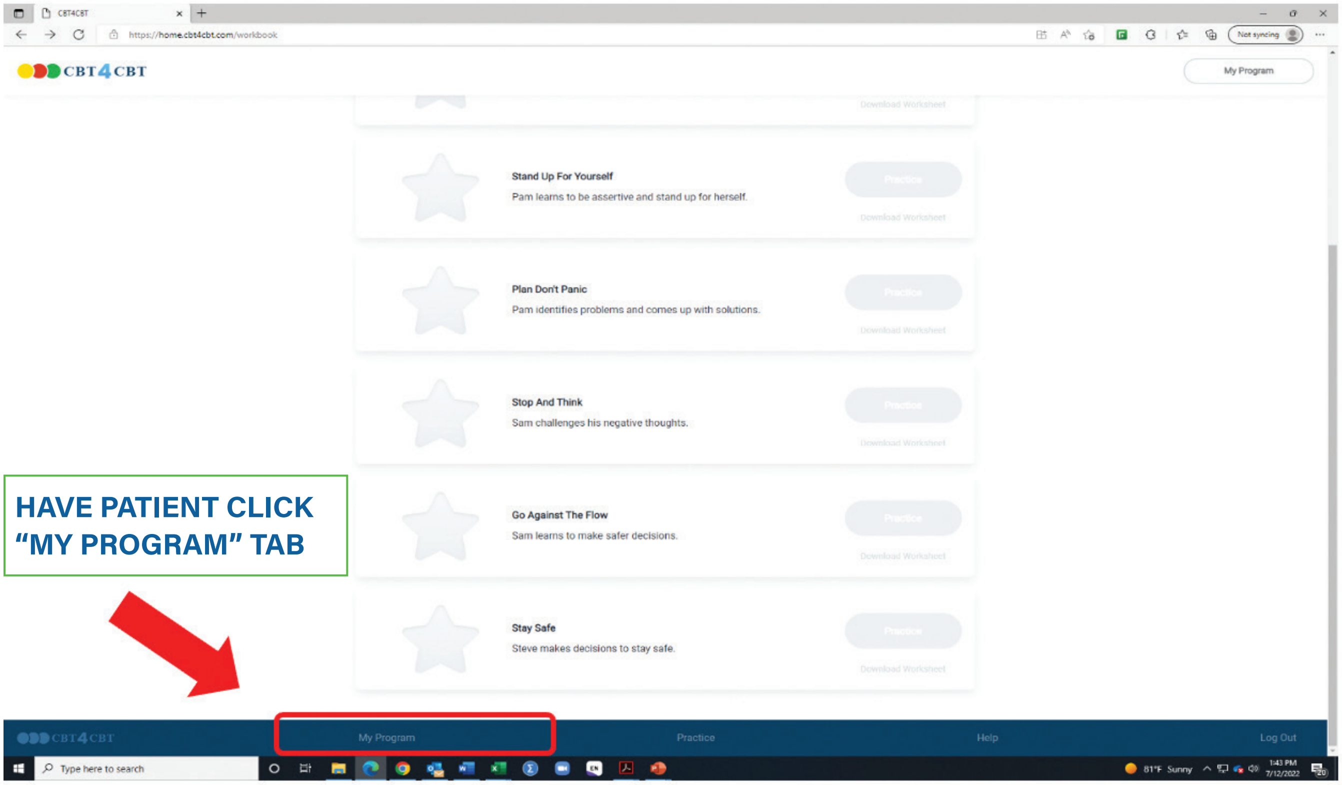

"Now we're back on the home page with all the lessons. The LOG OUT tab at the bottom of the screen will log you out of the program. Whenever you click/touch that tab, you'll be logged out and will go back to the login screen to enter your information again."

"The HELP tab on the bottom will bring you to a brief video to test the sound volume. Otherwise if you're having trouble with the program, you'll need to contact the research coordinator at xxx-xxx-xxxx."

"OK, when you're ready to start the lesson, just click/touch the start button at the top. This will take you to the first lesson, where a narrator will appear and will guide you through the lesson. Again, at any point, if you want to come back to this home page, just click/touch the MY PROGRAM tab at the bottom."

HAVE PATIENT CLICK/TOUCH THE START BUTTON

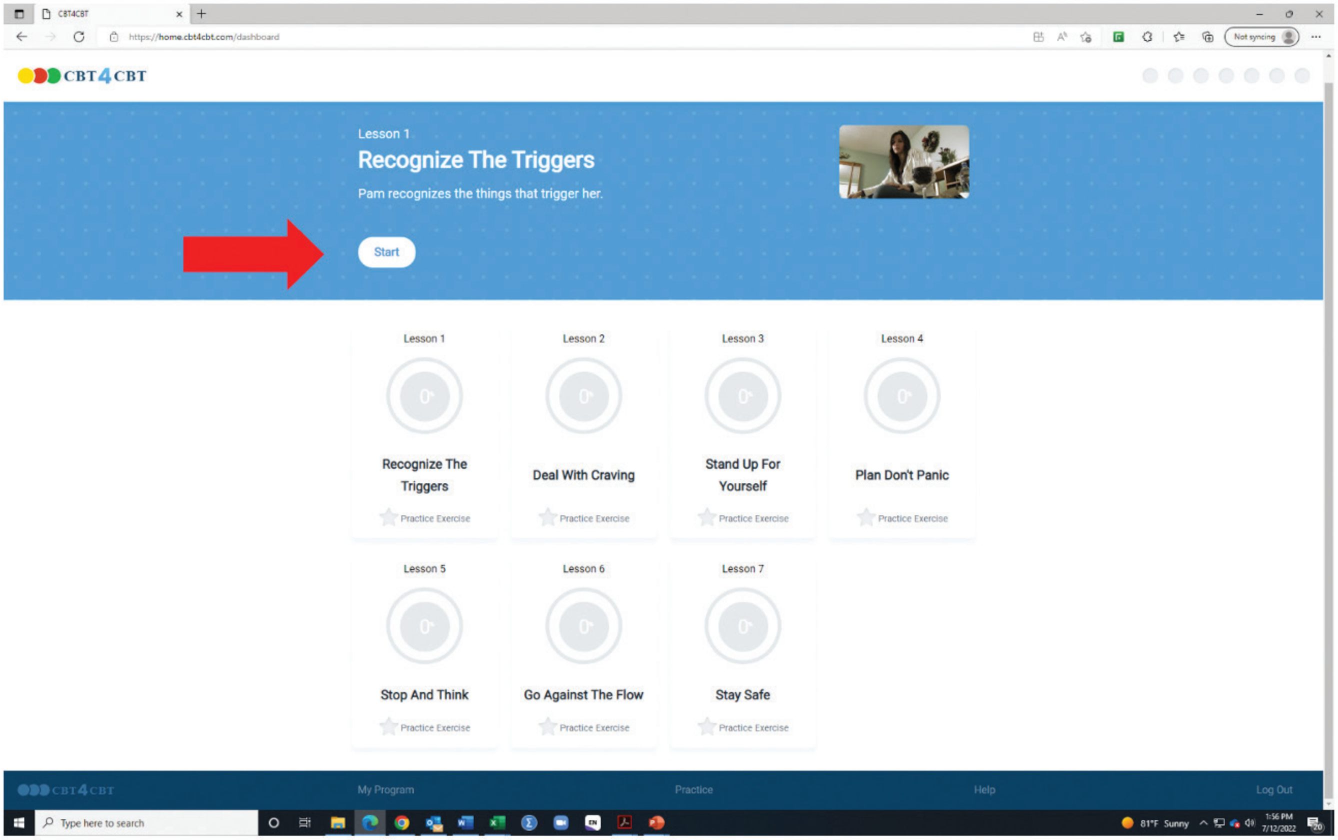

THE NARRATOR WILL APPEAR AND PROVIDE AN INTRODUCTION

SIT WITH PATIENT WHILE THEY WATCH THE 54s INTRODUCTION

"As the narrator said, please click/touch the forward arrow to continue. Do you see it on the screen? This is how you'll move forward or backward through each lesson. You will click on NEXT to move to the next screen. Or you can click on BACK to go back to a prior screen in the lesson."

"Do you have any questions?"

"When you're ready, click/touch the NEXT button to move forward with the lesson. I will leave you to finish the rest of the lesson on your own."

"Please try to complete at least one lesson before you leave the hospital."

IF PATIENT DOESN'T CLICK/TOUCH THE NEXT BUTTON, SHOW THEM WHERE TO CLICK/ TOUCH NEXT TO MOVE ONTO THE NEXT SCREEN

- Last, inquire about internet access at home and identify potential options if not available.

Project ENHANCE_CBT4CBT trifold_English.pdf

Project ENHANCE_CBT4CBT trifold_Spanish.pdf

SUMMARY OF STEP 4 BNI+ Facilitated Provision of MAUD+ CBT4CBT

In Step 4 of the BNI+ Facilitated Provision of MAUD+ CBT4CBT, the HPA assists the patient in exploring a menu of options regarding reducing their alcohol use and engaging in treatment in addition to exploring MAUD, as well as introducing CBT4CBT. The HPA also provides instructions on how to access CBT4CBT and helps them log in for the first time. The HPA encourages the patient to complete the first session at that time or gets a commitment from the patient to complete a session prior to hospital discharge.

Step 4 is ended by scheduling a Booster session (phone call) to occur 2-weeks after they discharge from the hospital and asking the patient if they have any questions about the information or instructions and by thanking them for their time.

VI. Communication with Primary Medical Team

When talking to the medical team:

I spoke with the patient, Mr./Mrs. (include first and last name) *and performed a brief intervention and plan for their alcohol use. The patient expressed interest in knowing more about medication treatment options for alcohol use disorder. They have (or do not have) prior experience with medications. They expressed interest in (naltrexone, acamprosate or disulfiram)* (as applicable).

If you have any questions about which medication to consider for this patient, please refer to the research trifold or the alcohol use disorder care signature pathway in Epic for guidance. You can also contact *** if you have questions.

Project ENHANCE Clinician PDF Flyer.pdf

Smart Web communication with the primary team

1. Go into "Smart Web" paging directory (in Epic, top right corner header)
2. Ask them via text from your primary cell phone if you can approach the patient to discuss the study
3. After patient agrees to study, go back to Smart Web and page them about patient interest and confirm patient enrollment in study and study arm
4. For HPA, if patient interested/not interested etc. in medication – send message via Smart Web like this: *"Please see my Epic note about this patient's interest in medications for alcohol use disorder for the ENHANCE study"* and leave your number for them to ask questions

VII. Documentation in EPIC

BNI Alone

Patient (include first and last name) has enrolled in a research project entitled **Project Enhance - ENhancing Hospital-initiated Alcohol TreatmeNt to InCrease Engagement**. Patients are either randomized to receive a BNI Alone or BNI+ MAUD, or BNI+ MAUD+ CBT4CBT (Computer-Based Training for Cognitive Behavioral Therapy).

Patient (include first and last name) has been randomized to receive the BNI Alone. I spoke with the patient (include first and last name) and performed a brief intervention and plan for their alcohol use. Patient (include first and last name) is interested in ________________ and a referral was made. Patient has an appointment scheduled for ______________________.

BNI+ MAUD

Patient (include first and last name) has enrolled in a research project entitled **Project Enhance - ENhancing Hospital-initiated Alcohol TreatmeNt to InCrease Engagement**. Patients are either randomized to receive a BNI Alone or BNI+ MAUD, or BNI+ MAUD+ CBT4CBT (Computer-Based Training for Cognitive Behavioral Therapy).

Patient (include first and last name) has been randomized to receive the BNI+ MAUD.

I spoke with the patient (include first and last name) and performed a brief intervention and plan for their alcohol use. Patient (include first and last name) is interested in ________________ and a referral was made. Patient has an appointment scheduled for ______________________.

In addition, this writer spoke with the patient about MAUD. The patient expressed interest in knowing more about medication treatment options for alcohol use disorder. They have (or do not have) prior experience with medications. They expressed interest in (naltrexone, acamprosate or disulfiram) (as applicable).

If you have any questions about which medication to consider for this patient, please refer to the research handout or the alcohol use disorder care signature pathway in Epic for guidance. You can also contact *** if you have questions.

[use dotphrase to add ".EJEPROJECTENHANCE" to add Clinician educational materials, see Appendix 11 on page 89 in manual]

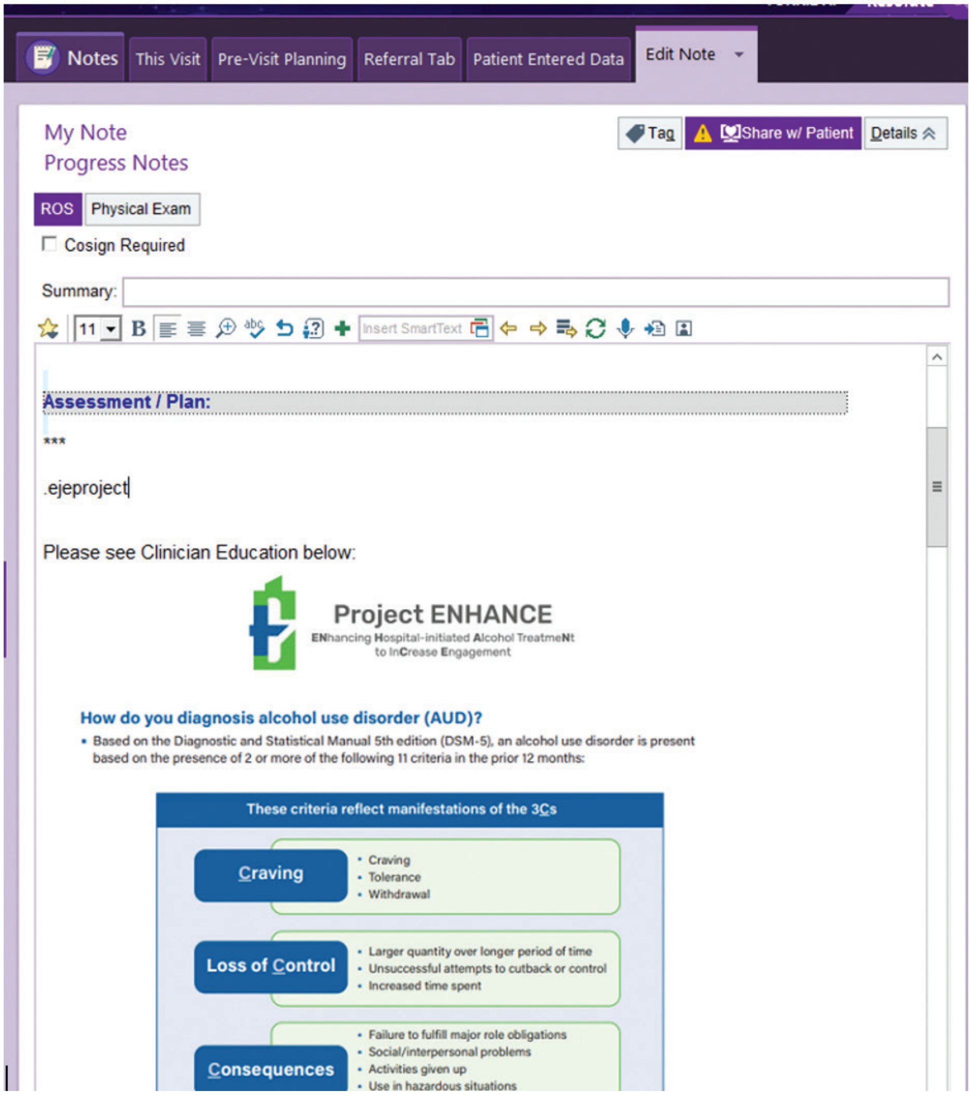

BNI+ MAUD+ CBT4CBT

Patient (include first and last name) has enrolled in a research project entitled **Project Enhance - ENhancing Hospital-initiated Alcohol TreatmeNt to InCrease Engagement.** Patients are either randomized to receive a BNI Alone or BNI+ MAUD, or BNI+ MAUD+ CBT4CBT (Computer-Based Training for Cognitive Behavioral Therapy).

Patient (include first and last name) has been randomized to receive the BNI+ MAUD+ CBT4CBT.

I spoke with the patient (include first and last name) and performed a brief intervention and plan for their alcohol use. Patient (include first and last name) is interested in ________________ and a referral was made. Patient has an appointment scheduled for ______________________.

In addition, this writer spoke with the patient about MAUD. The patient expressed interest in knowing more about medication treatment options for alcohol use disorder. They have (or do not have) prior experience with medications. They expressed interest in (naltrexone, acamprosate or disulfiram).

If you have any questions about which medication to consider for this patient, please refer to the research handout or the alcohol use disorder care signature pathway in Epic for guidance.

Finally, the patient was introduced to CBT4CBT. The patient was provided instruction on how to navigate the program, along with the username and password. Patient was informed to contact *** if questions related to the program surface.

[use dotphrase to add ".EJEPROJECTENHANCE" to add Clinician educational materials, see Appendix 11 on page 89 in manual]

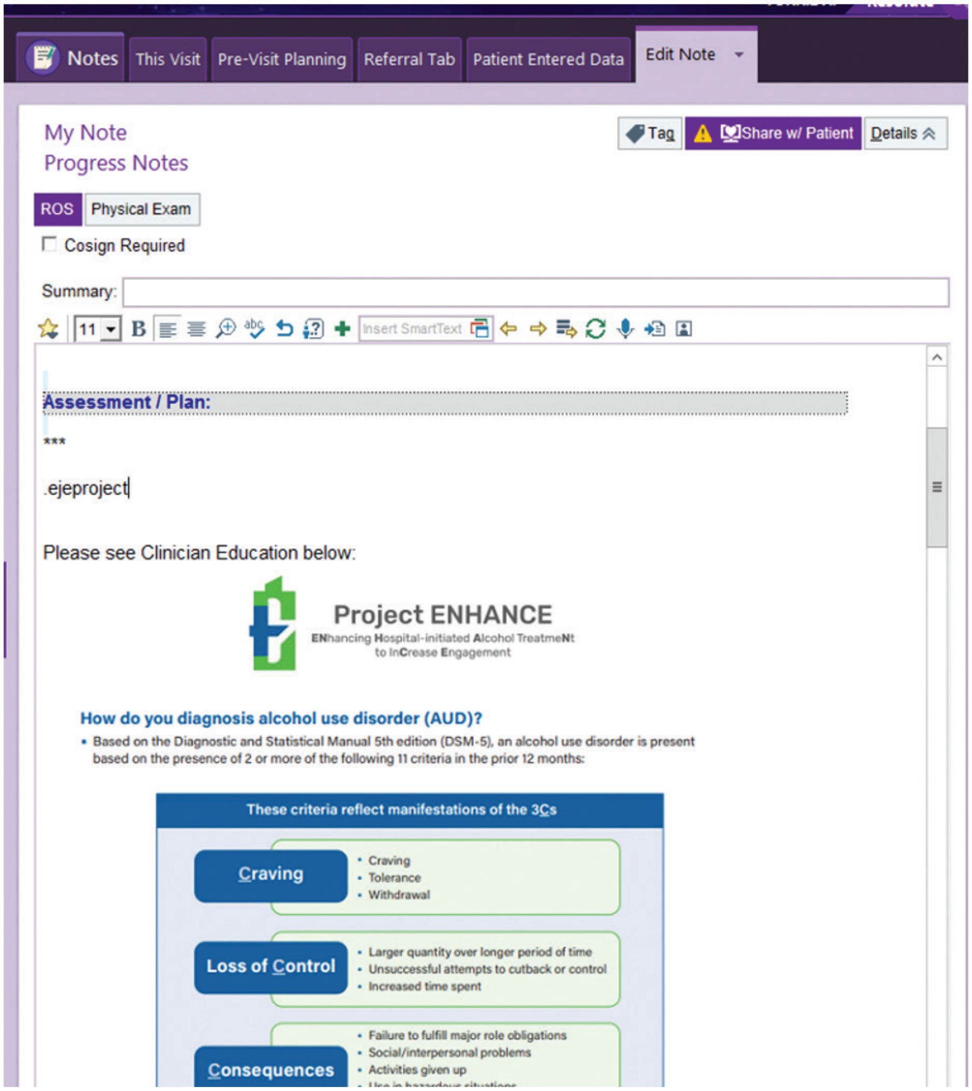

VIII. Brief Negotiation Interview Telephone Booster Session

The Booster session is a brief phone call (15-30 minutes, depending on treatment condition assignment) that should occur **2 weeks after patient is discharged from hospital**.

Goal of the BNI Booster Session:

- To collaboratively help the patient fulfill their plans for engaging in treatment (referral) and/or alcohol reduction agreement.
- To collaboratively put together a new plan for engaging in treatment and/or alcohol reduction agreement if neither was made, or it has been fulfilled.

Overview of the BNI Booster:

- The Booster session is a telephone call done by the HPA lasting approximately 15 minutes. The Booster session aims to build upon the outcome of the initial BNI performed in the hospital.
- The Booster session should remind patients of their previously agreed-upon goals and discuss with the patient their progress and/or barriers to achieving their goals.
- The main components of the Booster for patients who have not made progress toward their drinking agreement and/or who are still not ready to develop an agreement are the same as those for the BNI; for these participants, the Booster will START where the initial BNI left off. The focus for these patients will be building or enhancing motivation to engage in treatment.

Components of the Booster:

I. **Review EPIC note and treatment referral plan prior to call**
II. **Introduction & permission**
III. **Assessment of progress and feedback ~** Positive Reinforcement using affirmations, stigma-reducing language (e.g., alcohol use disorder as a medical condition and not as a defining characteristic) or new goal Setting
IV. **Review skills & establish next step**
V. **Enhance motivation if applicable**
VI. **Reinforce use of resources**

Sample Suggested Booster Dialogue

How to start conversation: *"Hello, my name is* [insert your name here] *and I am the Health Promotion Advocate for Project Enhance, the research study you are involved in. As you may recall, when we last spoke on* [insert date of visit/initial BNI session/specify that the visit occurred during the hospitalization], *we talked about your alcohol use and options for engaging in treatment after your hospitalization.* <PAUSE> *"At that time you also agreed to a follow-up call 2-weeks from when we last met. This is that call."* <PAUSE>

If the patient remembers, continue the dialogue and inquire about questions they may have.

If the patient does not recall, try to help them remember by recalling details of when you initially met.

If Agreement was made at the first session continue as follow:

"May we spend a few minutes talking about how you've done with the treatment referral and alcohol reduction/abstinence goal you agreed to on [insert date]*?"*

If the patient has no recollection of the agreement/referral, ask *"Would it be okay if I went over what we discussed when we last met?"* Trigger memory with items that were discussed during BNI.

Answer any questions that may surface.

Assess progress:

Reflect the patient's statement above and assess progress with the following.

"What progress have you made with the goals you made [reflect goals]*?"*

Reflect and move on to scenario that best applies to the context. See scenarios below.

If no agreement was made at the first visit:

Ask: *"Tell me how you have been doing with respect to your drinking since we last spoke?"*

Reflect and continue.

Summarize current drinking behavior/use.

For example, *"From what I understand, you have been drinking* [insert drinking quantity and frequency reported from above]." <PAUSE>.

Ask: *"Is this pattern different than it was prior to your hospitalization?"*

IF YES, ask *"how so?"* Reflect and explore what has changed.

IF NO, go to Scenario 4 listed on page 39.

Scenario 1:

IF the patient has achieved goals with respect to treatment engagement and drinking reduction/ abstinence, **THEN** ask about and affirm/reinforce reduction methods that they have used to do so.

Evoke/discuss on strategies for preventing return to alcohol use and their experience/plans for engaging in treatment.

Affirm by saying the following, *"You have been working really hard to meet your goals. How do you plan to keep it up?"* Other prompts to consider, *"What barriers do you think you might encounter?"* **Or** *"How can I support you in addressing them so you can keep meeting your goals?"*

Reflect and continue.

Evoke and set goals to maintain this new behavior. For example, *"What are additional strategies that you can implement to help you continue to work on your progress/meeting goals/improving your health?"*

Scenario 2:

IF the patient has achieved treatment engagement goal, but is still drinking above National Institute on Alcohol Abuse and Alcoholism (NIAAA) guidelines or not abstinent **THEN** ask about and reinforce methods they used to do so, BUT also attempt to devise new agreement and new strategies for lowering drinking to below NIAAA guidelines levels or achieve abstinence.

Affirm changes made and evoke new strategies for lowing drinking. For example, *"You're a goal-oriented person and kept your word. What else can you do to continue decreasing your drinking?"*

Reflect, pull for change talk, and continue.

IF patient refuses new agreement, use motivational techniques (e.g., Readiness Ruler, *"What might make you decide to change your drinking?"*) to point them in that direction and to consider such a change in the near future.

Affirm the changes that they have made. Pause for 5 seconds.

"How ready are you to take the next step?" (See BNI Manual)

Scenario 3:

IF the patient has reduced drinking, but has NOT achieved the agreement regarding engagement in treatment, **THEN** assess whether this is a result of: motivation (e.g. patient does not think it is a problem), logistical barriers (e.g., transportation, work schedule, childcare issues), or stressors/ concrete obstacle (e.g. loss of job, entitlements, relationship problems, etc). The key here is to tap

into lived experience. For example, the HPA may ask, *"What in your life is making it hard to reach the agreement?"* **Or** *"Tell me a little more about your everyday life."*

The HPA may want to affirm reduction in drinking and use motivational techniques to enhance motivation for engaging in treatment.

Based on the assessment, offer either motivational enhancement, or discuss problem solving to address barriers to treatment **AND** refer to additional services if necessary.

Either one of the following approaches can be taken:

Affirmations must be specific, genuine, and positive. An example is, *"You have worked very hard to reduce your alcohol consumption."* Follow up affirmation with importance ruler, *"On a scale from 1 to 10, how important is it for you to meet the goals that we agreed on?"* (to enhance motivation and to consider such a change in the future).

Or

"You are seeing the benefits of the changes you have made." Pause for 5 seconds. *"How ready are you to take the next step to achieve that initial agreement you made at the first visit?"*
(See BNI Manual)

Scenario 4:

IF there is no progress toward the agreement or if no agreement was made at the first visit, **THEN** suggest (ask for permission prior to sharing suggestion) developing a new reduction agreement (Please note: the new agreement may be less difficult than original) and referral agreement. Close with asking the patient what their thoughts are about the plan moving forward. This needs to be done collaboratively. Make sure to screen/assess for everyday barriers.

Negotiate new goals ~ this new agreement should be different and easier, with a time-limited goal (i.e., focus on next several weeks). (See BNI Manual)

Review prior consequences of drinking and relate current consequences or lack thereof to current drinking.

Assess readiness to change with Readiness Ruler (See BNI Manual – Enhance Motivation).
See examples above.

Summarize conversation – current progress and next steps.

Answer any questions.

"Thank you for your time."

Goal of the BNI+ Facilitated Provision of MAUD Booster Session:

- To collaboratively assist the patient, fulfill their plans for engaging in treatment, and adherence to MAUD.
- To collaboratively put together a new plan for MAUD if one was never made.

Overview of BNI+ Facilitated Provision of MAUD:

- The BNI+ MAUD Booster session is a telephone call done by the HPA that builds on the BNI Booster lasting approximately 20-25 minutes.
- The goal of the BNI+ MAUD Booster session is to remind patients of their previously agreed upon goals related to MAUD, assess progress, or barriers to achieving goals.

Sample Suggested Booster Dialogue

Once the BNI Booster is complete, follow with the next statement.

Scenario 1:

If patient agreed to medication continue as follow:

"Now that we have discussed treatment progress/barriers, is it okay if we also talk about how you are doing with [insert medication name]*?"*

If the patient agrees, ask *"Tell me how you have been doing with taking* [insert medication name]", **Or** *"What benefits have you noticed since starting [name of medication]"* **Or** *"How have you felt since starting* [name of medication]."

Reflect statement, continue to pull for change talk, and explore change.

Make sure to affirm progress. "*You're being proactive and working hard to meet your goal. How do you plan to continue?"*

Reflect and continue.

Evoke and set goals to maintain the behavior change. For example, *"What additional resources may you need to help you continue to work on your progress/improving your health?"*

Scenario 2:

If patient agreed to medication but has not started medication (or has stopped taking medication) continue as follow:

The HPA may want to affirm positive changes and use motivational techniques to enhance motivation for adherence to medication.

Based on patient and context, offer either motivational enhancement, or discuss problem solving to address barriers to medication adherence.

Either one of the following approaches can be taken:

"What do you recall from our last discussion related to medication that can help you with your alcohol use?" If the patient recalls information, reflect and continue to pull for change talk. If the patient does not recall information, ask for permission to share benefits of medication. If they respond with *"Yes"* share the benefits to medication. Once information has been shared follow it up with the next question, *"What do you make of the information just shared?"*

If the patient continues to express ambivalence about starting medication, ask *"What would need to happen in order for you to start medication?"*

Or

"You are seeing the benefits of the changes you have made." Pause for 5 seconds. "*How ready are you to take the next step to achieve that initial agreement you made at the first visit regarding medication?"*

Scenario 3:

IF no agreement was made at the first visit for medication, **THEN** suggest (ask for permission prior to sharing suggestion) developing a new medication agreement. Close with asking the patient what their thoughts about the plan moving forward are. This needs to be done collaboratively.

Review prior concerns about medication, validate, and pull for change talk.

Assess readiness for medication with Readiness Ruler *"On a scale from 1 to 10, how ready are you to start medication for alcohol use?"*

Summarize conversation – current progress and next steps.

Answer any questions.

Communicate to the patient that this is the last session with the HPA. If they have further questions or concerns related to their care to follow-up with their primary care provider. Finally, express to the patient that any study related concerns and/or follow-ups will be addressed by the research coordinator.

"Thank you for your time."

Goal of the BNI+ Facilitated Provision of MAUD+ CBT4CBT Booster Session:

- To collaboratively assist the patient, fulfill their plans for engaging in CBT4CBT.
- To collaboratively put together a new plan if CBT4CBT has not been started.

Overview of BNI+ Facilitated Provision of MAUD+ CBT4CBT:

- The BNI+ MAUD+ CBT4CBT Booster session is a telephone call done by the HPA that builds on the BNI+ MAUD Booster lasting approximately 25-30 minutes.
- The goal of the BNI+ MAUD+ CBT4CBT Booster session is to remind patients of their previously agreed upon goals related to participating in CBT4CBT, assess progress, or barriers to achieving goals.

Sample Suggested Booster Dialogue

Once the BNI+ MAUD Booster is complete, follow with the next statement.

Scenario 1:

If the patient started CBT4CBT continue as follow:

"Now that we have discussed treatment progress/barriers, medication for alcohol use, is it okay if we talk about your progress with CBT4CBT?"

If the patient agrees, ask *"Tell me how it's been going with CBT4CBT*" **Or** *"How helpful have you found the program"* **Or** *"What is something new that you have learned from the program and how have you implemented the skill(s) learned?"*

Reflect statement, continue to pull for change talk, and explore benefits.

Make sure to affirm progress. *"You've made significant progress and are implementing what you are learning. How do you plan to continue?"*

Reflect and continue.

Evoke and set goals to maintain the behavior change. For example, *"How else do you see this program helping you?"*

Scenario 2:

If patient has not started the program continue as follows:

The HPA may want to affirm positive changes and use motivational techniques to enhance motivation to start the program.

Based on patient and context, offer either motivational enhancement, or discuss problem solving to address barriers to starting the program.

Either one of the following approaches can be taken:

Explore benefits by asking, *"How do you think the program may help you reach your goals"* **Or** *"How may the program add to what you are already doing?"*

If the patient continues to express ambivalence about starting CBT4CBT, ask *"How can I support/ assist in getting your started with the program?"*

Or

Review prior concerns or barriers to starting the program, validate, and pull for change talk.

Explore barriers by asking, "*What has gotten in the way of you accessing the program?"*

Discuss problem solving to address barriers (e.g., limited internet access, lack of time and/or privacy). Set goals for how to address.

Assess readiness to start the program with Readiness Ruler *"On a scale from 1 to 10, how ready are you to start the program?"*

Summarize conversation – current progress and next steps.

Answer any questions.

Communicate to the patient that this is the last session with the HPA. If they have further questions or concerns related to their care to follow-up with their primary care provider. Finally, express to the patient that any study related concerns and/or follow-ups will be addressed by the research coordinator.

"Thank you for your time."

IX. Additional Motivational Strategies

Refrain from Directly Countering Resistance Statements

For example, the patient may say "How can I have an alcohol problem when I drink less than all my buddies?" You can reply...

In your friend group you consume the least or compared to your buddies you drink the least. It is also important to note that alcohol use can vary between patients and that it is worthy of further assessment and discussion, within the context of this brief interview and advice from treatment professionals.

Focus on the Less Resistant Aspects of the Statement

For example, the above patient may be wondering about how much their alcohol use is a problem. The response might be to restate his concern and ask about his level of alcohol use, which is the less resistant part of the statement. "It sounds like you're confused about how you could have an issue with your alcohol use if you use less than all your friends. I'd like to explain this to you." *(And remember, this is a statement NOT a question, so the intonation should turn down at the end of the remark).*

Restate Positive or Motivational Statements

For example, if a patient says: "You know, now that you mention it, I feel like I have been drinking more than I wanted to lately," the HPA could say, "It sounds like you are concerned that your alcohol use is out of your control." This serves to reinforce the patient's motivation – even if the motivational statement is a relatively weak one. If the patient says, "I guess I might have to change my alcohol use" this could be restated as "It sounds like you've been thinking about changing your alcohol use".

Other Helpful Hints

Encourage patients to think about previous times they have cut back or been abstinent, even for a few days.

Praise patients for their willingness to discuss such a sensitive topic, their willingness to consider change, and their courage for considering treatment. Acknowledge how hard it is to find treatment options.

View the patient as an active patient in the intervention.

X. Common Problems

Certain problems may occur during the course of the intervention steps…

Refusal to Engage in Discussion regarding Alcohol Use

Most patients will agree to discuss the topic, but in the unlikely event that someone outright refuses to discuss it at all, tell the patient that you will respect their wishes and that all you will be doing is giving them 3 pieces of information:

ALCOHOL:

1. Their drinking exceeds lower-risk drinking limits (or is harmful);
2. Lower-risk drinking limits recommended for patient's age and sex; and,
3. You are concerned and that they should cut down to lower-risk drinking limits to avoid future harm (Steps 2 and 4 only).

Refusal to Self-Identify Along the Readiness Ruler

When this happens, it is usually a problem with understanding the numbers. There are several ways of dealing with this:

1. Anchor the numbers with descriptors, such as "1" means not ready at all or 0 per cent ready, and "10" means completely ready or 100% ready to change.
2. Ask "What would make this a problem for you?" **Or** "How important is it for you to change any aspect of your alcohol/drug use?"
3. Discussion of Pros and Cons (See Appendix 3).

Unwilling to Associate Hospitalization with Alcohol Use

Don't force the patient to make the connection, but be sure that they hear that in your opinion there is a connection. However, this connection may not be the thing that ultimately motivates the patient to change. If this happens try to find some other negative consequence of alcohol use that the patient can agree is bothersome enough to consider decreasing or stopping their alcohol use.

Not Ready to Bring Drinking Patterns Down to Lower-Risk Limits

Tell the patient that the best recommendation is to cut back to lower-risk drinking limits, but that any step in that direction is a good start. The patient's goal is then written on the alcohol use agreement. Regardless of the individual goal, the patient also receives the HPA's advice for lower-risk drinking on the patient health information trifold.

XI. Appendices

Appendix 1: The Spectrum of Alcohol Use

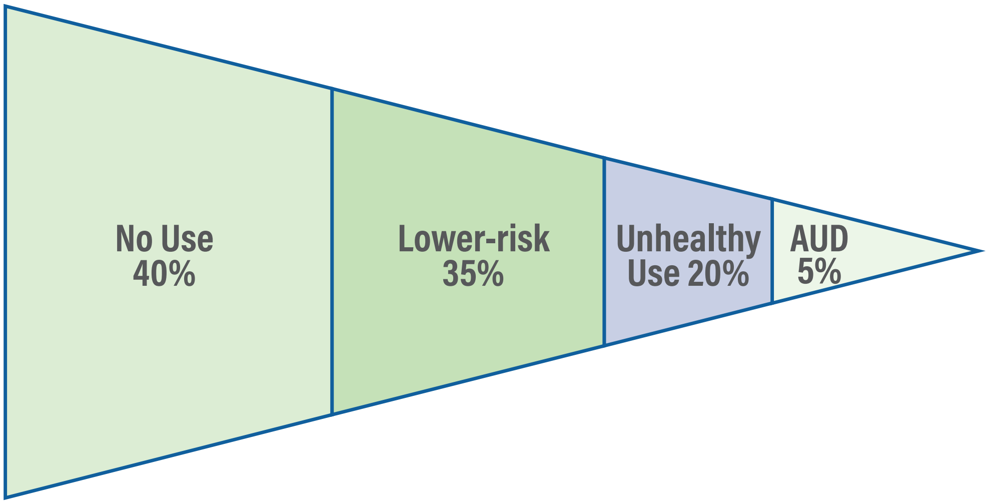

Types of Alcohol Use

**No Use**	Drink no alcohol.
**Lower-risk**	Drink within NIAAA guidelines. Alcohol use does not affect health or result in problems.
**Unhealthy Use**	Exceed NIAAA consumption guidelines. Alcohol use that puts them at risk for injury/illness or social problems, or associated with current problems.
**Alcohol Use Disorder**	Meets DSM-5 criteria and has alcohol withdrawal, alcohol tolerance, craving, loss of control, and consequences from alcohol use.

Appendix 2: The Readiness to Change Ruler

(Referred to in Step 3 of the BNI on page 14)

Readiness Ruler

1	2	3	4	5	6	7	8	9	10

Not ready at all **Completely Ready**

Appendix 3: Pros and Cons for Alcohol Use & Treatment

(Referred to in Step 3 of the BNI on page 14)

Reasons to Quit or Cut Down on Alcohol Use

To live longer, and feel better
To consume fewer empty calories (alcohol has no nutritional value)
To sleep better
To be less likely to have a stroke
To improve blood pressure control
To reduce the possibility of death from liver disease
To prevent problems with medications
To decrease the likelihood of falls or other injuries
To prevent memory loss that may lead to loss of independence
To be able to care for myself longer
To be a better parent or grandparent
To reduce the possibility that I will die in a car crash
Other reasons: ____

Reasons for Alcohol Use

I enjoy the taste
It enhances meals
For pleasure in social situations
To more easily socialize
Other people expect that I will drink with them
To relax or relieve stress
To cope with feelings of anger
To cope with feelings of boredom
To deal with momentary feelings of depression
To deal with momentary feelings of loneliness
To deal with feelings of frustration
To relieve the stress of arguments with family members or friends
It's something I do when I'm smoking
It's something I do when I'm watching TV
It's something I do with certain friends or relatives
To help me sleep
To relieve pain

(continue)

Appendix 3: Pros and Cons for Alcohol Use & Treatment *(continued)*

Reasons to Enter Alcohol Treatment

To spend less money
To avoid constantly seeking alcohol
To do a better job at home
To do a better job at work
To live longer, and feel better
To sleep better
To be able to care for myself longer
To be able to care for my children better
To be a better son/daughter, wife/husband, parent, or grandparent
To reduce the possibility that I will die
Other reasons: ____

Reasons for Ongoing Alcohol Use

Boredom
Habit
I enjoy the taste or the way that it feels
For pleasure in social situations
To more easily socialize
Other people expect that I will drink when I am with them
To relax or relieve stress
To cope with feelings of anger
To cope with feelings of anxiety
To cope with feelings of boredom
To deal with momentary feelings of depression
To deal with momentary feelings of loneliness
To deal with feelings of frustration
To relieve the stress of arguments with family members or friends
It's something I do when I'm watching TV
It's something I do with certain friends or relatives
To help me sleep
To relieve pain
To make me feel better
Other reasons: ____

Appendix 4: Alcohol Use Agreement & Referral Agreement

(Referred to in Step 4 page 16)

ALCOHOL USE AGREEMENT

Date:______________________

I,____ , agree to the following goals:

Alcohol

_______ I will not drink at all **Or**

Number of drinks week:__________

Number of drinks per occasion:_________

Follow-up_________________________With________________________________

Patient Signature: ____

REFERRAL ACCEPTANCE

Date:______________________

I,____ , agree to accept the following treatment referral to (name of referral location):

____

____

Patient Signature: ____

Appendix 5: Case Example of BNI Dialogue For Alcohol Use Reduction

SPEAKER	DIALOGUE	PROCEDURE
**HPA**	Hello, I am _______. I am a HPA with the study. Would you mind spending a few minutes talking about your use of alcohol?	**RAISE THE SUBJECT**
**Patient**	Ok, like what?	
**HPA**	From what I understand you were drinking and were involved in a car crash. You told the RA that you drink 2-3 days a week and usually have 6-8 beers per occasion. I am concerned because that level of drinking can put you at risk for illness or injuries, such as why you are here today.<br>What connection do you see between your drinking and your hospital stay?	**PROVIDE FEEDBACK**<br>Make connection
**Patient**	None really. I mean, I really had the right of way. I had a few beers. What is the problem with that? I can hold my alcohol well. He ran into me. You know that intersection between Grand and College Ave. I was going south on College and he just smacked right into me. I didn't see him at all. I am in kind of a rush. I need to get out of here, but it wasn't my fault.	
**HPA**	I believe that it was not your fault. I know that busy intersection. However we know that drinking even small amounts such as 1 or 2 drinks can reduce your reaction time. As you know, we avoid crashes almost every day. Drivers run stop signs, backup without looking etc. At that very intersection there are near misses everyday. Do you think that you might have seen that other car approaching and avoided the crash if you had not been drinking? I don't know for sure, I was not there, but it is one thing I would like you to consider.	
**Patient**	Well, I said that I didn't see him at all. I didn't see him until the crash.	
**HPA**	So one thing, you might have seen him if you weren't drinking any amount. It is clear that legally you had the right of way. I am also concerned about the amount you drink. Based on a large amount of research and national information we know that drinking above certain levels puts you at risk for injuries and illness. For your age and sex that means the upper limits of low risk drinking are no more than 14 drinks per week, and no more than 4 drinks on any occasion. A standard drink is one 12 ounce can of beer, 5 ounces of wine or 1 1/2 ounces of distilled spirits.	
**Patient**	Yeah, I guess I am over that.	
**HPA**	Well now that we have discussed the risks of further injury when drinking over the recommended amounts, how ready are you to change any aspect of your drinking?	**ENHANCE MOTIVATION**<br>Readiness to change
**Patient**	I don't know, maybe a 5.	

(continue)

Appendix 5: Case Example of BNI Dialogue For Alcohol Use Reduction *(continued)*

SPEAKER	DIALOGUE	PROCEDURE
**HPA**	OK, so that is good, you are halfway or 50% there. Why not less? In other words, why did you not pick a 1 or 2? What are some reasons why you think some changes need to be made?	Develop discrepancy
**Patient**	Well, I am here I guess, and I can tell that my neck and back are really going to hurt tomorrow. But I really do like to drink with my friends. Normally I do not drink and drive, but I needed to be somewhere after, so I drove myself.	
**HPA**	So you already know that drinking and driving is not a good idea and that was a rare event for you. But rare events can sometimes lead to consequences, like today. So I guess you are ready because you don't think that it's a good idea to drink and drive. On the other hand you enjoy drinking with your friends. Any disadvantages to that?	Reflection
**Patient**	We normally go out on Friday and Saturdays. Sometimes on Thursdays and then I'm a little late to work on Friday. It takes the morning and lots of coffee to clear my head.	
**HPA**	So what I hear your saying is that there are two reasons why you are dissatisfied with your drinking. First is that you ended up in the ED and will probably have some muscles aches and pains for a few days, and second that sometimes you are slow at work. That could cause you trouble I suspect with your boss. In addition I have given you some information regarding the risks of drinking over the recommended limits. So, where does that leave you now? (or what is the next step?) What agreement could you make between you and yourself regarding your drinking levels?	**NEGOTIATE & ADVISE**<br><br>Summarize<br>Negotiate goal
**Patient**	Well, I'm definitely not going to drink and drive. That is a big deal because even though I thought I could, I probably can't. I don't know about the limits. I can stay within 14 a week, but I don't know about the 4 at a time. I will try but it is often a long game we are watching.	
**HPA**	So no more drinking and driving, and you are going to try to keep it to 4 beers per occasion, knowing that it's tough at times but you are willing to try.	
**Patient**	OK	
**HPA**	I'd like to follow up with you in two weeks. Is it okay if I give you a call to see how you are doing? I would also recommend that you follow-up with your primary care doctor and discuss how you are doing with the agreement.<br><br>Thanks for your time.	Follow-up<br><br>Thank patient

Appendix 6: Plan to Promote Health

Goal is to further develop and strengthen understanding of triggers for alcohol use;
Identify the thoughts, feelings, and behaviors associated with those triggers;
Identify possible interventions to address those triggers;
Identify additional sources of support.

These are some of my triggers (people, places, and things which might make me think of drinking):

-
-
-

These are some of the thoughts, feelings, and behaviors which can contribute to urges or cravings to drink:

-
-
-

What can I do when I start to have these feelings. Name healthy coping skills (activities and behaviors to get my mind off drinking): Example: periodic self-check in or mood check.

-
-
-

Specific actions I will take to decline alcohol use in the future:

-
-
-

People I can reach out to for support (family, friends, fellowship, etc):

-
-
-

My continued plan of care:

-
-
-

Appendix 7: BNI – MAUD – CBT4CBT Checklists

BNI Alone Checklist

1. Ask the patient for permission to discuss alcohol use ☐ yes ☐ no

HOW TO START CONVERSATION:

"May we spend a few minutes talking about your use of alcohol." <PAUSE>

If the patient response is *"YES,"* use the following statement.

"I want to talk about how it's affecting you and how we might be able to help."

Follow the statement with the next questions.

"Tell me how you are feeling right now? When is the last time you drank alcohol?"

If the patient response is *"NO"* use the following statement.

"When would be a great time for us to discuss your alcohol related concerns?"

Or

"We don't have to talk about your alcohol use, we can talk about other concerns that you may have."

*Make sure to **avoid a non-judgmental stance**, acknowledge the situation, be mindful of cultural values for the racial and ethnic minoritized individual, and focus on engagement. Reflective listening is KEY.

2. Review patient's drinking patterns ☐ yes ☐ no

Reflect patients last statement and follow with the next comment.

"From what I understand you are drinking (fill in amounts from screening)*."*
BUT DO NOT RE-SCREEN.

Follow the statement above with one of the next questions.

"How concerned are you with your alcohol use, if at all?" **Or** "*What concerns has it caused you, if any?"*
Or *"How concerned or worried is your family with your drinking?"* <PAUSE>

Make sure to reflect the patient's statement. For example, "*it sounds like there is some concern with your alcohol use."*

3. Express concern about these patterns ☐ yes ☐ no

Reflect the participant's statement above and assess alcohol use and medical illness connection

Appendix 7: BNI Alone Checklist *(continued)*

with the following question:
"What connections might you see between your use of alcohol and your medical condition?"

Reflect the participants statement and follow it up with the next question.

"Has there been a time in your life when you stopped drinking or cut back in the past?"

"If 'YES,' please tell me about how you did it?"

"If 'NO,' tell me what has been helpful in your life in overcoming obstacles? **Or** *"In the past, how have you managed a difficult situation."*

IF PATIENT STATES ANY ACCURATE & RELEVANT CONNECTION(S),
reiterate them, acknowledge (affirm) the patient's good insight, and give additional relevant facts on medical risks related to alcohol use.

IF PATIENT DOES NOT STATE ANY ACCURATE & RELEVANT CONNECTIONS,
MAKE the connection based on general caution regarding alcohol use.

"From research we know that any alcohol use compromises decision making and health behaviors. In addition, we know that any alcohol use can be harmful to health."

4. Ask about a connection ☐ **yes** ☐ **no**

Reflect participant's comment above and follow with the next question if participant did not already acknowledge a connection between alcohol and this hospitalization.

"What (if any) connection is there between your alcohol use and this hospitalization?" <PAUSE>

See next steps below.

5. Reflect the patient's statement ☐ **yes** ☐ **no**

IF PARTICIPANT SEES ANY ACCURATE & RELEVANT CONNECTION, reiterate (reflect) what they have said.

6. Provide medical facts and information that there is a connection between the hospitalization and drinking ☐ **yes** ☐ **no**

Based on the information obtained above, follow with one of the options below.

SAMPLE: *"You're right,* [reiterate patient's connection] *... that's a good connection to make."*

IF PATIENT DOES NOT SEE AN ACCURATE CONNECTION, then make one using facts (if applicable).

This is a key step component of respecting autonomy: ***Ask for permission to share: Is it okay if I***

Appendix 7: BNI Alone Checklist *(continued)*

share an observation? If yes, follow the sample on next page.

SAMPLE: *"One of the things that we see as a connection is that your patterns of alcohol use may have contributed to your fall, physical discomfort, or whatever they're in the hospital for."* (if relevant)

Follow the statement with: *"What do you make of that?"*

IF THERE ACTUALLY IS NO CONNECTION between the hospitalization and alcohol use, ask about any potential connections between alcohol use and overall health issues.

7. Inform patient of NIAAA guidelines by using the pamphlet or card ☐ **yes** ☐ **no**

Hand patient trifold Project ENHANCE BNI Trifold.pdf

Whether a connection between hospitalization and drinking has been made, the next step is to provide information regarding the spectrum of alcohol use and recommendations regarding safer drinking limits; introduce relevant content on the BNI trifold.

Ask for permission to share information on NIAAA guidelines: *"Is it okay if I review with you what is considered safer drinking limits for your age and gender?"*

If the participant's response is *"YES"* proceed with sharing information. If they say *"NO",* say – "*tell me what you usually drink."*

If the participant's response was *"YES"* and drinking norms have been shared ask the following question: *"What do you make of the information just shared."*

This is where we want to bring it all together and use a summary. For example, *"Based on what we discussed so far, there seems to be some concern with your drinking and there is an indirect connection between your alcohol use and current hospitalization. Did I get that correct?"*

If the participant's response is *"YES,"* move on to the following statement.

"So, in order to help you with all of this, I would like to talk with you a bit further about how we might be able to help you reduce the concerns associated with your alcohol use. For example, I want to encourage you to consider that there are a wide variety of treatment options available and that I am prepared to go over them with you." (See and show treatment referral list.)

Ask for permission to share treatment options: *"Is it okay if I share some options with you?"*

If *"YES,"* move forward with discussion treatment options (reference trifold)

Explain different types of treatment options available

Medically Supervised Withdrawal Management (traditionally known as "Detox")

Appendix 7: BNI Alone Checklist *(continued)*

Partial Hospitalization Program (PHP)
Intensive Outpatient Program (IOP)
AA (Alcoholics Anonymous) and Narcotics Anonymous (NA)

To explore more positive thoughts about treatment ask *"Why might you decide to go? Have you thought about going before? Why might it be important to seek treatment now? What were some of the reasons why? Why is it important to have this conversation now?"*

"Would you mind If I gave you some possible reasons?"

If patient says, *"YES,"* then reflect on the possible consequences of treatment.

If patient says, *"NO,"* then discuss possible benefits of treatment (e.g., reversing the negative effects of physical alcohol dependence discussed in Steps 1 & 2).

Offer summary reflection of patient's reasons for starting or considering treatment, ending with a reinforcement of the patient's autonomy… *"Ultimately, the decision to seek treatment is up to you."*

Close with: *"If you decide to accept our recommendation, you would be taking the first step towards addressing the problems connected with your alcohol use that we've been discussing."*

8. Ask patient to identify readiness to change on Readiness Ruler ☐ **yes** ☐ **no**

Show card

Start with: *"Now that we have discussed treatment options, on a scale from 1-10, where 1 is 'not at all' and 10 is 'right now', how ready are you to engage in alcohol treatment after your hospitalization?"*

9. Ask why they choose that number and not a lower one ☐ **yes** ☐ **no**

If patient says:
- ≥ 2, ask *"Why did you choose that number and not a lower one?"* (i.e., *"What are some reasons you would engage in treatment?" "What else?"*

This is intended to pull for change talk, a patient's own argument for change. The change talk is then reflected. See below.

If the patient's response is 1 or unwilling, ask *"What would it take for that "1" turn into a "2"?* **Or** *"Imagine you did go to treatment; how could that be helpful to you?"* **Or**

"What would have to happen for you to be ready? "How important would it be for you to prevent that from happening?"

Reflect/reiterate positive reasons for change.

Discuss pros and cons of treatment. (See Appendix 3 on pages 48-49 in manual)

Appendix 7: BNI Alone Checklist *(continued)*

10. Reflect patients' statements regarding change ☐ yes ☐ no

Then REFLECT on their reasons and reinforce with clinical information (e.g., *"Treatment does in fact work to help reduce and eliminate use, as well as the problems connected with it."*

Take the participant's answer from the above question and ask: *"What makes that reason important to you?"*

11. Elicit response (How does all this sound to you?) ☐ yes ☐ no

Reiterate what participant says in above and say, *"What's the next step?"* [PAUSE] *"Are you accepting the referral?"*

12. Negotiate the goal (What would you like to do?) ☐ yes ☐ no

IF *"YES"* to treatment, skip to bottom "Secure Referral".

IF *"NO,"* reiterate reasons participant gave above and <PAUSE>.

IF STILL *"NO,"* then ask if the participant might consider it and give advice below for patient to have, even if they do not want to consider it, and then SKIP TO "Provide Handouts".

13. Give advice if necessary ☐ yes ☐ no

Based on the response above, follow with: *"If you leave the hospital with a plan for continued treatment for your alcohol use, you will be on the road to better health.* (see facilitated referral next page.) *Based on what you told me and what we know about alcohol use disorder, I think you should…", "it may be a good idea to address this now…"*

"Remember what we discussed in terms of improving your health."

Once a *"YES"* has been secured, follow with: *"Which of these options might you be interested in?"* [SHOW LIST]

14. Summarize (This is what I've heard you say…) ☐ yes ☐ no

15. Have patient fill out agreement card ☐ yes ☐ no

"Let's complete this referral agreement together which will reinforce your decision to seek treatment. This is really an agreement between you and yourself."

"Ok, based on your preferences, your insurance information and availability of programs, I can make a few phone calls to schedule an appointment for you. You may even be able to speak to someone while you are here in the hospital."

"Once I make a few calls, I will come back and we can update the referral agreement. How does that sound?"

Appendix 7: BNI Alone Checklist *(continued)*

16. Upon return, review information with the patient and update the referral agreement

☐ yes ☐ no

Provide summary
Update form with appointment date and time
Get signature
Ask if they have any questions: *"What questions do you have?"*

17. Thank the participant for their time ☐ yes ☐ no

Comments: ____

____

____

____

____

Start time: ____________________ End time: ____________________

Appendix 7: BNI – MAUD – CBT4CBT Checklists

BNI+ MAUD Adherence & Competence Checklist

1. Ask the patient for permission to discuss alcohol use ☐ yes ☐ no

HOW TO START CONVERSATION:

"May we spend a few minutes talking about your use of alcohol." <PAUSE>

If the patient response is *"YES,"* use the following statement.

"I want to talk about how it's affecting you and how we might be able to help."

Follow the statement with the next questions.

"Tell me how you are feeling right now? When is the last time you drank alcohol?"

If the patient response is *"NO"* use the following statement.

"When would be a great time for us to discuss your alcohol related concerns"

Or

"We don't have to talk about your alcohol use, we can talk about other concerns that you may have."

*Make sure to **avoid a non-judgmental stance**, acknowledge the situation, be mindful of cultural values for the racial and ethnic minoritized individual, and focus on engagement. Reflective listening is KEY.

2. Review patient's drinking patterns ☐ yes ☐ no

Reflect patients last statement and follow with the next comment.

"From what I understand you are drinking (fill in amounts from screening)*."*
BUT DO NOT RE-SCREEN.

Follow the statement above with one of the next questions.

"How concerned are you with your alcohol use, if at all?" **Or** "*What concerns has it caused you, if any?"*
Or *"How concerned or worried is your family with your drinking?"* <PAUSE>

Make sure to reflect the patient's statement. For example, "*it sounds like there is some concern with your alcohol use."*

3. Express concern about these patterns ☐ yes ☐ no

Reflect the participant's statement above and assess alcohol use and medical illness connection with the following question:

Appendix 7: BNI+ MAUD Adherence & Competence Checklist *(continued)*

"What connections might you see between your use of alcohol and your medical condition?"

Reflect the participants statement and follow it up with the next question.

"Has there been a time in your life when you stopped drinking or cut back in the past?"

"If 'YES,' please tell me about how you did it?"

"If 'NO,' tell me what has been helpful in your life in overcoming obstacles? **Or** *"In the past, how have you managed a difficult situation."*

IF PATIENT STATES ANY ACCURATE & RELEVANT CONNECTION(S),
reiterate them, acknowledge (affirm) the patient's good insight, and give additional relevant facts on medical risks related to alcohol use.

IF PATIENT DOES NOT STATE ANY ACCURATE & RELEVANT CONNECTIONS,
MAKE the connection based on general caution regarding alcohol use.

"From research we know that any alcohol use compromises decision making and health behaviors. In addition, we know that any alcohol use can be harmful to health."

4. Ask about a connection ☐ yes ☐ no

Reflect participant's comment above and follow with the next question if participant did not already acknowledge a connection between alcohol and this hospitalization.

"What (if any) connection is there between your alcohol use and this hospitalization?" <PAUSE>

See next steps below.

5. Reflect the patient's statement ☐ yes ☐ no

IF PARTICIPANT SEES ANY ACCURATE & RELEVANT CONNECTION, reiterate (reflect) what they have said.

6. Provide medical facts and information that there is a connection between the hospitalization and drinking ☐ yes ☐ no

Based on the information obtained above, follow with one of the options below.

SAMPLE: *"You're right,* [reiterate patient's connection] *... that's a good connection to make."*

IF PATIENT DOES NOT SEE AN ACCURATE CONNECTION, then make one using facts (if applicable).

This is a key step component of respecting autonomy: ***Ask for permission to share: Is it okay if I share an observation? If yes, follow the sample on next page.***

Appendix 7: BNI+ MAUD Adherence & Competence Checklist *(continued)*

SAMPLE: *"One of the things that we see as a connection is that your patterns of alcohol use may have contributed to your fall, physical discomfort, or whatever they're in the hospital for."* (if relevant)

Follow the statement with: *"What do you make of that?"*

IF THERE ACTUALLY IS NO CONNECTION between the hospitalization and alcohol use, ask about any potential connections between alcohol use and overall health issues.

7. Inform patient of NIAAA guidelines by using the pamphlet or card ☐ yes ☐ no

Hand patient trifold Project ENHANCE BNI Trifold.pdf

Whether a connection between hospitalization and drinking has been made, the next step is to provide information regarding the spectrum of alcohol use and recommendations regarding safer drinking limits; introduce relevant content on the BNI trifold.

Ask for permission to share information on NIAAA guidelines: *"Is it okay if I review with you what is considered safer drinking limits for your age and gender?"*

If the participant's response is *"YES"* proceed with sharing information. If they say *"NO",* say – "*tell me what you usually drink."*

If the participant's response was *"YES"* and drinking norms have been shared ask the following question: *"What do you make of the information just shared."*

This is where we want to bring it all together and use a summary. For example, *"Based on what we discussed so far, there seems to be some concern with your drinking and there is an indirect connection between your alcohol use and current hospitalization. Did I get that correct?*

If the participant's response is *"YES,"* move on to the following statement.

"So, in order to help you with all of this, I would like to talk with you a bit further about how we might be able to help you reduce the concerns associated with your alcohol use. For example, I want to encourage you to consider that there are a wide variety of treatment options available and that I am prepared to go over them with you." (See and show treatment referral list.)

Ask for permission to share treatment options: *"Is it okay if I share some options with you?"*

If *"YES,"* move forward with discussion treatment options (reference trifold).

Explain different types of treatment options available

Medically Supervised Withdrawal Management (traditionally known as "Detox")
Partial Hospitalization Program (PHP)
Intensive Outpatient Program (IOP)
AA (Alcoholics Anonymous) and Narcotics Anonymous (NA)

Appendix 7: BNI+MAUD Adherence & Competence Checklist *(continued)*

To explore more positive thoughts about treatment ask *"Why might you decide to go? Have you thought about going before? Why might it be important to seek treatment now? What were some of the reasons why? Why is it important to have this conversation now?"*

"Would you mind If I gave you some possible reasons?"

If patient says, *"YES,"* then reflect on the possible consequences of treatment.

If patient says, *"NO,"* then discuss possible benefits of treatment (e.g., reversing the negative effects of physical alcohol dependence discussed in Steps 1 & 2).

Offer summary reflection of patient's reasons for starting or considering treatment, ending with a reinforcement of the patient's autonomy... *"Ultimately, the decision to seek treatment is up to you."*

Close with: *"If you decide to accept our recommendation, you would be taking the first step towards addressing the problems connected with your alcohol use that we've been discussing."*

8. Ask patient to identify readiness to change on Readiness Ruler ☐ **yes** ☐ **no**

Show card

Start with: *"Now that we have discussed treatment options, on a scale from 1-10, where 1 is 'not at all' and 10 is 'right now', how ready are you to engage in alcohol treatment after your hospitalization?"*

9. Ask why they choose that number and not a lower one ☐ **yes** ☐ **no**

If patient says:
- ≥ 2, ask *"Why did you choose that number and not a lower one?"* (i.e., *"What are some reasons you would engage in treatment?" "What else?"*

This is intended to pull for change talk, a patient's own argument for change. The change talk is then reflected. See below.

If the patient's response is 1 or unwilling, ask *"What would it take for that "1" turn into a "2"?* **Or** *"Imagine you did go to treatment; how could that be helpful to you?"* **Or**

"What would have to happen for you to be ready? "How important would it be for you to prevent that from happening?"

Reflect/reiterate positive reasons for change.

Discuss pros and cons of treatment. (See Appendix 3 on pages 48-49 in manual)

Appendix 7: BNI+ MAUD Adherence & Competence Checklist *(continued)*

10. Reflect patients' statements regarding change ☐ yes ☐ no

Then REFLECT on their reasons and reinforce with clinical information (e.g., *"Treatment does in fact work to help reduce and eliminate use, as well as the problems connected with it."*

Take the participant's answer from the above question and ask: *"What makes that reason important to you?"*

11. Elicit response (How does all this sound to you?) ☐ yes ☐ no

Reiterate what participant says in above and say, *"What's the next step?"* [PAUSE] *"Are you accepting the referral?"*

12. Negotiate the goal (What would you like to do?) ☐ yes ☐ no

IF *"YES"* to treatment, skip to bottom "Secure Referral".

IF *"NO,"* reiterate reasons participant gave above and <PAUSE>.

IF STILL *"NO,"* then ask if the participant might consider it and give advice below for patient to have, even if they do not want to consider it, and then SKIP TO "Provide Handouts".

13. Give advice if necessary ☐ yes ☐ no

Based on the response above, follow with: *"If you leave the hospital with a plan for continued treatment for your alcohol use, you will be on the road to better health.* (see facilitated referral next page.) *Based on what you told me and what we know about alcohol use disorder, I think you should...", "it may be a good idea to address this now..."*

"Remember what we discussed in terms of improving your health."

Once a *"YES"* has been secured, follow with: *"Which of these options might you be interested in?"* [SHOW LIST]

14. Summarize (This is what I've heard you say...) ☐ yes ☐ no

15. Have patient fill out agreement card ☐ yes ☐ no

"Let's complete this referral agreement together which will reinforce your decision to seek treatment. This is really an agreement between you and yourself."

"Ok, based on your preferences, your insurance information and availability of programs, I can make a few phone calls to schedule an appointment for you. You may even be able to speak to someone while you are here in the hospital."

"Once I make a few calls, I will come back and we can update the referral agreement. How does that sound?"

Appendix 7: BNI+ MAUD Adherence & Competence Checklist *(continued)*

16. Upon return, review information with the patient and update the referral agreement

☐ yes ☐ no

Provide summary
Update form with appointment date and time
Ask if they have any questions: *"What questions do you have?"*

17. Provide trifold ☐ yes ☐ no

Hand the participant the trifold.

18. Introduce MAUD ☐ yes ☐ no

Once the BNI is complete, follow with the next statement.

"As part of the plan for addressing your alcohol use, I would like to talk to you about medications that can help reduce cravings. Is it okay if I discuss that with you?"

If they say *"YES,"* move on to the next statement.

If they say *"NO,"* reiterate reasons participant gave above about wanting to reduce alcohol use.

"Like medications for high blood pressure or diabetes, these medications can be prescribed by any doctor. Medications can be used in combination with other treatments like AA and can also be used without other treatments."

Introduce the trifold

"There are three main medications available to reduce alcohol use: naltrexone, acamprosate, and disulfiram. These medications come as a pill form to be taken once a day, like other medications, and one of them – naltrexone – also called Vivitrol – is available as a monthly injection. There are other medications such as gabapentin and topiramate that are sometimes used as well."

"Have you ever received any of these medications?" If "YES", which medications?"

"Some medications (except disulfiram [also known as Antabuse]) can be started or continued while drinking alcohol."

"These medications can be very helpful for decreasing cravings to drink alcohol and are very safe."

Reflect participants statement and move on to the next statement.

"Before I go, I would like you to summarize what we discussed... Finally, based on my understanding you are interested in finding out more about (insert name of medication) *and will discuss options further with your primary care team."*

Appendix 7: BNI+ MAUD Adherence & Competence Checklist *(continued)*

19. Update the agreement form and thank the participant for their time ☐ **yes** ☐ **no**

Comments: ____

____

____

____

____

Start time: ____________________ End time: ____________________

Appendix 7: BNI – MAUD – CBT4CBT Checklists

BNI+ MAUD+ CBT4CBT Adherence & Competence Checklist

1. Ask the patient for permission to discuss alcohol use ☐ yes ☐ no

HOW TO START CONVERSATION:

"May we spend a few minutes talking about your use of alcohol." <PAUSE>

If the patient response is *"YES,"* use the following statement.

"I want to talk about how it's affecting you and how we might be able to help."

Follow the statement with the next questions.

"Tell me how you are feeling right now? When is the last time you drank alcohol?"

If the patient response is *"NO"* use the following statement.

"When would be a great time for us to discuss your alcohol related concerns?"

Or

"We don't have to talk about your alcohol use, we can talk about other concerns that you may have."

*Make sure to **avoid a non-judgmental stance**, acknowledge the situation, be mindful of cultural values for the racial and ethnic minoritized individual, and focus on engagement. Reflective listening is KEY.

2. Review patient's drinking patterns ☐ yes ☐ no

Reflect patients last statement and follow with the next comment.

"From what I understand you are drinking (fill in amounts from screening)."
BUT DO NOT RE-SCREEN.

Follow the statement above with one of the next questions.

"How concerned are you with your alcohol use, if at all?" **Or** "*What concerns has it caused you, if any?"*
Or *"How concerned or worried is your family with your drinking?"* <PAUSE>

Make sure to reflect the patient's statement. For example, "*it sounds like there is some concern with your alcohol use."*

3. Express concern about these patterns ☐ yes ☐ no

Reflect the participant's statement above and assess alcohol use and medical illness connection with the following question:

Appendix 7: BNI+ MAUD+ CBT4CBT Adherence & Competence Checklist *(continued)*

"What connections might you see between your use of alcohol and your medical condition?"

Reflect the participants statement and follow it up with the next question.

"Has there been a time in your life when you stopped drinking or cut back in the past?"

"If 'YES,' please tell me about how you did it?"

"If 'NO,' tell me what has been helpful in your life in overcoming obstacles? **Or** *"In the past, how have you managed a difficult situation."*

IF PATIENT STATES ANY ACCURATE & RELEVANT CONNECTION(S),
reiterate them, acknowledge (affirm) the patient's good insight, and give additional relevant facts on medical risks related to alcohol use.

IF PATIENT DOES NOT STATE ANY ACCURATE & RELEVANT CONNECTIONS,
MAKE the connection based on general caution regarding alcohol use.

"From research we know that any alcohol use compromises decision making and health behaviors. In addition, we know that any alcohol use can be harmful to health."

4. Ask about a connection ☐ **yes** ☐ **no**

Reflect participant's comment above and follow with the next question if participant did not already acknowledge a connection between alcohol and this hospitalization.

"What (if any) connection is there between your alcohol use and this hospitalization?" <PAUSE>

See next steps below.

5. Reflect the patient's statement ☐ **yes** ☐ **no**

IF PARTICIPANT SEES ANY ACCURATE & RELEVANT CONNECTION, reiterate (reflect) what they have said.

6. Provide medical facts and information that there is a connection between the hospitalization and drinking ☐ **yes** ☐ **no**

Based on the information obtained above, follow with one of the options below.

SAMPLE: *"You're right,* [reiterate patient's connection] *... that's a good connection to make."*

IF PATIENT DOES NOT SEE AN ACCURATE CONNECTION, then make one using facts (if applicable).

This is a key step component of respecting autonomy: ***Ask for permission to share: Is it okay if I share an observation? If yes, follow the sample on next page.***

Appendix 7: BNI+ MAUD+ CBT4CBT Adherence & Competence Checklist *(continued)*

SAMPLE: *"One of the things that we see as a connection is that your patterns of alcohol use may have contributed to your fall, physical discomfort, or whatever they're in the hospital for."* (if relevant)

Follow the statement with: *"What do you make of that?"*

IF THERE ACTUALLY IS NO CONNECTION between the hospitalization and alcohol use, ask about any potential connections between alcohol use and overall health issues.

7. Inform patient of NIAAA guidelines by using the pamphlet or card ☐ yes ☐ no

Hand patient trifold Project ENHANCE BNI Trifold.pdf

Whether a connection between hospitalization and drinking has been made, the next step is to provide information regarding the spectrum of alcohol use and recommendations regarding safer drinking limits; introduce relevant content on the BNI trifold.

Ask for permission to share information on NIAAA guidelines: *"Is it okay if I review with you what is considered safer drinking limits for your age and gender?"*

If the participant's response is *"YES"* proceed with sharing information. If they say *"NO",* say – "*tell me what you usually drink."*

If the participant's response was *"YES"* and drinking norms have been shared ask the following question: *"What do you make of the information just shared."*

This is where we want to bring it all together and use a summary. For example, *"Based on what we discussed so far, there seems to be some concern with your drinking and there is an indirect connection between your alcohol use and current hospitalization. Did I get that correct?"*

If the participant's response is *"YES,"* move on to the following statement.

"So, in order to help you with all of this, I would like to talk with you a bit further about how we might be able to help you reduce the concerns associated with your alcohol use. For example, I want to encourage you to consider that there are a wide variety of treatment options available and that I am prepared to go over them with you." (See and show treatment referral list.)

Ask for permission to share treatment options: *"Is it okay if I share some options with you?"*

If *"YES,"* move forward with discussion of treatment options (reference trifold).

Explain different types of treatment options available

Medically Supervised Withdrawal Management (traditionally known as "Detox")
Partial Hospitalization Program (PHP)

Appendix 7: BNI+ MAUD+ CBT4CBT Adherence & Competence Checklist *(continued)*

Intensive Outpatient Program (IOP)
AA (Alcoholics Anonymous) and Narcotics Anonymous (NA)

To explore more positive thoughts about treatment ask *"Why might you decide to go? Have you thought about going before? Why might it be important to seek treatment now? What were some of the reasons why? Why is it important to have this conversation now?"*

"Would you mind If I gave you some possible reasons?"

If patient says, *"YES,"* then reflect on the possible consequences of treatment.

If patient says, *"NO,"* then discuss possible benefits of treatment (e.g., reversing the negative effects of physical alcohol dependence discussed in Steps 1 & 2).

Offer summary reflection of patient's reasons for starting or considering treatment, ending with a reinforcement of the patient's autonomy… *"Ultimately, the decision to seek treatment is up to you."*

Close with: *"If you decide to accept our recommendation, you would be taking the first step towards addressing the problems connected with your alcohol use that we've been discussing."*

8. Ask patient to identify readiness to change on Readiness Ruler ☐ **yes** ☐ **no**

Show card

Start with: *"Now that we have discussed treatment options, on a scale from 1-10, where 1 is 'not at all' and 10 is 'right now', how ready are you to engage in alcohol treatment after your hospitalization?"*

9. Ask why they choose that number and not a lower one ☐ **yes** ☐ **no**

If patient says:
- ≥ 2, ask *"Why did you choose that number and not a lower one?"* (i.e., *"What are some reasons you would engage in treatment?" "What else?"*

This is intended to pull for change talk, a patient's own argument for change. The change talk is then reflected. See below.

If the patient's response is 1 or unwilling, ask *"What would it take for that "1" turn into a "2"?* **Or** *"Imagine you did go to treatment; how could that be helpful to you?"* **Or**

"What would have to happen for you to be ready? "How important would it be for you to prevent that from happening?"

Reflect/reiterate positive reasons for change.

Discuss pros and cons of treatment. (See Appendix 3 on pages 48-49 in manual)

Appendix 7: BNI+ MAUD+ CBT4CBT Adherence & Competence Checklist *(continued)*

10. Reflect patients' statements regarding change ☐ yes ☐ no

Then REFLECT on their reasons and reinforce with clinical information (e.g., *"Treatment does in fact work to help reduce and eliminate use, as well as the problems connected with it."*

Take the participant's answer from the above question and ask: *"What makes that reason important to you?"*

11. Elicit response (How does all this sound to you?) ☐ yes ☐ no

Reiterate what participant says in above and say, *"What's the next step?"* [PAUSE] *"Are you accepting the referral?"*

12. Negotiate the goal (What would you like to do?) ☐ yes ☐ no

IF *"YES"* to treatment, skip to bottom "Secure Referral".

IF *"NO,"* reiterate reasons participant gave above and <PAUSE>.

IF STILL *"NO,"* then ask if the participant might consider it and give advice below for patient to have, even if they do not want to consider it, and then SKIP TO "Provide Handouts".

13. Give advice if necessary ☐ yes ☐ no

Based on the response above, follow with: *"If you leave the hospital with a plan for continued treatment for your alcohol use, you will be on the road to better health.* (see facilitated referral next page.) *Based on what you told me and what we know about alcohol use disorder, I think you should...", "it may be a good idea to address this now..."*

"Remember what we discussed in terms of improving your health."

Once a *"YES"* has been secured, follow with: *"Which of these options might you be interested in?"* [SHOW LIST]

14. Summarize (This is what I've heard you say...) ☐ yes ☐ no

15. Have patient fill out agreement card ☐ yes ☐ no

"Let's complete this referral agreement together which will reinforce your decision to seek treatment. This is really an agreement between you and yourself."

"Ok, based on your preferences, your insurance information and availability of programs, I can make a few phone calls to schedule an appointment for you. You may even be able to speak to someone while you are here in the hospital."

"Once I make a few calls, I will come back and we can update the referral agreement. How does that sound?"

Appendix 7: BNI+ MAUD+ CBT4CBT Adherence & Competence Checklist *(continued)*

16. Upon return, review information with the patient and update the referral agreement

☐ yes ☐ no

Provide summary
Update form with appointment date and time
Ask if they have any questions: *"What questions do you have?"*

17. Provide trifold ☐ yes ☐ no

Hand the participant the trifold.

18. Introduce MAUD ☐ yes ☐ no

Once the BNI is complete, follow with the next statement.

"As part of the plan for addressing your alcohol use, I would like to talk to you about medications that can help reduce cravings. Is it okay if I discuss that with you?"

If they say *"YES,"* move on to the next statement.

If they say *"NO,"* reiterate reasons participant gave above about wanting to reduce alcohol use.

"Like medications for high blood pressure or diabetes, these medications can be prescribed by any doctor. Medications can be used in combination with other treatments like AA and can also be used without other treatments."

Introduce the trifold

"There are three main medications available to reduce alcohol use: naltrexone, acamprosate, and disulfiram. These medications come as a pill form to be taken once a day, like other medications, and one of them – naltrexone – also called Vivitrol – is available as a monthly injection. There are other medications such as gabapentin and topiramate that are sometimes used as well."

"Have you ever received any of these medications?" If "YES", which medications?"

"Some medications (except disulfiram [also known as Antabuse]) can be started or continued while drinking alcohol."

"These medications can be very helpful for decreasing cravings to drink alcohol and are very safe."

Reflect participants statement and move on to the next statement.

"Before I go, I would like you to summarize what we discussed… Finally, based on my understanding you are interested in finding out more about (insert name of medication) *and will discuss options further with your primary care team."*

Appendix 7: BNI+ MAUD+ CBT4CBT Adherence & Competence Checklist *(continued)*

19. Start of CBT4CBT ☐ yes ☐ no

Script for HPA to describe CBT4CBT

"As part of your participation in this research study, you have been assigned to receive a computerized version of cognitive behavioral therapy, called CBT4CBT. I'd like to spend a few minutes telling you about it."

Provide CBT4CBT Trifold

POINT TO SECTION OF TRIFOLD: "What is cognitive behavioral therapy?"

"The CBT4CBT program is designed to teach you skills and strategies to help you reduce or stop drinking. It is based on a type of talk therapy, called cognitive behavioral therapy, or CBT, which is one of the most effective treatments for those wanting to change their alcohol use. Rather than a counselor teaching you the skills in CBT, you will learn them on your own through this interactive computer program."

POINT TO SECTION OF TRIFOLD: "CBT4CBT will teach you how to…"

"The CBT4CBT program will help you learn how to recognize, avoid, and cope with your triggers and things that set you up to drink. There are 7 total lessons in the program, and each one takes about 35-40 minutes to complete. But you'll be able to go at your own pace and can stop and restart a lesson whenever you like. The program has a narrator who will guide you through the lessons. Each lesson includes some brief movies and interactive exercises to help you learn skills to address your alcohol use."

"The CBT4CBT program has been used in many prior studies and is effective at helping people reduce or stop their alcohol or drug use. It is completely safe and confidential. You will not be entering in any personal information."

Important information to convey

- The great thing about this program is that you can do it whenever and wherever you want. You don't have to make an appointment or talk to anyone.
- We recommend that you access the program while in a quiet and private space. Think of this like a therapy session - you want to make sure you can give it your full attention without distractions.
- You will need to hear the narrator and characters in the program, so try to find a quiet space if possible. Headphones could be helpful.
- There are a lot of videos in the program, so we recommend using a device with a large screen (10 inches or greater). A tablet or laptop is recommended for optimal viewing. You can use your phone to access the program, but you may need to scroll a bit to see everything on the screen.

Appendix 7: BNI+ MAUD+ CBT4CBT Adherence & Competence Checklist *(continued)*

- We ask that you complete at least one CBT4CBT lesson while you're here in the hospital. After you leave the hospital, we recommend you complete 1 or 2 lessons per week. But you are free to access it as often as you like.

"Before I show you how to access it, do you have any questions?"

Script and Procedure for HPA to help patient log in to Computer-Based Training for Cognitive Behavioral Therapy

POINT TO SECTION OF TRIFOLD "HOW DO I ACCESS IT?"

"To access the program, you'll go to the following website – home.cbt4cbt.com"

"Or you can scan the QR code here on the pamphlet using your camera"

"Although the program can be accessed on a phone, the best way to view it is on a screen larger than 10 inches. So, we highly recommend using a tablet or laptop. But if you're more comfortable on your phone, that's OK too."

HELP PATIENT ENTER IN WEB ADDRESS ON THEIR DEVICE

SHOW THEM HOW TO SCAN QR CODE USING THEIR CAMERA

"Here is the main screen where you will log in to the program"

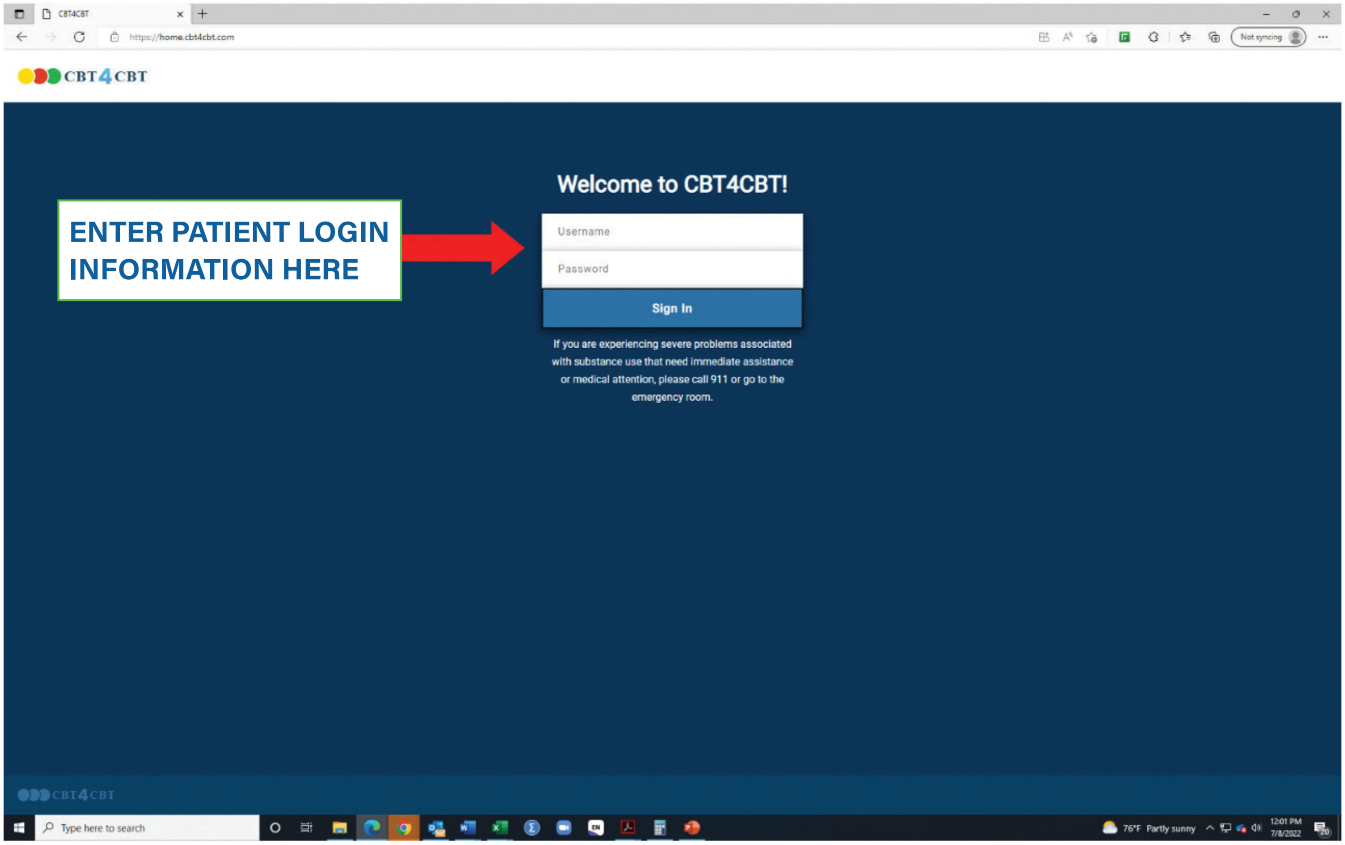

Appendix 7: BNI+ MAUD+ CBT4CBT Adherence & Competence Checklist *(continued)*

“Let’s enter your login information.”

“Please type in the following - Your username is...”

“Your password is...”

“You’ll need this information each time you log in. Let’s write these down so you can remember them. You might also want to keep it somewhere in your phone so you can access it if you forget.”

HELP PATIENT WRITE DOWN USERNAME AND PASSWORD. SHOW THEM HOW TO PUT IT IN SMARTPHONE ‘NOTE’ FUNCTION IF NEEDED

“If you ever forget your log in information, you can call or text our study coordinator at xxx-xxx-xxxx.”

PROVIDE STUDY COORDINATOR CONTACT INFO IF PATIENT DOES NOT HAVE

“Once you enter the login information, click/touch SIGN IN.”

HAVE PATIENT CLICK/TOUCH SIGN IN

“This is the home page of the program.”

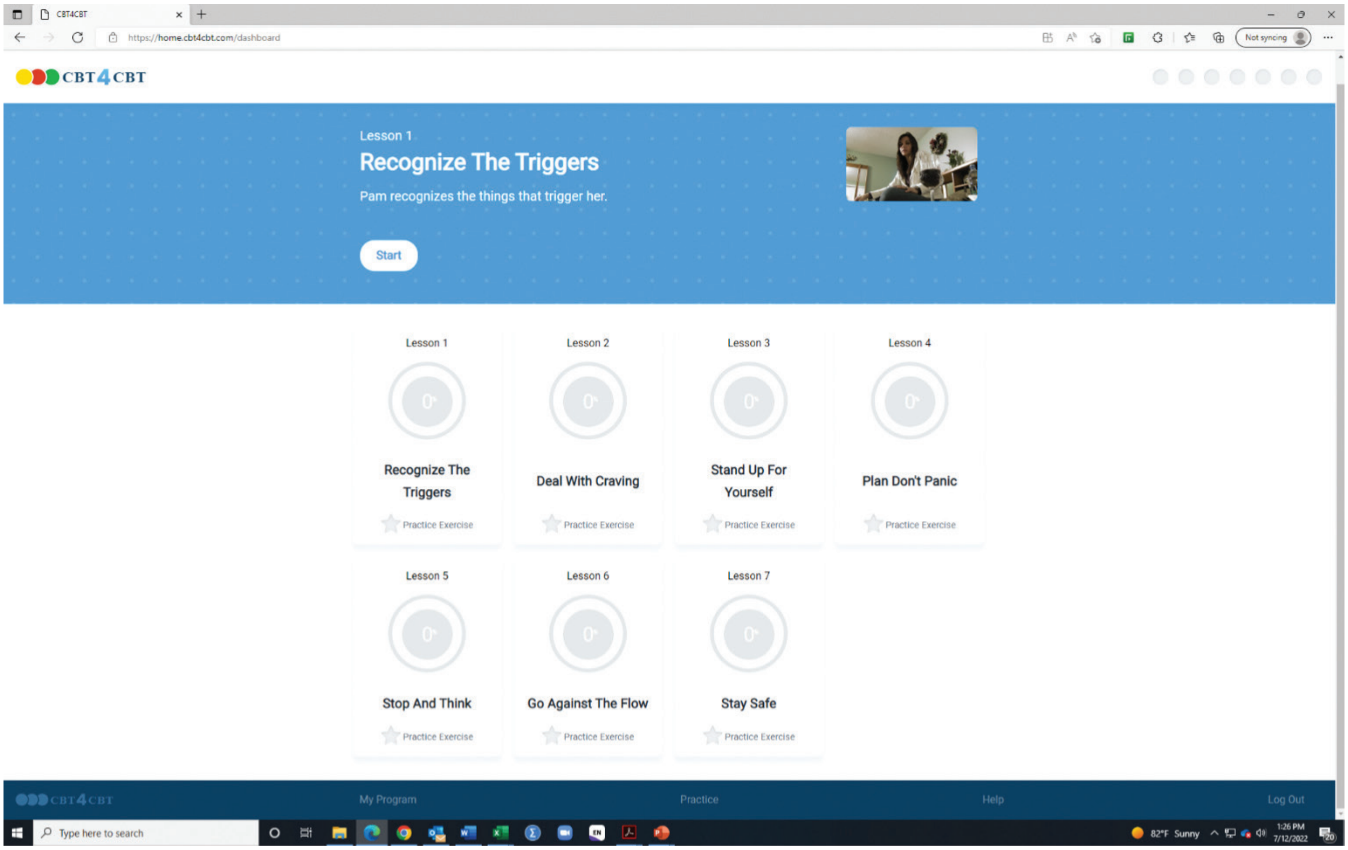

Appendix 7: BNI+ MAUD+ CBT4CBT Adherence & Competence Checklist *(continued)*

"You'll see the 7 lessons here on the home page. The first lesson is highlighted at the top of the page, it's called Recognize the Triggers. Before you start the lesson, let me just show you around the home page."

"Here are the 7 lessons. They each have a grey circle that keeps track of your progress in each lesson. Right now, they're all at 0%."

POINT TO THE PROGRESS CIRCLE FOR EACH LESSON

"When you complete a lesson, the circle will fill up and you'll receive a badge for completion. If you leave a lesson before completing it, the circle will show you what percentage you've completed."

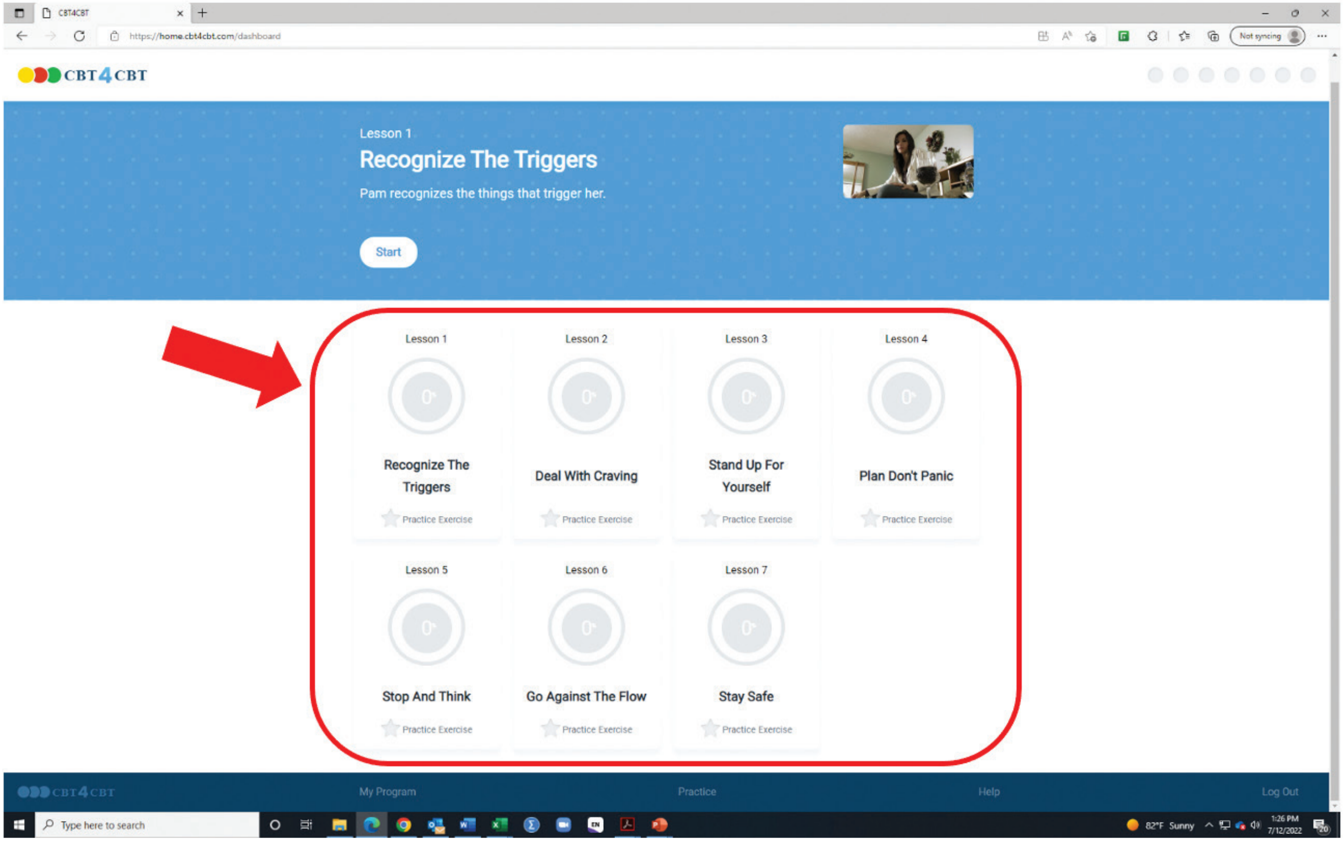

Appendix 7: BNI+ MAUD+ CBT4CBT Adherence & Competence Checklist *(continued)*

"Under the name of each lesson, there's a small star with the words PRACTICE EXERCISE."

POINT TO THE PRACTICE EXERCISE STAR

"This star will light up once you complete a practice exercise for that lesson. The practice exercises are a very important part of the program. The more you practice these skills, the better you'll do."

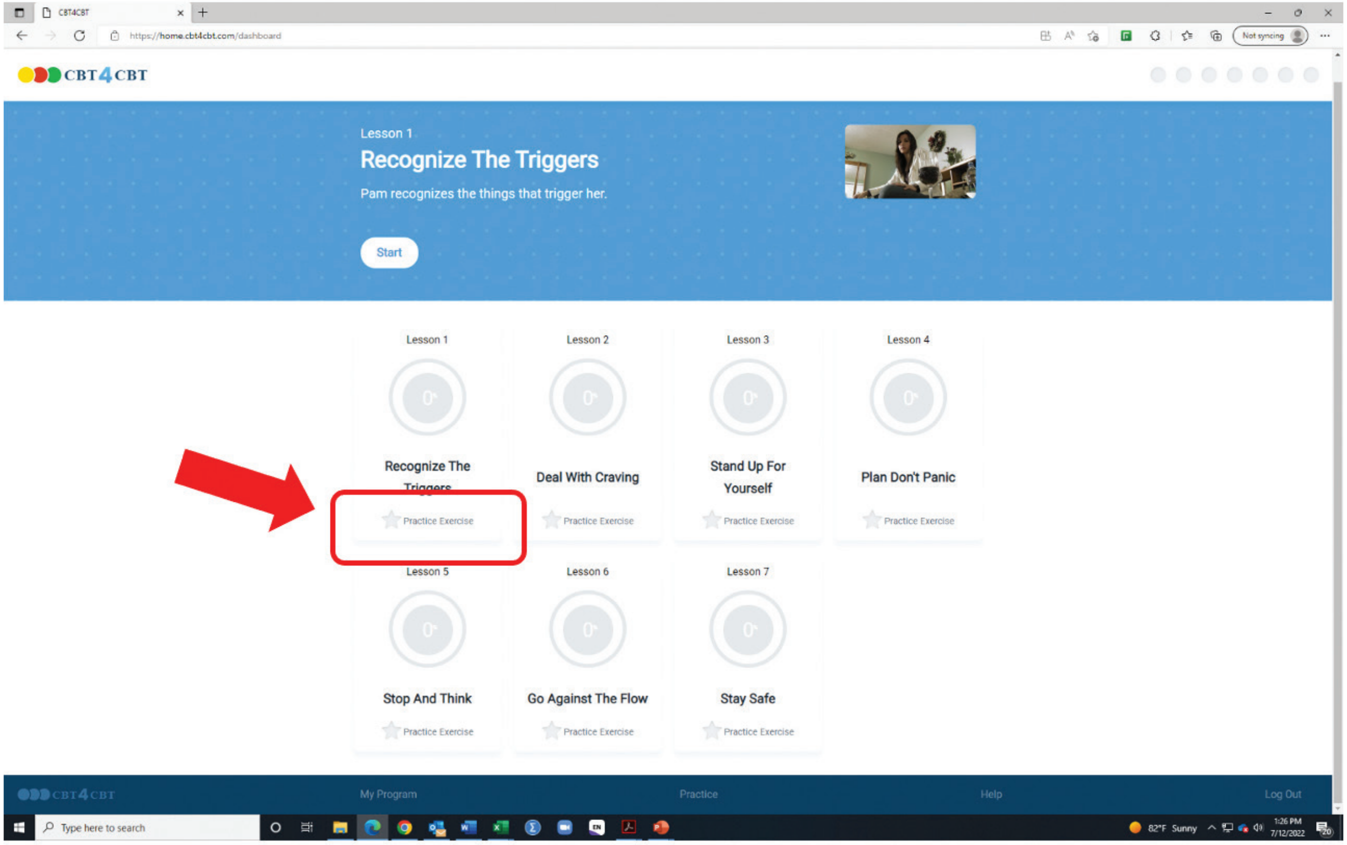

Appendix 7: BNI+ MAUD+ CBT4CBT Adherence & Competence Checklist *(continued)*

"Along the bottom of the page, there are 4 different tabs that will take you to different areas of the program."

POINT TO THE TABS AT THE BOTTOM OF THE PAGE

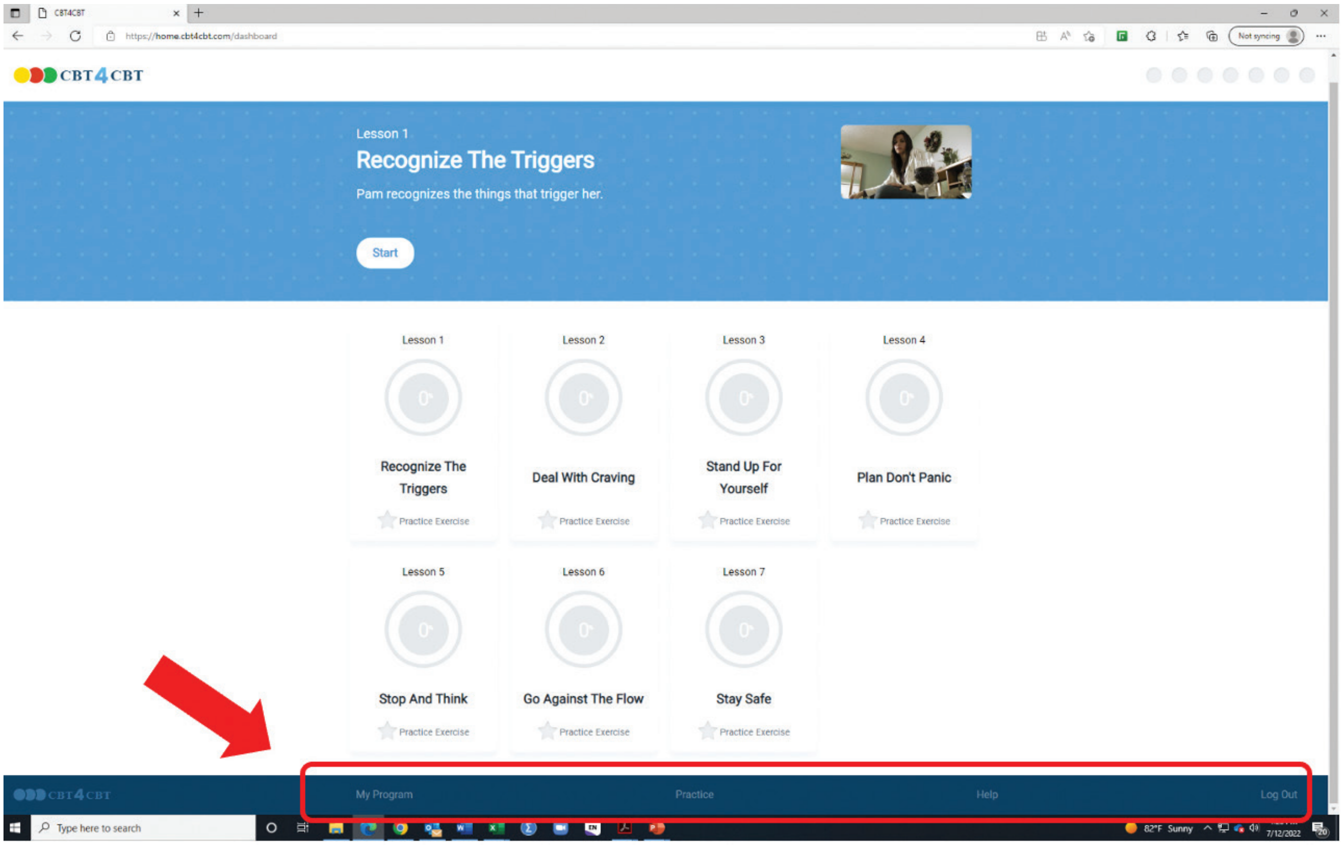

"The tab called MY PROGRAM will take you this home page with all the lessons. When you are in one of the lessons and want to get back to this home page, just click/touch the MY PROGRAM tab at the bottom."

"The tab called PRACTICE will take you to a page that has all of the practice activities within each lesson."

Appendix 7: BNI+ MAUD+ CBT4CBT Adherence & Competence Checklist *(continued)*

HAVE PATIENT CLICK/TOUCH PRACTICE TAB TO SHOW THEM WHERE IT LEADS

"This is the Practice page. These are all blank right now because you haven't done any practice activities yet. In each lesson, you'll be asked to complete practice activities on the screen. You'll be able to download blank practice worksheets here on this page whenever you want to practice a skill."

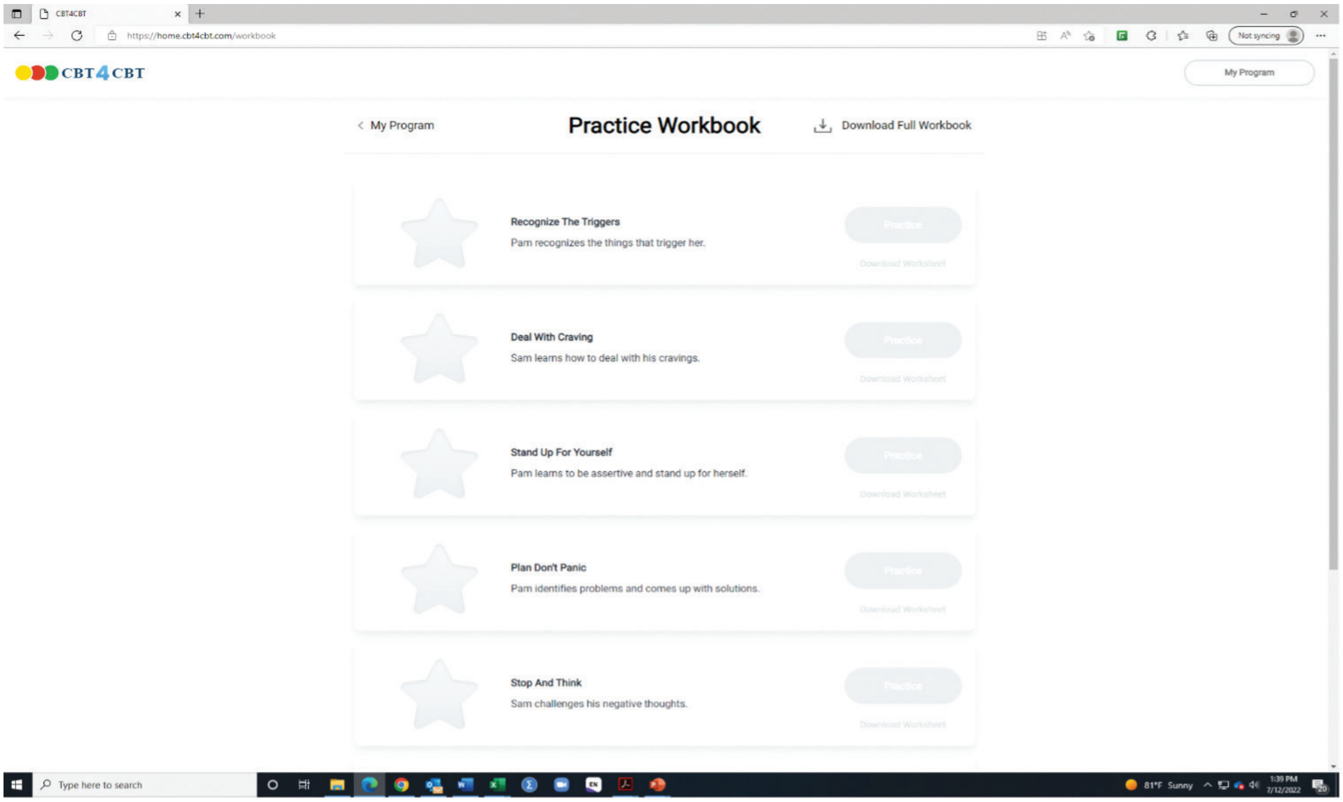

Appendix 7: BNI+ MAUD+ CBT4CBT Adherence & Competence Checklist *(continued)*

“Please scroll down on the PRACTICE page to see the tabs at the bottom of the page. Touch/click the MY PROGRAM button to go back to the home page.”

HAVE PATIENT CLICK/TOUCH MY PROGRAM TAB

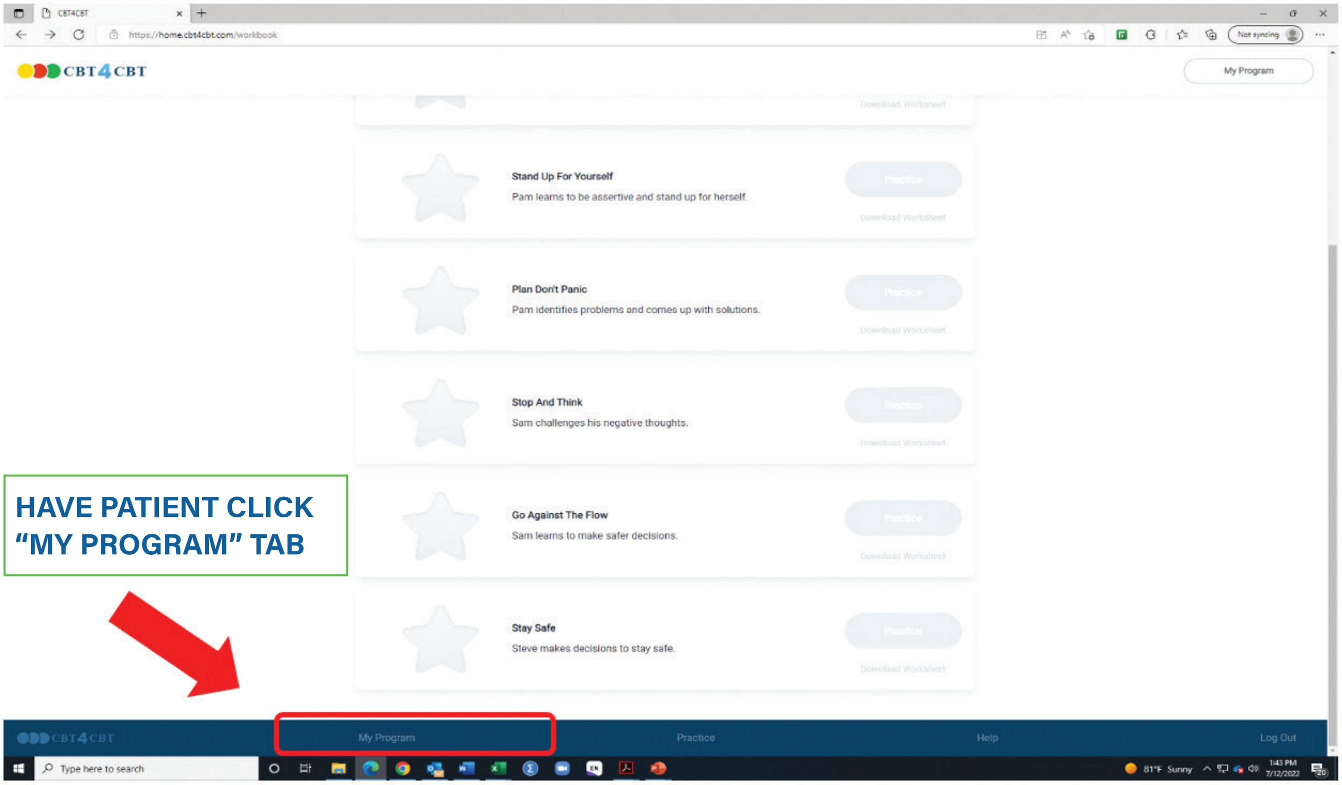

Appendix 7: BNI+ MAUD+ CBT4CBT Adherence & Competence Checklist *(continued)*

"Now we're back on the home page with all the lessons. The LOG OUT tab at the bottom of the screen will log you out of the program. Whenever you click/touch that tab, you'll be logged out and will go back to the login screen to enter your information again."

"The HELP tab on the bottom will bring you to a brief video to test the sound volume. Otherwise if you're having trouble with the program, you'll need to contact the research coordinator at xxx-xxx-xxxx."

"OK, when you're ready to start the lesson, just click/touch the start button at the top. This will take you to the first lesson, where a narrator will appear and will guide you through the lesson. Again, at any point, if you want to come back to this home page, just click/touch the MY PROGRAM tab at the bottom."

HAVE PATIENT CLICK/TOUCH THE START BUTTON

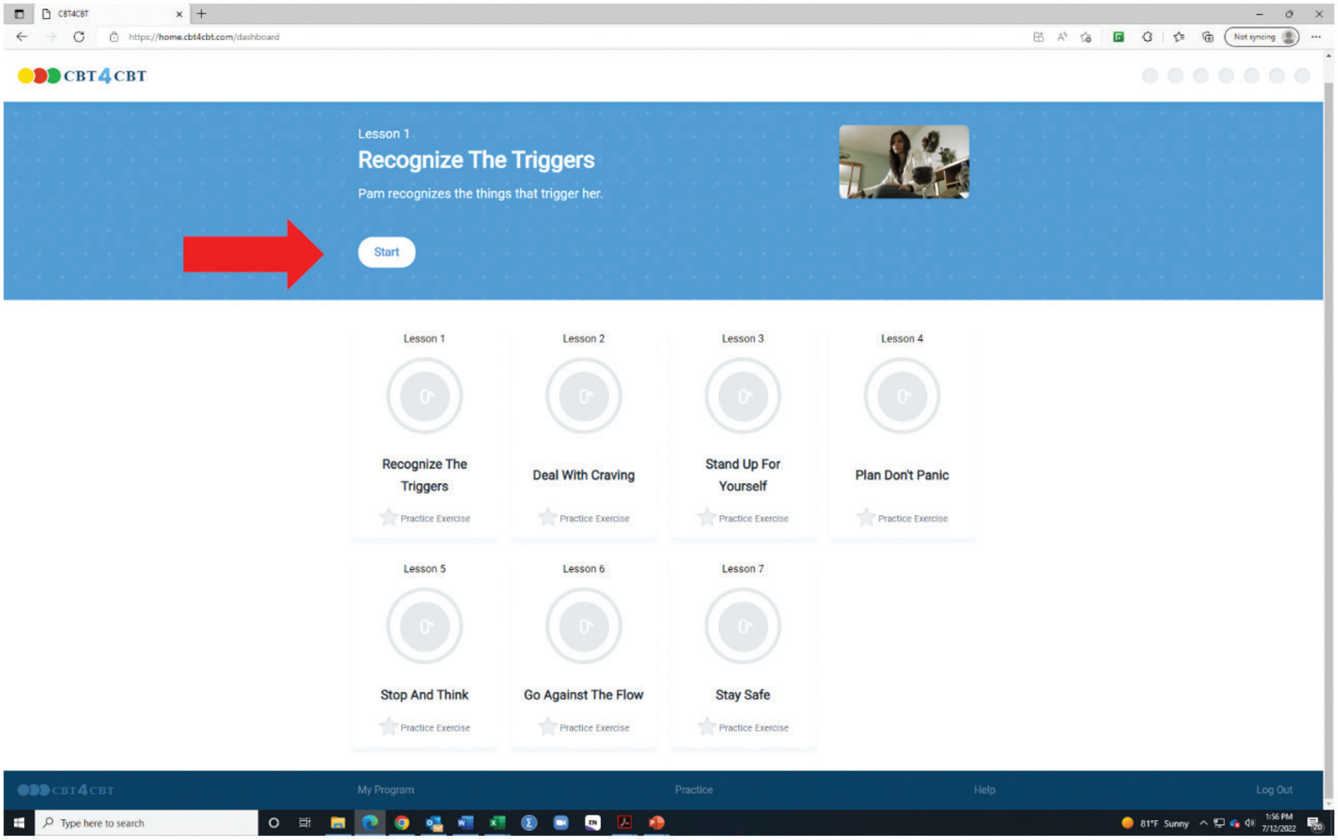

Appendix 7: BNI+ MAUD+ CBT4CBT Adherence & Competence Checklist *(continued)*

THE NARRATOR WILL APPEAR AND PROVIDE AN INTRODUCTION

SIT WITH PATIENT WHILE THEY WATCH THE 54s INTRODUCTION

"As the narrator said, please click/touch the forward arrow to continue." Do you see it on the screen? This is how you'll move forward or backward through each lesson. You will click on NEXT to move to the next screen. Or you can click on BACK to go back to a prior screen in the lesson."

"Do you have any questions?"

"When you're ready, click/touch the NEXT button to move forward with the lesson. I will leave you to finish the rest of the lesson on your own."

"Please try to complete at least one lesson before you leave the hospital."

IF PATIENT DOESN'T CLICK/TOUCH THE NEXT BUTTON, SHOW THEM WHERE TO CLICK/ TOUCH NEXT TO MOVE ONTO THE NEXT SCREEN

Project ENHANCE CBT4CBT trifold_English.pdf

Project ENHANCE CBT4CBT trifold_Spanish.pdf

20. SUMMARY OF STEP 4 BNI+ Facilitated Provision of MAUD+ CBT4CBT

In Step 4 of the BNI+ Facilitated Provision of MAUD+ CBT4CBT, the HPA assists the patient in exploring a menu of options regarding reducing their alcohol use and engaging in treatment in addition to exploring MAUD, as well as introducing CBT4CBT. The HPA also provides instructions on how to access CBT4CBT and helps them log in for the first time. The HPA encourages the patient to complete the first session at that time or gets a commitment from the patient to complete a session prior to hospital discharge.

Step 4 is ended by scheduling a 2-week out booster session and asking the patient if they have any questions about the information or instructions and by thanking them for their time.

Thank the participant for their time ☐ **yes** ☐ **no**

Comments: ____

____

____

____

____

Start time: ____________________ End time: ____________________

Appendix 8: BNI Trifold - English

What is Alcohol Use Disorder?

Similar to conditions such as diabetes or high blood pressure, alcohol use disorder (AUD) is a chronic medical condition. When someone has an alcohol use disorder, they:

1. experience loss of control of alcohol use;
2. drink alcohol despite adverse consequences;
3. crave alcohol.

Treatments are available, including counseling and medication options. Talk to your health care clinician to learn more about your options.

What is the spectrum of alcohol use?

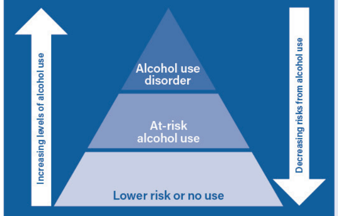

Reducing your alcohol use, or avoiding alcohol completely, will lead to better overall health and other improvements in your life.

Treatment Resources for Individuals with Substance Use and Mental Health Disorders

APT Foundation-Residential Services – 203-337-9943, 44-54 East Ramsdell St., New Haven (co-ed). Offers residential services for adults, including Spanish-speakers, HIV+ individuals, and those with primary mental health disorders.

Aware Recovery Care – 203-631-2556. Offers in-home addiction treatment, medically managed withdrawal, MAT, recovery coaching, psychotherapy, care coordination/case management, and family wellness/education.

CASA – 203-339-4112, Bridgeport (co-ed). Offers intensive and intermediate residential services, OP, IOP, and partial hospitalization programs (PHP) for Spanish-speaking individuals.

CHR-Milestone – 860-928-1860, Option #1, Putnam (female only). Offers dual-diagnosis intensive residential services.

CHR-Roots to Recovery – 860-456-1769, Option #5, Willimantic (male only). Offers long-term/intermediate residential services.

Connecticut Renaissance-McAuliffe Center – 203-346-1931, Waterbury (men only). Offers dual-diagnosis residential services.

Connecticut Valley Hospital-Blue Hills – 860-293-6400, Hartford (co-ed). Offers medically managed withdrawal and residential services.

Connecticut Valley Hospital-Merritt Hall – 800-828-3396, x5, Middletown (co-ed). Offers medically managed withdrawal and residential services.

First Step – 203-416-1915, Bridgeport (co-ed). Offers medically monitored withdrawal for adults.

Grant Street Partnership – 203-503-3000, 60-62 Grant St., New Haven (men only). Offers IOP/OP services (18+).

High Watch Recovery Center – 860-927-3772, 62 Carter Rd., Kent. Offers medically managed withdrawal, residential services, extended care sober living, and virtual IOP.

InterCommunity Recovery Centers, Inc. – 860-569-5900, Hartford (co-ed). Medically monitored withdrawal: x515 | Residential Rehab: x541. Offers medically monitored withdrawal, OP, IOP, medication treatment, and intensive and intermediate residential services.

Liberation Programs – 855-542-7764, Bridgeport (co-ed). Offers long-term/intermediate residential services, medically monitored withdrawal, MAT (Suboxone/ Naltrexone/ Methadone), IOP, and OP treatment.

Midwestern CT Council of Alcoholism (MCCA) – 203-792-4515, Danbury (co-ed). Offers intensive and intermediate residential services, medically monitored withdrawal, MAT (Suboxone/ Naltrexone), IOP, and OP treatment.

Mountainside – 800-762-5433, x111, Canaan. Offers medically monitored withdrawal, residential services, extended care sober living, OP, LGBTQ Group, and MAT (Suboxone/Vivitrol).

Regional Network of Programs (RNP)-Horizons – 203-333-3518, Bridgeport (co-ed). Offers intensive residential services.

Regional Network of Programs (RNP)-New Prospects – 203-610-6252, Bridgeport (co-ed). Offers dual-diagnosis residential services.

Retreat Behavioral Health – 203-873-9768, New Haven (co-ed). Offers medically monitored withdrawal, residential services, MAT (Suboxone/Vivitrol), OP, and IOP.

Rushford Treatment Center – 877-577-3233, multiple locations (co-ed). Offers medically monitored withdrawal, residential services, MAT (Suboxone), PHP and OP for adults and adolescents.

SCADD – 860-447-1717, New London (co-ed) | Lebanon (men only). Offers medically monitored withdrawal and long-term dual diagnosis residential services.

Silver Hill Hospital – 866-542-4455, New Canaan, (co-ed). Offers medically monitored withdrawal and residential services for adolescents and adults.

Stonington Institute – 800-832-1022, North Stonington (co-ed). Offers medically monitored withdrawal, residential services, dual-diagnosis PHP with a sober living residence and IOP.

Yale IRB #2000031874

Promoting Alcohol Treatment Engagement Post-Hospitalization

Project ENHANCE

ENhancing Hospital-initiated Alcohol TreatmeNt to InCrease Engagement

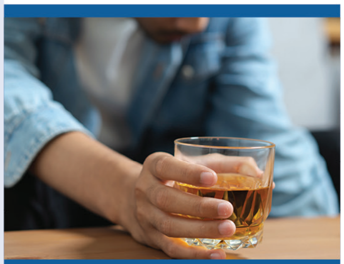

What is Alcohol Use Disorder?

Yale

Project ENHANCE

What is a standard drink?

One standard drink is defined as:

12 fl oz of Regular Beer	8–9 fl oz of Malt Liquor	5 fl oz of Table Wine	1.5 fl oz shot of 80-proof Distilled Spirits (gin, rum, tequila, vodka, whiskey, etc.)
5% alcohol	7% alcohol	12% alcohol	40% alcohol

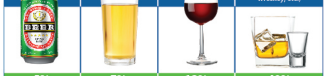

What are the standard drinking limits for adults?

Maximum drinks per week/occasion

	Single-day Limit	Weekly Limit
Men age 65 or under	4 or less standard-size drinks	14 or less standard-size drinks
Women or men age 65 or over	3 or less standard-size drinks	7 or less standard-size drinks

What are potentials problems from alcohol use?

These are some of the problems that can happen with risky drinking:

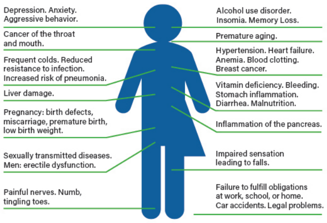

Programs Offering Medication Treatment

APT Foundation – 203-781-4600, 1 Long Wharf Dr., Ste. 10, New Haven. Offers OP treatment including individual, groups, IOP, and medication services. Spanish-speakers and HIV+ individuals serviced. No appointment necessary, walk-in screenings available Monday-Friday 8:30-11am.

Bridges Healthcare, Inc. – 203-878-6365, 949 Bridgeport Ave., Milford. Dual diagnosis OP program. Offers day and evening groups, IOP, and medication. Accepts Medicaid, Medicare, and BCBS.

Bridges Healthcare: Mobile Addiction Treatment Team (MATT's van) – 203-494-5811. Offers medication and recovery coaching services for adults 18+. No insurance required. Schedule/hours varies per locations.

CSHHC - Addiction Recovery Clinic – 203-503-3000, 150 Sargent Dr., New Haven. Offers medication and outpatient treatment. Limited hours: Tuesday 8am-12pm, Friday 1-5pm.

Fair Haven Community Health Clinic – 203-809-3511, 374 Grand Ave., New Haven. FHCHC is an integrated program that offers both medical and behavioral health care for adults 18+, LGBTQ+, and HIV+. Services are bilingual. Provides medications and behavioral treatment for substance use disorder.

Liberation Programs, Inc. – 855-542-7764 (Stamford and Bridgeport). Provides dual-diagnosis OP treatment including individual, group, and medications.

Multicultural Ambulatory Addiction Services – 203-495-7710, 426 East St., New Haven. Outpatient services, including methadone, for African-American and Latino adults 18+ with opioid addictions. Walk-ins accepted.

South Central Rehabilitation Center – 203-503- 3318, 232 Cedar St., New Haven. Medically monitored withdrawal and triage facility for adults 18+, including Spanish-speakers. Available 24/7.

The Root Center for Advanced Recovery – 1-800-862-2181, Option #2. Offers evidence-based OP treatment, group, and medications. Multiple locations, hours vary.

Waterstone Counseling Centers – 203-245-0412, 86 Bradley Rd., Madison | 446 Blake St., New Haven | 317 Long Hill Rd., Groton. Offers OP treatment including individual, group, and medications. Accepts Medicaid and some private insurance. Hours vary.

Outpatient Resources

BHcare – 203-800-7177 (co-ed), Valley: 435 E. Main St., Ansonia | Shoreline: 14 Sycamore Way, Branford. Offers outpatient (OP) treatment, recovery coaching, IOP, medication for addiction treatment, and primary care and wellness services.

MCCA – 203-285-6475, 419 Whalley Ave., Ste. 300, New Haven (co-ed). Offers OP individual and group services, IOP, and specialized groups such as relapse prevention and anger management.

Progressive Institute – 203-816-6424, 2 Trap Falls Rd., Ste. 120, Shelton. Offers IOP/OP, individual counseling, recovery coaching, and medication for addiction treatment.

Recovery Services of CT – 203-421-6242, 71 Bradley Rd., Ste. 6, Madison. Offers IOP/OP and specialized groups such as early recovery group and relapse prevention.

Retreat Behavioral Health – 203-873-9768, 1 Long Wharf Dr., New Haven (co-ed). Offers IOP/OP, and specialized groups such as relapse prevention.

Substance Abuse Treatment Unit – 203-974-5777, 1 Long Wharf Dr., New Haven (co-ed). Offers evaluation and OP services for individuals, including Spanish-speakers and HIV+ individuals.

Wildwood Behavioral Health – 203-421-6372, 1250 Durham Rd., Madison. Offers IOP/OP, and relapse prevention services.

Yale New Haven Psychiatric Hospital, Outpatient – 203-688-9723, 425 George St., New Haven (co-ed). Offers OP and IOP services for adults. **Hamden location with evening hours – 203-688-9723.**

Other Community Resources

988 – National Suicide Prevention Hotline and Veteran's Crisis Line – Dial 988

Community Health Centers
- Connecticut Mental Health Center (CMHC) – 203-974-7713
- Fair Haven Community Health Center – 203-777-7411

Connecticut Alliance of Recovery Residences (CTARR)
www.ctrecoveryresidences.org. Listing of CTARR certified sober houses in CT.

CT 2-1-1 – Out of state: 1-800-203-1234

Domestic Violence & Sexual Assault
- CT Domestic Violence Hotline – 888-774-2900
- Sexual Assault Crisis Services – 888-999-5545

DMHAS Access Line and Transportation – 800-563-4086. 24/7 call center to facilitate access to substance use treatment. Transportation to medically managed withdrawal prioritized.

DMHAS Bed Availability – www.ctaddictionservices.com. Listing of available addiction services, withdrawal management, residential treatment, recovery houses, and sober houses.

Health Insurance
- Healthy Start (for pregnant women) – 203- 946-8187
- Husky Health (Medicaid) – 877-284-8759

Health Services
- Breast and Cervical Cancer Screenings – 860-509-8251
- Department of Social Services (DSS) – 855-626-6632
- DSS Protective Services for Elderly – 203-974-8027
- DSS Family Services – 888-385-4225

HIV/AIDS
- AIDS Project New Haven – 203-624-0947
- CT AIDS Drug Assistance Program – 855-805-4325
- Hispanos Unidos, Inc. – 203-781-0226

Mutual Help
- Alcoholics Anonymous – 866-783-7712
- Narcotics Anonymous – 800-627-3543
- CT Community for Addiction Recovery – 203-672-4115, 1435 Chapel St., New Haven
- www.smartrecovery.com – global community of mutual-support groups
- www.intherooms.com – access to online recovery support services

Taking Initiative Center – 203-389-2970, x1317, 514 Whalley Ave., New Haven. Drop-in center for people facing homelessness and/or recovering from addiction. Provides referrals, not treatment.

Transitions Clinic – Linkages with correctional partners to provide support and access to continuity of care including comprehensive primary care and behavioral health services.
- InterCommunity Health Care – 860-569-5900, x202, 16 Coventry St., Hartford
- Cornell Scott Hill Health Center – Appointment: 203-850-2449 or 203-492- 9763, 911 State St., New Haven/Thursday 1-5pm
  150 Sargent Dr., 2nd Floor, New Haven/Friday 8am-12:30pm

Veyo Medical Transportation – 855-478-7350. Provides medical transportation.

Continue on back

Appendix 8: BNI Trifold - Spanish

¿Qué es el trastorno por consumo de alcohol?

Al igual que las afecciones como la diabetes o hipertensión, el trastorno por consumo de alcohol (AUD por sus siglas en inglés) es una condición médica crónica. Cuando alguien tiene un trastorno por consumo de alcohol, ellos:

1. experimentan pérdida de control por el consumo de alcohol;
2. consumen alcohol a pesar de las consecuencias adversas;
3. tienen deseos de consumir alcohol.

Tratamientos se encuentran disponibles, los cuales incluyen servicios orientación y opciones médicas. Consulte con su profesional médico para averiguar más sobre sus opciones.

¿Cuál es el espectro de su consumo de alcohol?

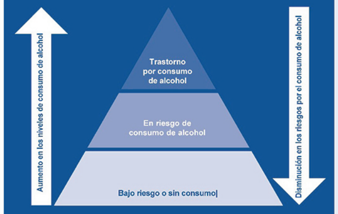

Disminuir su consumo de alcohol, o el evitar su consumo completamente, conllevará a una mejor condición general de salud y otras mejoras en su vida.

Recursos de tratamiento para individuos con trastornos de uso de sustancias y de salud mental

APT Foundation-Residential Services - 203-337-9943, 44-54 East Ramsdell St., New Haven (mixto). Ofrece servicios residenciales para adultos, incluso hispanohablantes, individuos VIH positivos, y aquellos con trastornos primarios de salud mental.

Aware Recovery Care - 203-631-2556. Ofrece tratamiento domiciliario de adicciones, abstinencia bajo supervisión médica, MAT, coaching para recuperación, psicoterapia, coordinación del cuidado/gestión de caso, y bienestar/educación familiar.

CASA - 203-339-4112, Bridgeport (mixto). Ofrece servicios residenciales intensivos e intermedios, programas ambulatorios (OP), ambulatorios intensivos (IOP), y de hospitalización parcial (PHP) para individuos hispanohablantes.

CHR-Milestone - 860-928-1860, Opción #1, Putnam (mujeres únicamente). Ofrece servicios residenciales intensivos para diagnóstico doble.

CHR-Roots to Recovery - 860-456-1769, Opción #5, Willimantic (hombres únicamente). Ofrece servicios residenciales a largo plazo/intermedios.

Connecticut Renaissance-McAuliffe Center - 203-346-1931, Waterbury (hombres únicamente). Ofrece servicios residenciales para diagnóstico doble.

Connecticut Valley Hospital-Blue Hills - 860-293-6400, Hartford (mixto). Ofrece servicios residenciales y manejo de abstinencia bajo supervisión médica.

Connecticut Valley Hospital-Merritt Hall - 800-828-3396, x5, Middletown (mixto). Ofrece servicios residenciales y manejo de abstinencia bajo supervisión médica.

First Step - 203-416-1915, Bridgeport (mixto). Ofrece manejo del síndrome de abstinencia bajo supervisión médica para adultos.

Grant Street Partnership - 203-503-3000, 60-62 Grant St., New Haven (hombres únicamente). Ofrece servicios IOP/OP (mayores de 18 años).

High Watch Recovery Center - 860-927-3772, 62 Carter Rd., Kent. Ofrece manejo del síndrome de abstinencia bajo supervisión médica, servicios residenciales, atención prolongada para vivir en sobriedad, y un IOP virtual.

InterCommunity Recovery Centers, Inc. - 860-569-5900, Hartford (mixto). Manejo de abstinencia bajo supervisión médica: x515 | Centro de rehab: x541. Ofrece manejo de abstinencia bajo supervisión médica, OP, IOP, tratamiento farmacológico, servicios residenciales intensivos e intermedios.

Liberation Programs - 855-542-7764, Bridgeport (mixto). Ofrece servicios residenciales a largo plazo/intermedios, manejo de abstinencia bajo supervisión médica, tratamiento MAT (Suboxona/ Naltrexona/ Metadona), IOP, y OP.

Midwestern CT Council of Alcoholism (MCCA) - 203-792-4515, Danbury (mixto). Ofrece servicios residenciales intensivos e intermedios, manejo de abstinencia bajo supervisión médica, tratamiento MAT (Suboxona/ Naltrexona), IOP, y OP.

Mountainside - 800-762-5433, x111, Canaan. Ofrece manejo de abstinencia bajo supervisión médica, servicios residenciales, atención prolongada para vivir en sobriedad, OP, grupo LGBTQ, y MAT (Suboxone/Vivitrol).

Regional Network of Programs (RNP)-Horizons - 203-333-3518, Bridgeport (mixto). Ofrece servicios residenciales intensivos.

Regional Network of Programs (RNP)-New Prospects - 203-610-6252, Bridgeport (mixto). Ofrece servicios residenciales para diagnóstico doble.

Retreat Behavioral Health - 203-873-9768, New Haven (mixto). Ofrece manejo de abstinencia bajo supervisión médica, servicios residenciales, MAT (Suboxone/Vivitrol), tanto OP como IOP.

Rushford Treatment Center - 877-577-3233, múltiples centros (mixto). Ofrece manejo de abstinencia bajo supervisión médica, servicios residenciales, MAT (Suboxone), PHP y OP para adultos y adolescentes.

SCADD - 860-447-1717, New London (mixto) | Lebanon (hombres únicamente). Ofrece manejo de abstinencia bajo supervisión médica y servicios residenciales a largo plazo para diagnóstico doble.

Silver Hill Hospital - 866-542-4455, New Canaan, (mixto). Ofrece manejo de abstinencia bajo supervisión médica y servicios residenciales para adolescentes y adultos.

Stonington Institute - 800-832-1022, North Stonington (mixto). Ofrece manejo de abstinencia bajo supervisión médica, servicios residenciales, PHP para diagnóstico doble con residencia para vivir en sobriedad e IOP.

IRB de Yale #2000031874

Promoción del compromiso con el tratamiento contra el consumo de alcohol posterior a la hospitalización

Proyecto ENHANCE

Mejora del tratamiento por consumo de alcohol iniciado en el hospital para aumentar compromiso

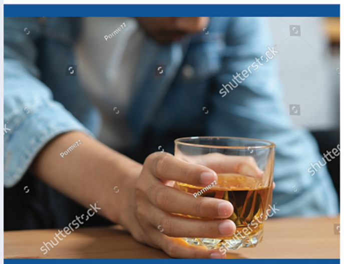

¿Qué es el trastorno por consumo de alcohol?

Yale

Proyecto ENHANCE

¿Qué es un trago estándar?

Un trago estándar se define como:

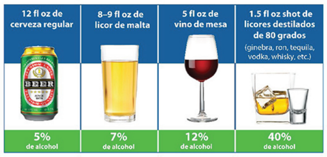

¿Cuáles son los límites de los tragos estándar para adultos?

El máximo de tragos por semana/evento

	Límite en un día	Límite semanal
Hombres de 65 años o menos	4 tragos estandarizados o menos	14 tragos estandarizados o menos
Mujeres u hombres de 65 años o mayores	3 tragos estandarizados o menos	7 tragos estandarizados o menos

¿Cuáles son los posibles problemas por el consumo de alcohol?

Estos son algunos de los problemas que pueden suceder con el consumo riesgoso de alcohol:

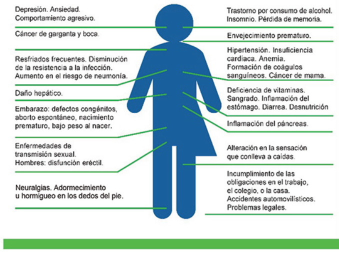

Programas que ofrecen tratamiento con medicamentos

APT Foundation - 203-781-4600, 1 Long Wharf Dr., Ste. 10, New Haven. Ofrece tratamiento OP individual, en grupos, IOP, y servicios de medicación. Servicios para hispanohablantes e individuos VIH+. No se requiere cita, pruebas de detección sin cita previa disponibles de lunes a viernes de 8:30 a 11 am.

Bridges Healthcare, Inc. - 203-878-6365, 949 Bridgeport Ave., Milford. Programa OP para diagnóstico doble. Ofrece grupos diurnos y nocturnos, IOP, y medicación. Acepta Medicaid, Medicare, y BCBS.

Bridges Healthcare: Mobile Addiction Treatment Team (MATT's van) – 203-494-5811. Ofrece medicación y servicios de coaching para recuperación destinados a adultos mayores de 18 años. No se requiere seguro. El programa/horario varía según las sedes.

CSHHC - Addiction Recovery Clinic - 203-503-3000, 150 Sargent Dr., New Haven. Ofrece tratamiento ambulatorio y con medicación. Horario limitado: martes de 8 am-12 pm, viernes de 1 a 5 pm.

Fair Haven Community Health Clinic - 203-809-3511, 374 Grand Ave., New Haven. FHCHC es un programa integrado que ofrece atención médica y del comportamiento para adultos mayores de 18 años, LGBTQ+, y VIH+. Servicios bilingües. Proporciona tratamiento con medicación y del comportamiento para trastornos por consumo de sustancias.

Liberation Programs, Inc. - 855-542-7764 (Stamford y Bridgeport). Proporciona tratamiento OP individual, en grupo y con medicamentos para diagnóstico dual.

Multicultural Ambulatory Addiction Services - 203-495-7710, 426 East St., New Haven. Servicios ambulatorios, que incluyen metadona, para afroamericanos y latinos adultos, mayores de 18 años con adicciones a opioides. Se aceptan consultas sin cita.

South Central Rehabilitation Center - 203-503- 3318, 232 Cedar St., New Haven. Manejo de abstinencia bajo supervisión médica e instalación de triaje para adultos mayores de 18 años de edad, incluso hispanohablantes. Disponible 24 horas al día los 7 días de la semana.

The Root Center for Advanced Recovery - 1-800-862-2181, Opción #2. Ofrece tratamiento OP según la evidencia, en grupo, y con medicamentos. Múltiples centros, y el horario varía.

Waterstone Counseling Centers - 203-245-0412, 86 Bradley Rd., Madison | 446 Blake St., New Haven | 317 Long Hill Rd., Groton. Ofrece tratamiento OP individual, en grupo y con medicamentos. Acepta Medicaid y algunos seguros privados. El horario de atención varía.

Recursos ambulatorios

BHcare - 203-800-7177 (mixto), Valley: 435 E. Main St., Ansonia | Shoreline: 14 Sycamore Way, Branford. Ofrece tratamiento ambulatorio (OP), coaching para la recuperación, IOP, medicación para el tratamiento de adicciones, y atención primaria y servicios de bienestar.

MCCA - 203-285-6475, 419 Whalley Ave., Ste. 300, New Haven (mixto). Ofrece servicios OP individuales y en grupo, grupos especializados como la prevención de recaídas y manejo de la ira.

Progressive Institute - 203-816-6424, 2 Trap Falls Rd., Ste. 120, Shelton. Ofrece IOP/OP, orientación individual, coaching para la recuperación, y medicación para el tratamiento de adicciones.

Recovery Services of CT - 203-421-6242, 71 Bradley Rd., Ste. 6, Madison. Ofrece IOP/OP y grupos especializados como el grupo de recuperación temprana y de prevención de recaídas.

Retreat Behavioral Health - 203-873-9768, 1 Long Wharf Dr., New Haven (mixto). Ofrece IOP/OP y grupos especializados como el de prevención de recaídas.

Substance Abuse Treatment Unit - 203-974-5777, 1 Long Wharf Dr., New Haven (mixto). Ofrece servicios de evaluación y OP para individuos, incluso hispanohablantes y VIH+.

Wildwood Behavioral Health - 203-421-6372, 1250 Durham Rd., Madison. Ofrece IOP/OP, y servicios para la prevención de recaídas.

Yale New Haven Psychiatric Hospital, Ambulatorio - 203-688-9723, 425 George St., New Haven (mixto). Ofrece servicios OP y de IOP para adultos. **Centro Hamden con horario nocturno - 203-688-9723.**

Otros recursos comunitarios

988 - Línea nacional de prevención del suicidio y línea de crisis para veteranos - Marcar 988

Centros de salud comunitarios
- **Connecticut Mental Health Center (CMHC) - 203-974-7713**
- **Fair Haven Community Health Center - 203-777-7411**

Alianza de Connecticut de residencias de recuperación (CTARR)
www.ctrecoveryresidences.org. Listado de casas para vivir en sobriedad certificadas por CTARR.

CT 2-1-1 - Fuera del estado: **1-800-203-1234**

Violencia doméstica y agresión sexual
- **Línea de atención de violencia doméstica de CT (CT Domestic Violence Hotline) - 888-774-2900**
- **Servicios de crisis por agresión sexual (Sexual Assault Crisis Services) - 888-999-5545**

Línea de acceso y transporte DMHAS - 800-563-4086. Centro de atención telefónica 24/7 para facilitar el acceso a tratamiento por abuso de sustancias. Transporte priorizado para manejo de abstinencia monitoreado médicamente.

Disponibilidad de camas DMHAS - www.ctaddictionservices.com. Listado de servicios de adicción, manejo de la abstinencia, tratamiento residencial, centros de rehabilitación, y de sobriedad.

Seguro de salud
- **Healthy Start** (para mujeres embarazadas) **- 203- 946-8187**
- **Husky Health** (Medicaid) **- 877-284-8759**

Servicios de salud
- **Pruebas para la detección de cáncer cervical y de mama - 860-509-8251**
- **Departamento de servicios sociales (Department of Social Services, DSS) - 855-626-6632**
- **Servicios de protección del adulto mayor del DDS (DSS Protective Services for Elderly) - 203-974-8027**
- **Servicios familiares del DSS (DSS Family Services) - 888-385-4225**

VIH/SIDA
- **Proyecto de SIDA de New Haven (AIDS Project New Haven) - 203-624-0947**
- **Programa de asistencia para medicamentos contra el SIDA de CT (CT AIDS Drug Assistance Program) - 855-805-4325**
- **Hispanos Unidos, Inc. - 203-781-0226**

Ayuda mutua
- **Alcohólicos Anónimos - 866-783-7712**
- **Narcóticos Anónimos - 800-627-3543**
- **Comunidad de CT para la recuperación de adicciones (CT Community for Addiction Recovery) - 203-672-4115, 1435 Chapel St., New Haven**
- **www.smartrecovery.com -** comunidad global de grupos de apoyo mutuo
- **www.intherooms.com -** acceso a servicios en línea de apoyo para recuperación

Taking Initiative Center - 203-389-2970, x1317, 514 Whalley Ave., New Haven. Centro de atención de acceso voluntario para personas sin hogar y/o recuperación de una adicción. Brinda derivaciones, y no tratamiento.

Transitions Clinic - Conexiones con socios penitenciarios para brindar apoyo y acceso a la continuidad de la atención que incluye servicios de atención primaria integral y de salud mental.
- **InterCommunity Health Care - 860-569-5900, x202, 16 Coventry St., Hartford**
- **Cornell Scott Hill Health Center -** Citas: **203-850-2449** o **203-492- 9763, 911 State St., New Haven**/jueves de 1 a 5 pm
  150 Sargent Dr., 2nd Floor, New Haven/viernes de 8 am-12:30 pm

Veyo Medical Transportation - 855-478-7350. Proporciona transporte médico.

Continúa al respaldo

Appendix 9: MAUD Trifold - English*

Medication Options for Alcohol Use Disorder Treatment

Medications can help individuals with an alcohol use disorder to:

- Regain a stable state of mind, free from the highs and lows that can come from alcohol use.
- Provide freedom from thinking about alcohol.
- Reduce craving for alcohol.
- Focus on healthy lifestyle.

Taking medication for alcohol use disorder is like taking medication to treat any other medical condition. These medications are safe and can be taken for years, if needed.

Reminders:

- Similar to medications for high blood pressure or diabetes, medications for alcohol use disorder can be prescribed by any clinican.
- Medications can be used in combination with other treatments like mutual support, but can also be used without other treatments.
- Some medications (except disulfiram) can be started or continued while drinking alcohol.

Speak with your clinician today about starting medication before your hospital discharge.

Project ENHANCE
ENhancing Hospital-initiated Alcohol TreatmeNt to InCrease Engagement

Yale IRB #2000031874

Yale

Promoting Alcohol Treatment Engagement Post-Hospitalization

Project ENHANCE
ENhancing Hospital-initiated Alcohol TreatmeNt to InCrease Engagement

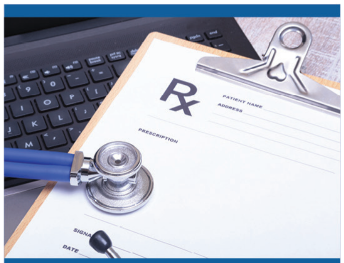

Medication Options for Alcohol Use Disorder Treatment

Yale

	Acamprosate (a-kam'-pro-sate)	Disulfiram (dye-sul'-fi-ram)	Naltrexone (nal-trex'-one)	Topiramate (toe-pyre'-a-mate)
**Description**	▪ Repairs chemical imbalance in systems of the brain responsible for excitation<br>▪ Decreases the number of relapses	▪ Helps people avoid alcohol by causing unpleasant effects when alcohol is consumed:<br>– Flushing in the face<br>– Throbbing headache<br>– Difficulty breathing<br>– Nausea<br>– Vomiting<br>– Sweating<br>▪ Does not decrease alcohol cravings *Those taking it should avoid alcohol in all forms including mouthwash and over the counter medications*	▪ Decreases or completely blocks the enjoyable effects of drinking<br>▪ Decreases the number of relapses	▪ Decreases craving for alcohol<br>▪ Repairs chemical imbalance in systems of the brain responsible for excitation and reward
**Potential Side Effects**	▪ Diarrhea<br>▪ Nervousness<br>▪ Weakness<br>▪ Difficulty falling or staying asleep<br>▪ Depression*<br>▪ Suicidal thoughts*<br>*CONTACT YOUR CLINICIAN IMMEDIATELY IF YOU EXPERIENCE THIS SIDE EFFECT	▪ Sleepiness<br>▪ Metallic taste<br>▪ Headache<br>▪ Yellowness of the skin or eyes*<br>▪ Numbness or tingling in hands and feet*<br>▪ Confusion*<br>▪ Losing contact with reality*<br>▪ Excessive and severe reaction when alcohol is consumed*<br>*CONTACT YOUR CLINICIAN IMMEDIATELY IF YOU EXPERIENCE THIS SIDE EFFECT	▪ Nausea<br>▪ Vomiting<br>▪ Injection site reaction *(hardening, itching, or swelling)*<br>▪ Headache<br>▪ Dizziness<br>▪ Nervousness<br>▪ Feeling tired<br>▪ Feeling sleepy<br>▪ Decreased appetite<br>▪ Yellowness of the skin or eyes*<br>▪ Allergic pneumonia with difficulty breathing, coughing, or wheezing*<br>▪ Suicidal thoughts*<br>*CONTACT YOUR CLINICIAN IMMEDIATELY IF YOU EXPERIENCE THIS SIDE EFFECT	▪ Abnormal tingling<br>▪ Nervousness<br>▪ Feeling tired<br>▪ Poor coordination<br>▪ Sleepiness<br>▪ Pain in belly<br>▪ Reduced appetite<br>▪ Poor memory*<br>▪ Confusion*<br>▪ Slowing of movements*<br>▪ Difficulty with concentration*<br>▪ Difficulty finding exact words*<br>▪ Suicidal thoughts*<br>*CONTACT YOUR CLINICIAN IMMEDIATELY IF YOU EXPERIENCE THIS SIDE EFFECT

WARNING

- Let your clinician know of any allergies or existing health conditions.
- Some patients cannot take certain medications because of health conditions or the medications may not mix well with others.
- Avoid driving or other activities where you need to be alert or see clearly until you know your reaction to the medication.
- Tell your clinician if you are pregnant, planning on getting pregnant, or breastfeeding.
- Tell your clinician if you experience any side effects.
- When prescribed disulfiram, avoid drinking alcohol, taking other medications that contain alcohol, and eating food containing alcohol.

*Indicates adapted from content created by Veterans Affairs, available at https://www.healthquality.va.gov/guidelines/MH/sud/MedicationsForTheTreatmentOfAlcoholUseDisorderBrochure92816.pdf

Appendix 9: MAUD Trifold - Spanish*

Opciones de medicación para el tratamiento del trastorno por consumo de alcohol

Los medicamentos pueden ayudar a los individuos con trastorno por consumo de alcohol a:

- Recuperar un estado mental estable, sin los altibajos que pueden originarse por el consumo de alcohol.
- Dejar de recurrir al uso de alcohol.
- Reducir el deseo intenso de consumir alcohol.
- Enfocarse en un estilo de vida sano.

Tomar medicación para el trastorno por consumo de alcohol es como tomar medicamento para tratar cualquier otra afección médica. Estos medicamentos son seguros y se puede tomar durante años, en caso de ser necesario.

Recordatorios:

- Al igual que los medicamentos para la hipertensión o diabetes, la medicación para el trastorno por consumo de alcohol se puede prescribir por cualquier médico.
- Los medicamentos se pueden utilizar en combinación con otros tratamientos como apoyo mutuo, pero también se pueden utilizar sin otros tratamientos.
- El uso de algunos medicamentos (excepto disulfiram) se puede iniciar o continuar mientras se consume alcohol.

Consulte con su médico hoy sobre el inicio del tratamiento con medicamentos antes de ser dado de alta del hospital.

Project ENHANCE
ENhancing Hospital-initiated Alcohol TreatmeNt to InCrease Engagement

IRB de Yale #2000031874

Yale

Promoción del compromiso con el tratamiento contra el consumo de alcohol posterior a la hospitalización

Proyecto ENHANCE
Mejora del tratamiento por consumo de alcohol iniciado en el hospital para aumentar compromiso

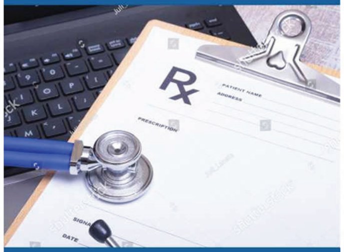

Opciones de medicación para el tratamiento por consumo de alcohol

Yale

	Acamprosato (a-kam'-pro-sato)	Disulfiram (di-sul'-fi-ram)	Naltrexona (nal-trex'-ona)	Topiramato (to-pir'-a-mato)
**Descripción**	• Restaura el desequilibrio químico en los sistemas del cerebro responsables de la excitación<br>• Disminuye el número de recaídas	• Ayuda a las personas a evitar el alcohol al provocar efectos desagradables cuando se consume alcohol:<br>- Enrojecimiento en la cara<br>- Dolor de cabeza palpitante<br>- Dificultad para respirar<br>- Náuseas<br>- Vómitos<br>- Transpiración<br>• No disminuye el deseo intenso de consumir alcohol *Aquellos que toman este medicamento deben evitar el alcohol en todas sus formas, incluso los enjuagues bucales y medicamentos de venta libre*	• Disminuye o bloquea completamente los efectos placenteros de consumir alcohol<br>• Disminuye el número de recaídas	• Disminuye el deseo intenso de consumir alcohol<br>• Restaura el desequilibrio químico en los sistemas del cerebro responsables de la excitación y recompensa
**Posibles efectos secundarios**	• Diarrea<br>• Nerviosismo<br>• Debilidad<br>• Dificultad para conciliar el sueño o permanecer dormido<br>• Depresión*<br>• Pensamientos suicidas*<br>*PÓNGASE EN CONTACTO CON SU MÉDICO DE INMEDIATO SI EXPERIMENTA ESTE EFECTO SECUNDARIO	• Somnolencia<br>• Sabor metálico<br>• Dolor de cabeza<br>• Color amarillo de la piel o los ojos*<br>• Entumecimiento u hormigueo en pies y manos*<br>• Confusión*<br>• Pérdida de contacto con la realidad*<br>• Reacción excesiva y grave cuando se consume alcohol*<br>*PÓNGASE EN CONTACTO CON SU MÉDICO DE INMEDIATO SI EXPERIMENTA ESTE EFECTO SECUNDARIO	• Náuseas<br>• Vómitos<br>• Reacción en el lugar de la inyección *(endurecimiento, picazón, o hinchazón)*<br>• Dolor de cabeza<br>• Mareo<br>• Nerviosismo<br>• Sensación de cansancio<br>• Sensación de somnolencia<br>• Disminución del apetito<br>• Color amarillo de la piel o los ojos*<br>• Neumonía alérgica con dificultad para respirar, tos, o respiración sibilante*<br>• Pensamientos suicidas*<br>*PÓNGASE EN CONTACTO CON SU SU MÉDICO DE INMEDIATO SI EXPERIMENTA ESTE EFECTO SECUNDARIO	• Hormigueo anormal<br>• Nerviosismo<br>• Sensación de cansancio<br>• Coordinación deficiente<br>• Somnolencia<br>• Dolor de estómago<br>• Disminución del apetito<br>• Mala memoria*<br>• Confusión*<br>• Lentitud en los movimientos*<br>• Dificultad para concentrarse*<br>• Dificultad para encontrar las palabras exactas*<br>• Pensamientos suicidas*<br>*PÓNGASE EN CONTACTO CON SU MÉDICO DE INMEDIATO SI EXPERIMENTA ESTE EFECTO SECUNDARIO

ADVERTENCIA

- Infórmele a su médico de cualquier alergia o afección de salud existente.
- Algunos pacientes no pueden tomar ciertos medicamentos debido a sus afecciones de salud o no se mezclan bien con otros.
- Evite conducir u otras actividades donde necesite estar alerta o ver claramente hasta que conozca su reacción al medicamento.
- Infórmele a su médico si está embarazada, planea quedar embarazada, o está lactando.
- Dígale a su médico si experimenta alguno de estos efectos secundarios.
- Cuando se le prescriba disulfiram, evite el consumo de alcohol, tomar otros medicamentos que contengan alcohol, y comer alimentos que contengan alcohol.

*Indicates adapted from content created by Veterans Affairs, available at https://www.healthquality.va.gov/guidelines/MH/sud/MedicationsForTheTreatmentOfAlcoholUseDisorderBrochure92816.pdf

Appendix 10: CBT4CBT Trifold - English

What is Cognitive Behavioral Therapy (CBT)?

CBT is a type of talk therapy that is helpful at addressing alcohol and drug use. It is based on the idea that thoughts and feelings can lead to unhelpful behaviors. CBT helps you change thinking patterns and learn new coping skills.

What is CBT4CBT?

CBT4CBT stands for "Computer-Based Training For Cognitive Behavioral Therapy". It is a computer-based program to help you learn skills and strategies for reducing or stopping alcohol use. Rather than a counselor teaching you these skills, you will learn them on your own through an interactive computer program.

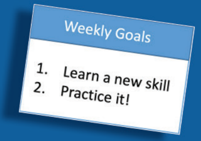

Project ENHANCE
ENhancing Hospital-initiated Alcohol TreatmeNt to InCrease Engagement

Yale IRB #2000031874

Yale

Promoting Alcohol Treatment Engagement Post-Hospitalization

Project ENHANCE
ENhancing Hospital-initiated Alcohol TreatmeNt to InCrease Engagement

CBT4CBT

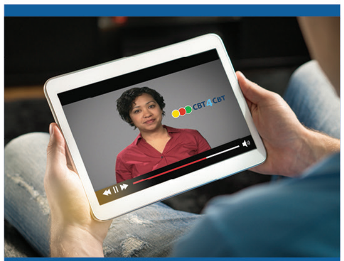

Computer-Based Training For Cognitive Behavioral Therapy

Yale

Project ENHANCE

CBT4CBT will teach you how to RECOGNIZE, AVOID, and COPE.

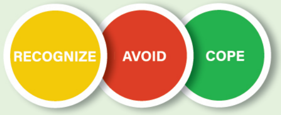

- **RECOGNIZE** your triggers and the things that set you up to drink.
- **AVOID** triggers and situations that involve alcohol.
- **COPE** with triggers, such as stress and negative emotions, in a healthy way.

How long is the CBT4CBT program?

There are 7 modules (or lessons). Each lesson takes about 30-45 minutes.

The modules will teach you how to:

1 – Recognize triggers and patterns of drinking
2 – Manage cravings and cope with urges to drink
3 – Say "no" to offers for alcohol
4 – Problem solve
5 – Identify and change negative thoughts
6 – Make safer decisions
7 – Prevent relapse

How does it work?

CBT4CBT uses movies and interactive exercises to teach skills.

A narrator will guide you through skills.

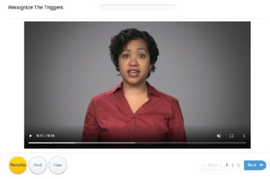

All you have to do is press a button and the program guides you through

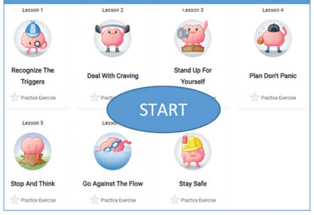

Is it safe? *YES*

- It is completely safe and confidential.
- The program collects no personal information about you.

How do I access it?

You can access **CBT4CBT** from any device, such as a smartphone, tablet, laptop, or computer.

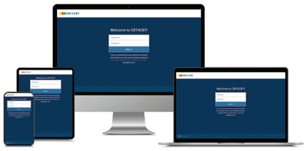

Go to: **home.cbt4cbt.com** or scan the QR code with your phone camera.

A tablet or other device with a screen size larger than 10 inches is recommended for optimal viewing.

SCAN ME

Appendix 10: CBT4CBT Trifold - Spanish

¿Qué es la Terapia Cognitivo Conductual (TCC)?

La TCC es un tipo de terapia conversacional que es útil para el consumo de alcohol y drogas. Se basa en la idea de que los pensamientos y sentimientos pueden conducir a comportamientos improductivo La TCC lo ayuda a cambiar los patrones de pensamiento y aprender nuevas habilidades de afrontamiento.

¿Qué es CBT4CBT?

CBT4CBT significa "Entrenamiento basado en computadora para terapia cognitiva conductual". Es un programa basado en computadora para ayudarlo a aprender nuevas habilidades y estrategias para reducir o detener el consumo de alcohol. En lugar de que un consejero le enseñe estas habilidades, las aprenderá por su cuenta a través de un programa de computadora interactivo.

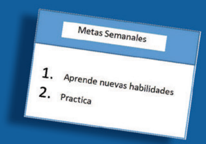

Project ENHANCE
ENhancing Hospital-initiated Alcohol TreatmeNt to InCrease Engagement

Yale IRB #2000031874

Yale

Promoción de participación en tratamiento del alcoholismo después de hospitalización

Project ENHANCE
ENhancing Hospital-initiated Alcohol TreatmeNt to InCrease Engagement

CBT4CBT

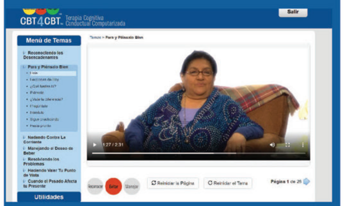

Entrenamiento computacional de terapia cognitiva

Yale

Project ENHANCE

CBT4CBT Te enseñará a RECONOCER, EVITAR, y MANEJAR.

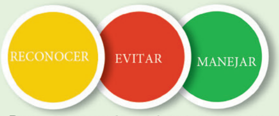

- **RECONOCER** tus desencadenantes y cosas que te llevan a beber triggers and the things that set you up to drink.
- **EVITAR** desencadenantes y situaciones que llevan al consumo del alcohol
- **MANEJAR** con los desencadenates como estres, y emociones negatiovas, de una manera saludable .

¿Cuánto dura el programa CBT4CBT?

Hay 7 módulos (o lecciones). Cada lección dura entre 30 y 45 minutos.

Los módulos le enseñarán cómo:

1- Reconocer desencadenantes y patrones de consumo de alcohol
2- Manejar los antojos y hacer frente a los impulsos beber
3- Decir "no" a las ofertas de alcohol
4 – Resolución de problemas
5 – Identificar y cambiar pensamientos negativos
6 – Tomar decisiones más seguras
7 – Prevenir la recaída

¿Como funciona?

CBT4CBT usa una telenovelas y ejercicios interactivos para enseñarte habilidades.

Una narradora te guiará a través del programa

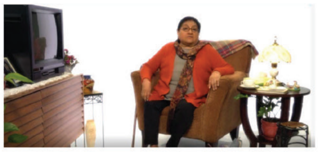

Todo lo que tienes que hacer es presionar un botón y el programa te guiará.

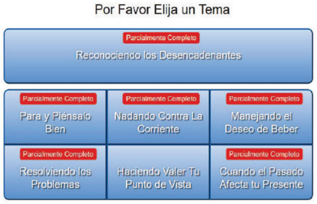

¿Es seguro? *Si*

- Es completamente seguro y confidencial.
- El programa no recopila información personal sobre usted

¿Cómo accedo el programa?

Puede acceder a CBT4CBT desde cualquier dispositivo, como un teléfono inteligente, una tableta, una computadora portátil o una computadora.

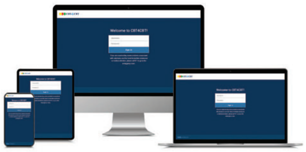

Vaya a: **https://app.cbt4cbt.org** o escanee el código QR con la cámara de su teléfono.

Se recomienda una tableta u otro dispositivo con una pantalla de más de 10 pulgadas para una visualización óptima.

SCAN ME

Escanéame

Appendix 11: Clinician Educational Materials *(page 1 of 4)*

Project ENHANCE

ENhancing **H**ospital-initiated **A**lcohol Treatme**N**t to In**C**rease **E**ngagement

How do you diagnosis alcohol use disorder (AUD)?

- Based on the Diagnostic and Statistical Manual 5th edition (DSM-5), an alcohol use disorder is present based on the presence of 2 or more of the following 11 criteria in the prior 12 months:

These criteria reflect manifestations of the 3Cs

Craving
- Craving
- Tolerance
- Withdrawal

Loss of Control
- Larger quantity over longer period of time
- Unsuccessful attempts to cutback or control
- Increased time spent

Consequences
- Failure to fulfill major role obligations
- Social/interpersonal problems
- Activities given up
- Use in hazardous situations
- Physical and psychological consequence

Severity: Mild=2-3 criteria; Moderate=4-5 criteria; Severe=6 or more criteria

Why should I prescribe medications for AUD (MAUD)?

- Based on strong evidence to support their use, MAUD are recommended by clinical guidelines to help prevent return to heavy drinking (defined for women as >4 drinks on any day or >8 drinks/week, and for men as >5 drinks on any day or >15 drinks/week).
- Less than 2% of individuals with an AUD receive a Food and Drug Administration approved medication.
- No special training is required to prescribe MAUD.
- Upon hospital discharge, patients can receive ongoing treatment and follow-up in Primary Care and/or Addiction Specialty settings.

Yale

Yale IRB #2000031874

Appendix 11: Clinician Educational Materials *(page 2 of 4)*

Project ENHANCE

What are recommended medications for AUD?

Medication	Mechanism	Side Effects	Notes
**Naltrexone**<br>▪ oral 50-100mg daily<br>▪ injectable 380mg monthly	Opioid antagonist that may reduce subjective reward associated with alcohol use.	Nausea, indigestion, headache, fatigue. Depressive symptoms. Rarely medication-associated hepatitis. Potential for precipitated opioid withdrawal if opioids present.	Contraindicated in the presence of opioid use. Use with caution with acute hepatitis or Child Pugh class B. Weigh risks versus benefits for patients with Child Pugh Class C liver disease.<br>If unable to get liver function tests, this should NOT delay starting naltrexone.
**Acamprosate**<br>▪ 666mg 3 times daily	May antagonize glutamate-mediated neuronal hyperexcitability and reduce prolonged (but not acute) withdrawal symptoms.	Diarrhea, nausea/vomiting, myalgia, rash, dizziness, palpitations. Rarely associated with renal impairment.	Reduced dosage with renal insufficiency. May be used with naltrexone. Medication adherence may be challenging.
**Disulfiram**<br>▪ 250-500mg daily	Aldehyde dehydrogenase inhibition results in acetaldehyde accumulation with alcohol use, leading to unpleasant symptoms (i.e., alcohol-disulfiram reaction).	Drowsiness, rash. Rarely medication-associated severe hepatotoxicity, optic neuritis, peripheral neuropathy.	Potential for many medication interactions. Patient must be abstinent >48 hours before administration. Avoid in patients with hepatic impairment or cardiovascular disease. Most appropriate for patients with strong motivation to be abstinent and with support to promote medication adherence.
**Topiramate***<br>▪ 25-150mg twice daily	Modulates GABA and antagonizes glutamate receptors.	Dizziness, drowsiness, fatigue, anorexia; Cognitive dysfunction including memory impairment, impaired attention, decreased processing speed and verbal fluency; anxiety, depression; paresthesia; non-anion gap metabolic acidosis, nephrolithiasis	Titrate to initiate. Requires dose adjustment for kidney impairment. Use lower dose with liver impairment. Consider when co-occurring diagnosis present including cocaine use disorder, chronic migraine, seizure disorder, PTSD.

*Not currently approved by Food and Drug Administration for this indication, but recommended by clinical guidelines

Yale

Page 2 of 4

Yale IRB #2000031874

Appendix 11: Clinician Educational Materials *(page 3 of 4)*

Project ENHANCE

What are common misconceptions and facts regarding MAUD?

Misconception	Fact
A patient's goal must be abstinence from alcohol use to start a medication	Naltrexone can be safely used for reducing heavy drinking days
The risk of liver injury is a strong contraindication to naltrexone for AUD	Patients with liver disease can safely use many medications for AUD and the benefits outweigh the risks
Patients unlikely to adhere to or follow post discharge care should not receive medications	Medications are safe to start in patients who may not be retained in care
Injectable naltrexone requires Addiction Medicine or Psychiatry consult	Injectable naltrexone may be prescribed by the primary team; all patients, including those who receive injectable naltrexone, should have a post-hospital discharge follow-up plan
Special training or a waiver is required to prescribe MAUD	All clinicians can prescribe these medications and NO special training or waiver is required

What should I offer patients upon hospital discharge?

- Patients should be provided enough MAUD to last them to their post-hospital discharge follow-up appointment.
  - For patients who receive injectable naltrexone in the hospital, they should be scheduled for follow-up within 30 days of their injection.
  - For patients who are prescribed an oral medication, they should be prescribed enough days to last them to their appointment up to 30 days post-discharge.
- Ensure all patients have a follow-up appointment with date, time, and place for appropriate care.

Yale
Page 3 of 4
Yale IRB #2000031874

Appendix 11: Clinician Educational Materials *(page 4 of 4)*

Project ENHANCE

What are other resources regarding MAUD and AUD treatment?

Local resources:

- Yale Addiction Medicine Consult Service
- Epic Care Signature Pathway for Alcohol Use Disorder Treatment Initiation: Adult Inpatient

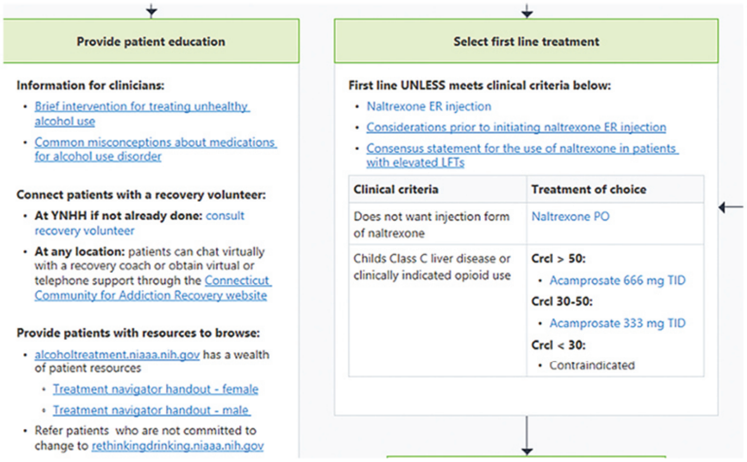

Other resources:

- National Institute on Alcohol Abuse and Alcoholism Healthcare Professional's Core Resource on Alcohol
- Substance Abuse and Mental Health Services Administration Medication for the Treatment of Alcohol Use Disorder: A Brief Guide
- Monitoring for Liver Function Tests in Patients Receiving Naltrexone or Extended-Release Naltrexone
- VA/DoD Clinical Practice Guidelines
- American Psychiatric Association Practice Guideline for the Pharmacological Treatment of Patients with Alcohol Use Disorder

Yale IRB #2000031874

Appendix 8: SUP_Intervention Adherence Rating Form

Please complete the survey below. Thank you!

This Survey is for Record ID [record_id] . The participant was randomized to [rand]			
INSTRUCTIONS: Please check either YES, NO, or N/A (not applicable), or score one number between 1 and 7, where applicable, for each of the following items.			
**Did the Health Promotion Advocate (HPA)...**			
	NO	YES	N/A
1) Ask patient for permission to discuss alcohol use and pause for their response?	○	○	○
2) Review patient's drinking patterns and ask about patient's concern?	○	○	○
3) Use open-ended question to ask patient about a connection between drinking and current hospitalization?	○	○	○
4) Use reflections to make a specific connection between drinking and their current medical issue (e.g., pancreatitis, GI complaints, hypertension)?	○	○	○
5) Ask permission to share treatment options and reviewed options with patient?	○	○	○
6) Use the "Readiness Ruler" to facilitate discussion about readiness for treatment?	○	○	○
7) Negotiate a goal to engage in treatment?	○	○	○
8) Provide clear information for AUD treatment post-hospital discharge?	○	○	○
9) Tell patient in a confrontational manner, that they have to cut down?	○	○	○
10) Make suggestions regarding how much patient should cut down?	○	○	○
11) Refer to patient as an "alcoholic"?	○	○	○
12) Offer unsolicited advice?	○	○	○

Appendix 12: SUP_Intervention Adherence Rating Form *(continued)*

Did the Health Promotion Advocate (HPA)...							
	NO			YES			N/A
13) Provide and review "Project ENHANCE BNI trifold" Information sheet?	○			○			○
14) Provide and review "Project ENHANCE BNI+MAUD trifold"?	○			○			○
15) Provide and review "Project ENHANCE BNI+MAUD+CBT4CBT trifold"?	○			○			○
16) Obtain patient signature on alcohol use agreement and referral sheet?	○			○			○
17) Summarize the session at the end of encounter?	○			○			○
18) Schedule a 2-week phone booster session with patient?	○			○			○
19) Offer confrontational warnings regarding drinking?	○			○			○
	1<br>Not at all	2	3	4<br>Somewhat	5	6	7<br>Extensively
20) To what degree did the HPA reflect patient's motivational statements regarding cutting down?	○	○	○	○	○	○	○
21) To what degree did the HPA's reflective statements and open questions pull for change talk?	○	○	○	○	○	○	○
22) Re-direct non-motivational statements?	○	○	○	○	○	○	○

XII. References

[1] D'Onofrio G, Bernstein E, Rollnick S. Motivating patients for change: a brief strategy for negotiation. In: Bernstein E, Bernstein J (eds). Case studies in Emergency Medicine and the health of the Public. Boston: Jones and Bartlett, 1996, pp 295-303.

[2] Bernstein E, Bernstein J, Levenson S. Project ASSERT: an ED-based intervention to increase access to primary care, preventive services, and the substance abuse treatment system. Ann Emerg Med. 1996;30:181-189.

[3] D'Onofrio G, Pantalon MV, Degutis LC, Fiellin DA, O'Connor PG. Developmemnt and implementation of an emergency practitioner-performed brief intervention for hazardous and harmful drinkers in the emergency department. Acad Emerg Med 2005;12:249-256.

[4] Edelman EJ, Maisto SA, Hansen NB, et al. Integrated stepped alcohol treatment for patients with HIV and liver disease: A randomized trial. J Subst Abuse Treat. Nov 2019;106:97-106. doi:10.1016/j.jsat.2019.08.007

[5] Edelman EJ, Maisto SA, Hansen NB, et al. Integrated stepped alcohol treatment for patients with HIV and at-risk alcohol use: a randomized trial. Addict Sci Clin Pract. Jul 29 2020;15(1):28. doi:10.1186/s13722-020-00200-y

[6] Edelman EJ, Maisto SA, Hansen NB, et al. The Starting Treatment for Ethanol in Primary care Trials (STEP Trials): Protocol for Three Parallel Multi-Site Stepped Care Effectiveness Studies for Unhealthy Alcohol Use in HIV-Positive Patients. Contemp Clin Trials. Jan 2017;52:80-90. doi:10.1016/j.cct.2016.11.008

[7] Veterans Affairs, available at https://www.healthquality.va.gov/guidelines/MH/sud/MedicationsForTheTreatmentOfAlcoholUseDisorderBrochure92816.pdf

[8] Donroe JH, Edelman EJ. Alcohol use. Annals Intern Med. Oct 2022.
